# Trifaceted Mickey Mouse Amphiphiles for Programmable Self‐Assembly, DNA Complexation and Organ‐Selective Gene Delivery

**DOI:** 10.1002/chem.202100832

**Published:** 2021-05-26

**Authors:** Ana I. Carbajo‐Gordillo, Manuel González‐Cuesta, José L. Jiménez Blanco, Juan M. Benito, María L. Santana‐Armas, Thais Carmona, Christophe Di Giorgio, Cédric Przybylski, Carmen Ortiz Mellet, Conchita Tros de Ilarduya, Francisco Mendicuti, José M. García Fernández

**Affiliations:** ^1^ Institute for Chemical Research IIQ CSIC-Univ. Sevilla C/ Américo Vespucio 49 41092 Sevilla Spain; ^2^ Department of Organic Chemistry Faculty of Chemistry University of Sevilla C/ Prof García González 1 41012 Sevilla Spain; ^3^ Department of Pharmaceutical Technology and Chemistry School of Pharmacy and Nutrition University of Navarra 31080 Pamplona Spain; ^4^ Department of Analytical Chemistry Physical Chemistry and Chemical Engineering Instituto de Investigación Química “Andrés M. del Rio” (IQAR) University of Alcalá Campus Universitario Ctra. Madrid-Barcelona Km 33.600 28871 Alcalá de Henares Spain; ^5^ Institut de Chimie Nice UMR 7272 Université Côte d'Azur 28, Avenue de Valrose 06108 Nice France; ^6^ CNRS Institut Parisien de Chimie Moléculaire IPCM Sorbonne Université Paris France

**Keywords:** cyclooligosaccharides, macrocycles, molecular nanoparticles, non-viral gene delivery, self-assembling, trehalose

## Abstract

Instilling segregated cationic and lipophilic domains with an angular disposition in a trehalose‐based trifaceted macrocyclic scaffold allows engineering patchy molecular nanoparticles leveraging directional interactions that emulate those controlling self‐assembling processes in viral capsids. The resulting trilobular amphiphilic derivatives, featuring a Mickey Mouse architecture, can electrostatically interact with plasmid DNA (pDNA) and further engage in hydrophobic contacts to promote condensation into transfectious nanocomplexes. Notably, the topology and internal structure of the cyclooligosaccharide/pDNA co‐assemblies can be molded by fine‐tuning the valency and characteristics of the cationic and lipophilic patches, which strongly impacts the transfection efficacy in vitro and in vivo. Outstanding organ selectivities can then be programmed with no need of incorporating a biorecognizable motif in the formulation. The results provide a versatile strategy for the construction of fully synthetic and perfectly monodisperse nonviral gene delivery systems uniquely suited for optimization schemes by making cyclooligosaccharide patchiness the focus.

## Graphical abstract

**Mickey Mouse molecular nanoparticles** with a trehalose‐based cyclooligosaccharide core and uneven distributions of cationic (**C**, red lobules) and lipophilic patches (**L**, blue lobules) co‐assemble with plasmid DNA (**pDNA**, orange chains) to afford transfectious nanacomplexes with programmable topologies and internal structures, ranging from multilamellar spheroids and rods to core‐shell arrangements, translating into amazing cell efficiencies in vitro and organ selectivities in vivo with no need of targeting labels.

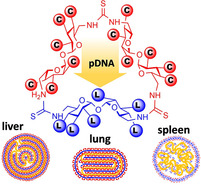

## Introduction

Surface anisotropy has proven to be vital in biological systems. An example is the well‐regulated structural control seen in virus capsids, which stems from the overall shape of the folded protein, the arrangement of hydrophobic areas on the protein surface and the distribution of charged residues, altogether setting up the spread of disease by self‐assembly of viral particles in vivo.^[1]^ In capsids, the interfaces responsible for the protein‐protein interactions must not only be in contact, but also have the appropriate relative orientation. It has long been the aim of chemists to instill artificial systems with similarly directional interactions in order to self‐assemble advanced materials capable of performing sophisticated tasks with limited human intervention.^[2]^ The basic notion is that heterogeneous arrangements of functional elements on particle or macromolecule surfaces, resulting in the formation of localized clusters or “patches”, can bestow the system with different features compared to those with uniformly distributed groups, translating into unique capabilities to self‐assemble into 2D and 3D structures of different topologies.^[3]^

**Figure 1:**
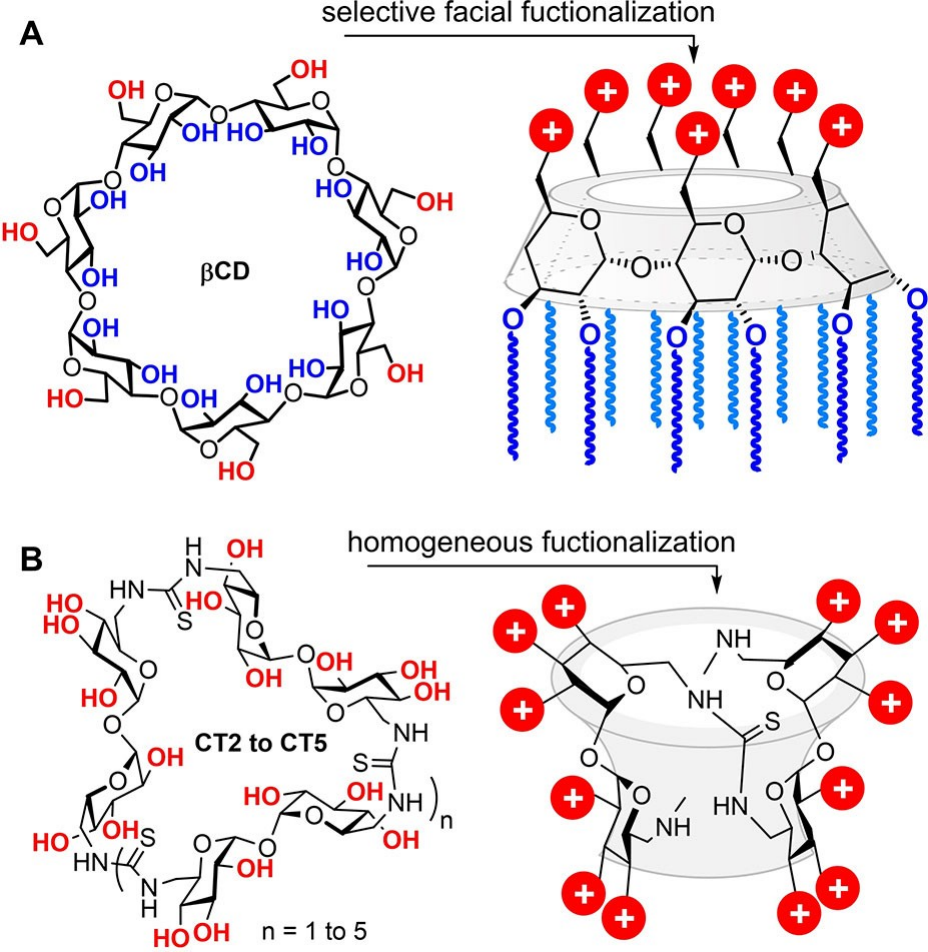
A) Structure of β‐cyclodextrin (βCD) and schematic representation of a Janus‐type polycationic amphiphilic derivative. B) General structure of cyclotrehalans (CTs) and schematic representation of polycationic CT‐centered star‐shape polymers.

The fabrication of patchy nanoconstructs that are highly monodisperse in patch number (valency), size and position represents a main defy for the above channels.^[4]^ Most reports focus on Janus bodies with two dissimilar hemispheres.^[5]^ Meanwhile, the development of efficient strategies for the elaboration of robust nanostructured objects with higher face valency, displaying well‐defined static patterns of segregated domains, remains a daunting endeavor.^[6]^ Molecular nanometric entities (molecular nanoparticles, MNPs) exhibiting defined symmetry and persistent shape and volume such as fullerenes, polyhedral oligomeric silsequioxanes (POSS), metal‐organic frameworks (MOFs), calixarenes, or cyclodextrins (CDs), in combination with precision synthesis methodologies, offer unique opportunities towards this end.^[7]^ The synthesis of gene delivery systems (vectors) based on monodisperse cyclodextrin (especially β‐cyclodextrin; βCD) derivatives is a paradigmatic example: both the self‐assembling properties and the abilities to form nanocomplexes with nucleic acids (CDplexes) are tightly dependent on the anisotropic disposition of clusterized cationizable and lipophilic groups at opposite rims of the macrocyclic core in a Janus‐like architecture (Figure 1A).^[8]^ Increasing the number of cationic or lipophilic patches is expected to broaden the opportunities to regulate directional recognition phenomena in search for artificial viruses. However, neither cyclodextrins nor any of the commonly used MNP platforms allows breaking the two‐face Janus boundary.^[9]^

Macrocyclic scaffolds based on the disaccharide α,α’‐trehalose (cyclotrehalans, CTs) typify a singular addition to the MNP cohort.^[10]^ CT representatives incorporating from two to five α,α’‐trehalose bricks (CT2 to CT5) are on record.^[11]^ Differently from CDs, which are intrinsically dissymmetric Janus molecules, CTs portray as many faces as the number of constitutive α,α’‐trehalose units, all of which bear six secondary hydroxyls and are chemically identical in the canonical CT representatives. Homogeneous functionalization of the CT2 (cyclotetrasaccharide) and CT3 (cyclohexasaccharide) members was put at work to access highly symmetrical polycationic star‐shape polymers (Figure 1B) that showed high gene delivery efficiencies.^[12]^

**Figure 2:**
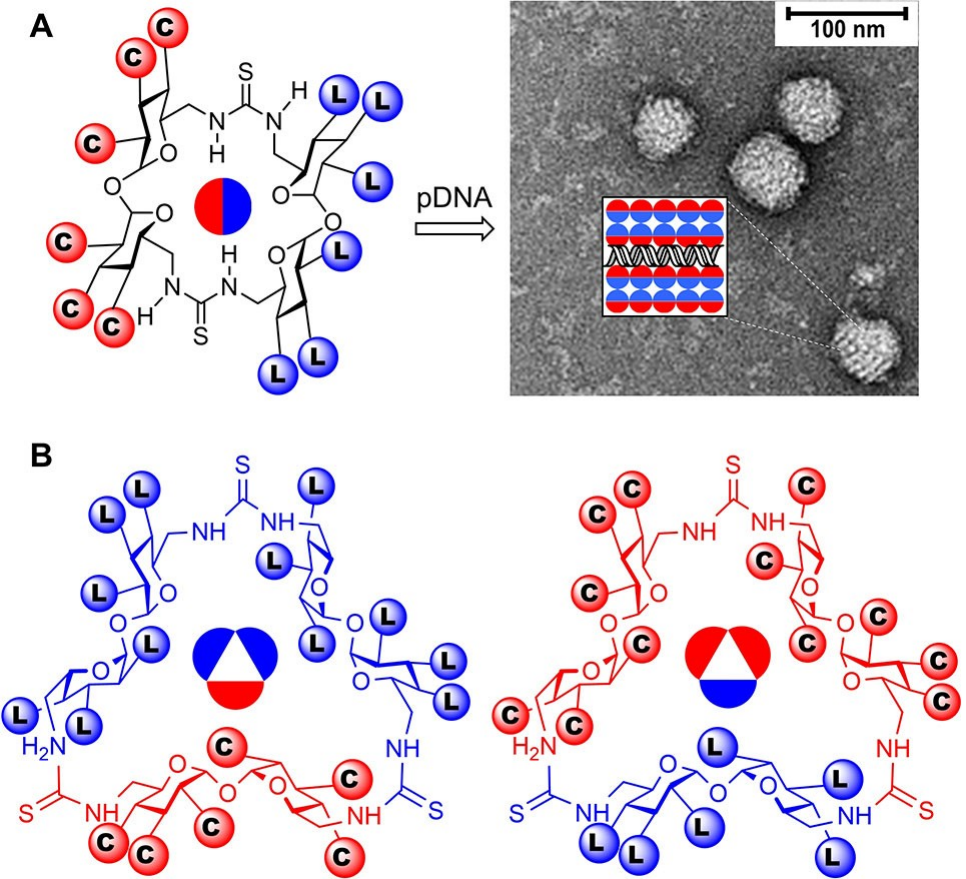
A) Previous work: Janus‐type CT2 derivatives (left) and representative TEM micrograph of the nanocomplexes formed upon co‐assembly with pDNA; a schematic representation of the layered ultrathin structure is shown in the insert (right).^[13]^ B) This work: structure of the new CT3‐based Mickey Mouse‐type molecular vectors with uneven distribution of cationic (C) and lipophilic domains (L).

**Scheme 1:**
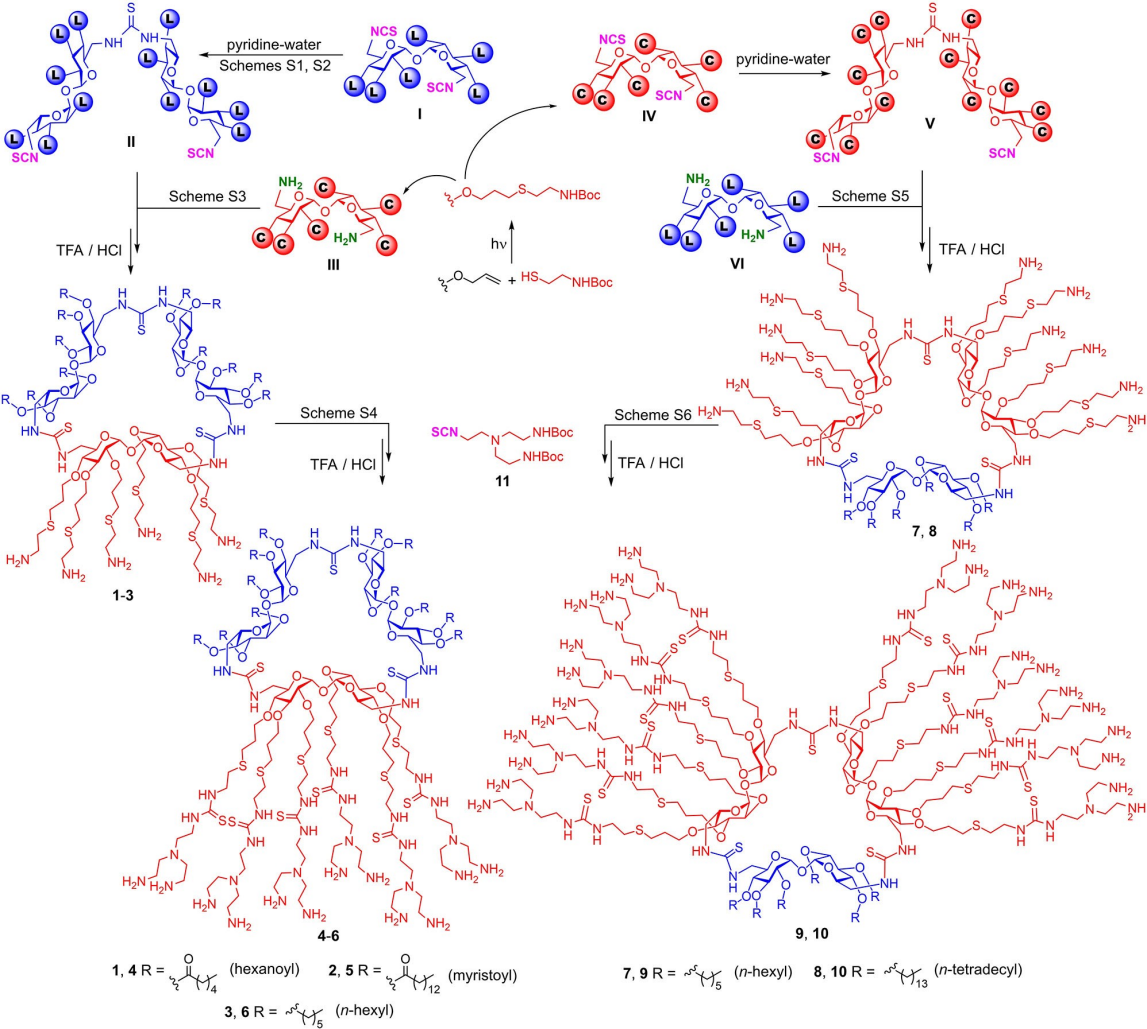
Synthesis of the C_1_L_2_ (left) and C_2_L_1_ (right) Mickey Mouse cyclotrehalans **1**–**6** and **7**–**10**.

Interestingly, CTs can potentially be constructed from differently functionalized building blocks, then resulting in anisotropically‐faceted architectures. This notion was first realized for the simplest CT2 core: perfect Janus MNPs featuring cationic (C) and lipophilic (L) halves were elaborated that formed multilamellar transfectious nanocomplexes (CTplexes) with plasmid DNA (pDNA) (Figure 2A).^[13]^ We hypothesized that increasing the face valency would enable new opportunities to encode information for programmable assembly. CTplex fine structure and topology could then be precast in order to optimize gene vector performance and selectivity. As a proof of concept, here we report the synthesis of patchy MNPs combining C and L lobes in 1 : 2 (C_1_L_2_) or 2 : 1 (C_2_L_1_) relationships with a clear‐cut angular disposition (Figure 2B). By analogy with the terminology coined for colloidal particles with the same type of patchiness, we call this new prototypes Mickey Mouse molecular nanoparticles (MM‐MNPs).^[14]^ The synthesis, characterization, supramolecular self‐assembling, pDNA nanocomplexation and in vitro and in vivo transfection capabilities of compounds **1**–**10** (Scheme 1) are discussed.

## Results and Discussion

### Design criteria and synthesis

One can anticipate that going from the Janus‐type CT2 pattern (Figure 2A) to the C_1_L_2_ trilobular CT3 organization (Figure 2B, left) would roughly multiply by two the volume of the hydrophobic area in the corresponding amphiphiles. Going to C_2_L_1_ MM‐MNPs (Figure 2B, right) will instead increase the effective area of the cationic domain. Such differences are expected to result in significantly disparate self‐assembling properties, as well as pDNA complexing and transfection capabilities, which might be further adjusted by tailoring the cationic and lipophilic appendages.^[15]^ In order to test this notion, we settled to develop efficacious and versatile methodologies, compatible with the facile elaboration of both the cationic and lipid domains, to access both types of Mickey Mouse patchy macrocycles for tunable gene delivery.

The key step in our synthetic scheme was the intermolecular macrocyclization reaction between a *C*_2_‐symmetric linear tetrasaccharide armed with isothiocyanate groups at the distal primary positions (structure **II** or **V**) and a 6,6’‐diamino‐6‐6’‐dideoxy‐α,α’‐trehalose partner (structure **III** or **VI**; Scheme 1).^[16]^ The formation of the CT3 core is then a self‐templated process driven by the rigid concave/convex geometry of the trehalose building blocks.^[17]^ The tetrasaccharide precursor was obtained from the homologous disaccharide diisothiocyanate (structure **I** or **IV**)^[16]^ by controlled self‐condensation in pyridine‐water, a reaction that provides the dimeric thiourea with no involvement of a transient amine, thus preventing O→N acyl migration side reactions.^[18]^ In the case of C_1_L_2_ CT3 derivatives, the dimeric diisothiocyanate (structure **II**) bears twelve lipophilic tails anchored at the secondary oxygen atoms through either ester (C_6_ or C_14_) or ether functionalities (C_6_), whereas the diamine (structure **III**) was equipped with six *O*‐linked *N*‐*tert*‐butoxycarbonyl (Boc)‐protected cysteaminylpropyl heads. The later were installed very efficiently through multiple thiol‐ene click reaction^[19]^ between a hexa‐*O*‐allyl α,α’‐trehalose derivative and Boc‐protected cysteamine.^[17]^ Coupling of building blocks **II** and **III**, followed by acid‐promoted hydrolysis of the carbamate groups in the macrocyclic adducts, afforded the target tri‐faceted cationic amphiphiles **1–3** in 44–62 % yield (Scheme 1; see the Supporting Information for details). Noteworthy, chromatographic purification could be efficiently accomplished on the Boc‐ended adducts. The subsequent deprotection step was a quantitative process as confirmed by the disappearance of the *tert*‐butyl proton signal in the corresponding ^1^H NMR spectra. The final compounds were isolated as perhydrochloride salts upon two‐fold lyophilisation from 0.1 M HCl solutions.

The reaction of isothiocyanates with amines to produce thioureas is highly efficient without need of catalyst and insensitive to oxygen and moisture, meeting all the criteria of click chemistry, and is particularly well‐suited for multiconjugation schemes.^[20]^ We have further used it for the post‐modification of the cationic heads in **1–3** by addition of the isothiocyanate‐armed branching element **11** ^[21]^ and subsequent carbamate hydrolysis. The newly generated thiourea functionalities in the resulting MM‐MNPs **4–6** are then expected to contribute to nucleic acid binding through cooperative hydrogen bonding interactions.^[22]^ In addition to twelve primary amines, the adducts further incorporate six tertiary amine centers per cationic patch in the branching points, which will habilitate the so‐called proton sponge mechanism for endosomal escape by imparting buffering capabilities.^[23]^ An analogous reaction sequence implying the cysteaminyl‐equipped tetrasaccharide diisothiocyanate (structure **IV**) and hexa‐*O*‐alkylated (C_6_ or C_14_) disaccharide diamines (structure **V**) provided the reverse C_2_L_1_ MM‐MNPs **7** and **8**, which upon homologation by reaction with **11** delivered the dendronized adducts **9** and **10** (Scheme 1; see the Supporting Information for details).

The structure and homogeneity of all MM‐MNPs, as the corresponding perhydrochloride salts, were gauged by ^1^H and ^13^C NMR spectroscopy (Figures S1‐S28, Supporting Information), mass spectrometry (MS) and elemental analysis. It is worth noting that the new vectors have a single *C_2_* axis of symmetry, meaning that the two α‐glucopyranoside constituents of the α,α’‐trehalose moieties in the twin lipid or cationic patches of C_1_L_2_ (**1**‐**6**) or C_2_L_1_ (**7**‐**10**) CT3 derivatives are not magnetically equivalent. This translates into three different set of signals for the carbohydrate subunits in the NMR spectra. Electrospray ionization (ESI)‐MS confirmed the expected molecular masses for the Mickey Mouse amphiphiles that keep the cysteaminyl groups underivatized (**1**–**3** and **7**, **8**; Figures S29–S31, S35 and S36, Supporting Information). The corresponding pseudomolecular ions of the higher dendroidal homologues (**4**–**6** and **9**, **10**) were not clearly observed under the same conditions because their numerous thiourea and amine groups prevented them from ionizing. For those cases we implemented an alternative strategy, consisting in the preformation of noncovalent complexes with a single‐stranded 12‐mer DNA (5‐AAGCCCGCCCAA‐3; DNAi).^[24]^ Since the compounds are conceived to co‐assembly with DNA, we advanced that an efficient association would take place, partly masking their strong polycationic character. We were delighted to see that the resulting MM–MNP/DNAi complexes could be subjected to matrix‐assisted laser desorption ionization time‐of‐flight (MALDI‐TOF) MS, providing signals that matched very well the theoretical masses (Figures S32–S34, S37 and S38, Supporting Information).

### Self‐assembling and pDNA co‐assembling properties

**Figure 3:**
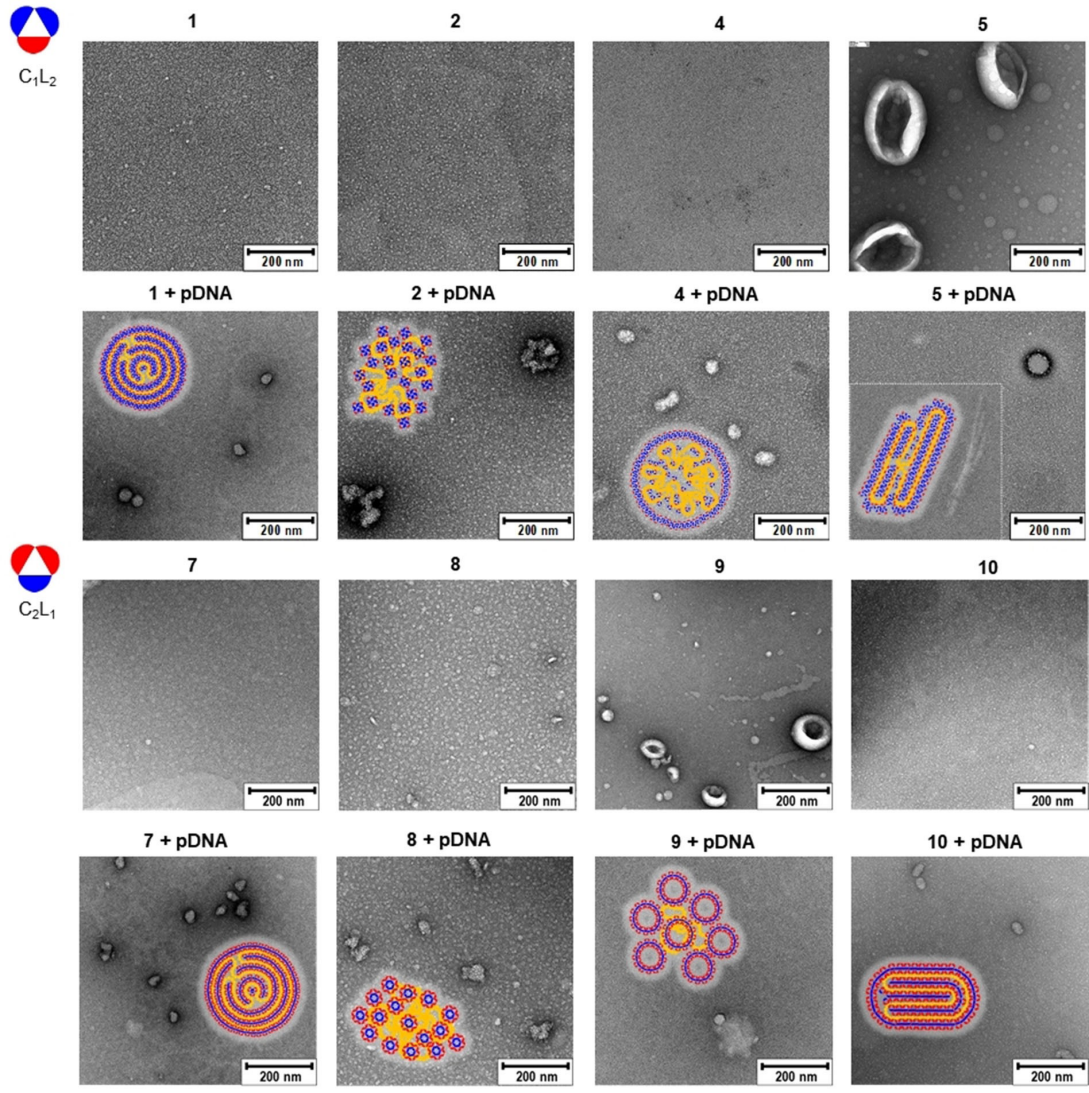
Representative TEM images recorded on the C_1_L_2_ (**1**, **2**, **4** and **5**) or C_2_L_1_‐type (**7**–**10**) Mickey Mouse molecular nanoparticles as well as on the corresponding CTplexes formulated with pDNA (luciferase‐encoding reporter gene pCMV‐Luc VR1216) at N/P 20 (see also Figure S39, Supporting Information, for selected high magnification images). Schematic representations of the proposed arrangements of the molecular nanoparticle constituents in the spherical multilamellar (**1**/pDNA and **7**/pDNA), morula (**2**/pDNA and **8**/pDNA), core‐shell (**4**/pDNA) filamentous (**5**/pDNA; inset), globular (**9**/pDNA) or cylindrical superstructures (**10**/pDNA) are also depicted. The corresponding TEM micrographs for the C_1_L_2_ MM–MNP vectors **3** and **6** and for the respective **3**/pDNA and **6**/pDNA formulations are very similar to those obtained from **1** and **4** and are not shown.

Dynamic light scattering (DLS) and mixed mode measurement phase analysis light scattering (M3‐PALS) for the hexanoylated or *n*‐hexylated C_1_L_2_ MM‐MNPs **1**, **4** and **3**, **6**, respectively, provided poor quality data indicative of insufficient particle counts at the concentrations used for pDNA complexation, which reflects their inability to form stable assemblies. Transmission electron microscopy (TEM, Figure 3) revealed the presence of low densities of small aggregates (<10 nm diameter), probably arising from transient clusters of a few molecules that are captured upon drying the sample on the TEM grid. Such clusters become more stable for the hexacysteaminyl‐dodecamyristoylated derivative **2**, as observed both by DLS (average hydrodynamic diameter, D_h_, 15 nm; ζ‐potential 38 mV) and TEM, likely due to its decreased water solubility. Differently, the dendronized homologue **5** self‐assembled into giant vesicles (D_h_ 320 nm; ζ‐potential 41 mV). In the C_2_L_1_ cyclohexasaccharide series, compounds **7** and **10** did not aggregate, whereas **8** afforded spherical and cyclindrical micelles (D_h_ 12 nm; ζ‐potential 35 mV) and **9** was found by TEM to form vesicles of 30–150 nm (Figure 3).

The possibility to bias the preference to integrate high‐ or low‐curvature self‐assembled constructions by molding the MM–MNP vector architecture was anticipated to further impact the topology and stability of their co‐assemblies with nucleic acids. To test this prediction, the TEM images of the supramolecular complexes prepared with pDNA (luciferase‐encoding pCMV‐LucVR1216) and the MM‐MNPs at protonable nitrogen/phosphorus (N/P) ratio 20 in 4‐(2‐hydroxyethyl)‐1‐piperazineethanesulfonic acid (HEPES) buffer (pH 7.4, 10 mM) were recorded. The micrographs obtained from **2**/pDNA formulations showed irregular morula‐like aggregates of variable shape and size (150–300 nm), likely resulting from electrostatically‐driven disordered co‐aggregation between clusters of **2** and pDNA, whereas in the case of **5**/pDNA CTplexes globular (100–150 nm) and filamentous objects (>200 nm) coexisted. The first can arise from the direct interaction of the plasmid with self‐assembled vesicles of the vector. Filamentous and worm‐like topologies on their side are typically observed for macromolecular polycations with a marked preference for linear over curved arrangements upon pDNA templation.^[25]^ These types of complexes have been shown previously to be inadequate for gene delivery applications due to inefficient protection of the nucleic acid cargo^[26]^ and were not further pursued. The other C_1_L_2_ MM‐MNPs **1**, **3**, **4** and **6** afforded quasi‐spherical CTplexes of 30–50 nm diameter upon co‐assembling with pDNA. The **1**/pDNA and **3**/pDNA nanocomplexes showed a sinusoidal, lamellar ultrathin structure characteristic of alternated DNA segments (dark; high electron density) and amphiphile bilayers (light; low electron density).^[27]^ This co‐organization mode is usually encountered in complexes of DNA and polycationic amphiphilic cyclodextrins, being generally associated to high transfection efficacy.^[28]^ In the case of **4**/pDNA and **6**/pDNA CTplexes such snake‐like arrangement is missing. Instead, the particles appear as bright white objects in the TEM micrographs, strongly suggesting a core‐shell organization evocative of enveloped viruses:^[29]^ after an initial condensation phase promoted by electrostatic interactions, the negatively charged “dark” core thus formed is encircled by an external “light” shell of the surfactant (Figure 3; see also Figure S39, Supporting Information). Analogous nanostructures have been previously assembled by sophisticated multiformulation strategies^[30]^ and, more recently, from α,α’‐trehalose‐based Siamese‐twin amphiphiles,^[17]^ and found to be appropriate for nucleic acid cargo delivery to target cells and specific organs.

In the C_2_L_1_ MM–MNP series, compound **7** (*n*‐hexylated) likewise formed spherical aggregates with a multilamellar ultrathin structure upon co‐assembly with pDNA. The TEM images of the CTplexes formulated with **8** (*n*‐tetradecylated) showed morula‐like particles, suggesting that nanocomplex formation involved self‐assembled micelles and not the individual vector molecules. The influence of the hydrophobic/hydrophilic balance in determining the preference for a lamellar or a morula‐like arrangement has been previously noticed, the later architecture being mostly detrimental for transfection.[^17^, ^28a^] Compound **9** (the dendronized analogue of **7**) formed instead globular particles pDNA, likely involving the co‐aggregation of self‐assembled vesicle of the vector with pDNA. This scenario is similar to that observed for **2**/pDNA and **5**/pDNA CTplexes, but particles are now smaller (≈50 and 100 nm, respectively) and, a priori, better suited for transfection studies. Finally, compound **10** (the dendronized analogue of **8**) was unique within the whole set of MM‐MNPs synthesized in providing small cylindrical nanoparticles upon co‐assembling with pDNA (Figure 3; see also Figure S39, Supporting Information).

DLS of colloidal MM–MNP/pDNA nanocomplex solutions at N/P ratios 5, 10, and 20 in HEPES evidenced the formation of unimodal populations of particles for most formulations, with hydrodynamic diameters comprised between 65 and 150 nm and polydispersity index values <0.3. The exceptions were the C_1_L_2_ CT3 myristoylated derivatives **2** (at all studied N/P values) and **5** (at N/P 5 and 10). It was found that the size of the CTplexes decreased monotonically as the proportion of the cationic vector increased, whereas the ζ‐potential followed the opposite trend, reaching positive values in the range 23–36 mV at N/P 20. Of note, compounds **4** and **6** originated negatively charged nanoparticles upon co‐assembling with pDNA at N/P 5, supporting the proposed two‐phase (nucleation/coating) mechanism leading to core‐shell nanoparticles (Table 1).

**Table 1:** Hydrodynamic diameter (D_h_) and ζ‐potential of the nanocomplexes formulated with the Mickey Mouse molecular nanoparticles **1**–**10** and pDNA (luciferase‐encoding pCMV‐LucVR1216) at protonable nitrogen/phosphorus (N/P) ratios 5, 10, and 20 in 4‐(2‐hydroxyethyl)‐1‐piperazineethanesulfonic acid (HEPES) buffer (pH 7.4, 10 mM).^[a]^

N/P	5		10		20	
	D_h_ (nm)	ζ (mV)	D_h_ (nm)	ζ (mV)	D_h_ (nm)	ζ (mV)
**1**	145±2	16±2	147±2	25±1	102±3	31±2
**2**	>500	–	>500	–	>500	–
**3**	148±25	6±4	108±11	18±8	86±1	36±14
**4**	230±3	−25±1	177±3	16±2	123±4	33±2
**5**	>500	–	>500	–	157±6	35±2
**6**	108±20	−9±5	74±2	25±9	66±2	27±12
**7**	108±6	21±1	93±1	23±2	82±1	29±2
**8**	104±11	28±1	88±2	33±1	86±2	33±3
**9**	138±11	28±17	106±28	23±6	93±4	28±8
**10**	151±21	9±5	80±10	21±9	65±1	23±2

[a] Except for formulations based in compound **2** (all N/P values) and **5** (N/P 5 and 10), polydispersity index values were <0.3 in all cases.

**Figure 4:**
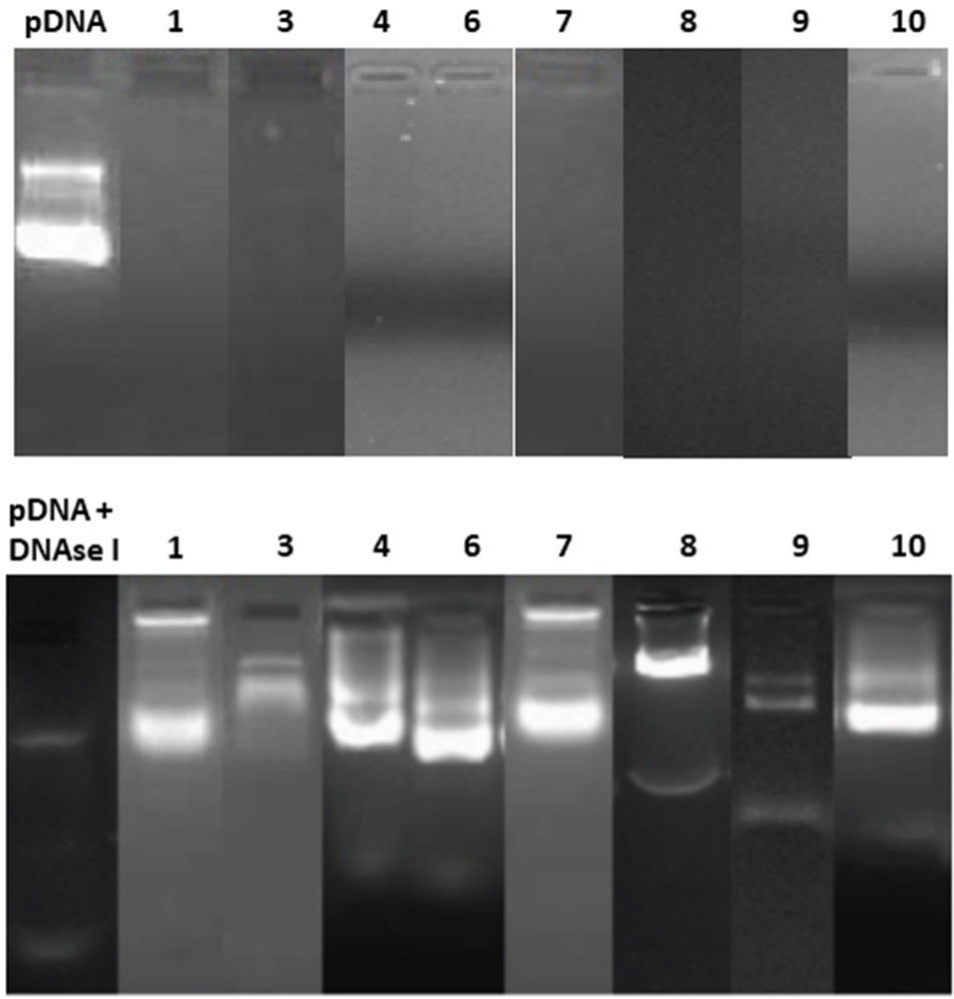
EMSA gels for CTplexes formulated with compounds **1**, **3**, **4** and **6**–**10** at N/P 10, before (upper panel) and after treatment with DNAse I and subsequent dissociation of the complexes with SDS (lower panel). Naked pDNA and ethidium bromide were used as the control and staining reagent, respectively. Notably, pDNA complexation results in arrested migration to the anode, whereas degradation leads to the disappearance of any visible band due to disruption of the double helix, which prevents intercalation of the fluorescent probe.

The above MM–MNP/pDNA formulations were next analyzed by electrophoresis mobility shift assay (EMSA) in 0.8 % agarose gel, with staining by the intercalating agent ethidium bromide, for assessing DNA complex formation and protection as well as DNA integrity (Figure 4; note that the lanes of interest, coming from different EMSA experiments conducted under identical conditions, are shown. The corresponding gel pictures from which these lanes were taken, with the individual references, are collected in Figure S40, Supporting Information). Except for **2** and **5** (data not shown), full pDNA complexation and protection were achieved in all cases, as inferred from the capacity of the compounds to arrest migration of pDNA in the gel and the recovery of the essentially unaltered pDNA after DNase I/sodium dodecyl sulfate (SDS) treatment. These results, together with the rather small size and homogeneity of the CTplexes, make them promising candidates for the development of systemic applications in vivo by limiting size‐restricted diffusion and epithelial permeation and absorption.

### Computational assessment of CTplex assembly

The above DLS and TEM data illustrate the potential of shifting between the C_1_L_2_ and the C_2_L_1_ Mickey Mouse CT3 prototypes to fine‐tune the topology of the vector/pDNA co‐aggregates. The exception is the pair **1** (or **3**) versus **7**, combining unbranched cysteaminylpropyl cationic arms and C_6_ lipophilic tails: quasi‐spherical multilamellar nanocomplexes with similar ζ‐potential, securing pDNA protection, were observed in both series, in spite of the expected substantial change in the hydrophilic/hydrophobic balance. Preliminary circular dichroism studies were consistent with neither **1** nor **7** self‐associating in HEPES at the concentrations used for CTplex formulation and both being able to intermingle with DNA as individual molecules (Figures. S46–S48, Supporting Information). To get a deeper insight on the interactions at play, the stability of antiparallel cyclohexasaccharide dimers facing their hydrophobic domains, both in the bulk and in the confined space between DNA fragments, was investigated by molecular mechanics (MM) and molecular dynamics (MD) simulations conducted in explicit water. Such dimers can be considered as the basic elements of vector bilayers.^[31]^

**Figure 5:**
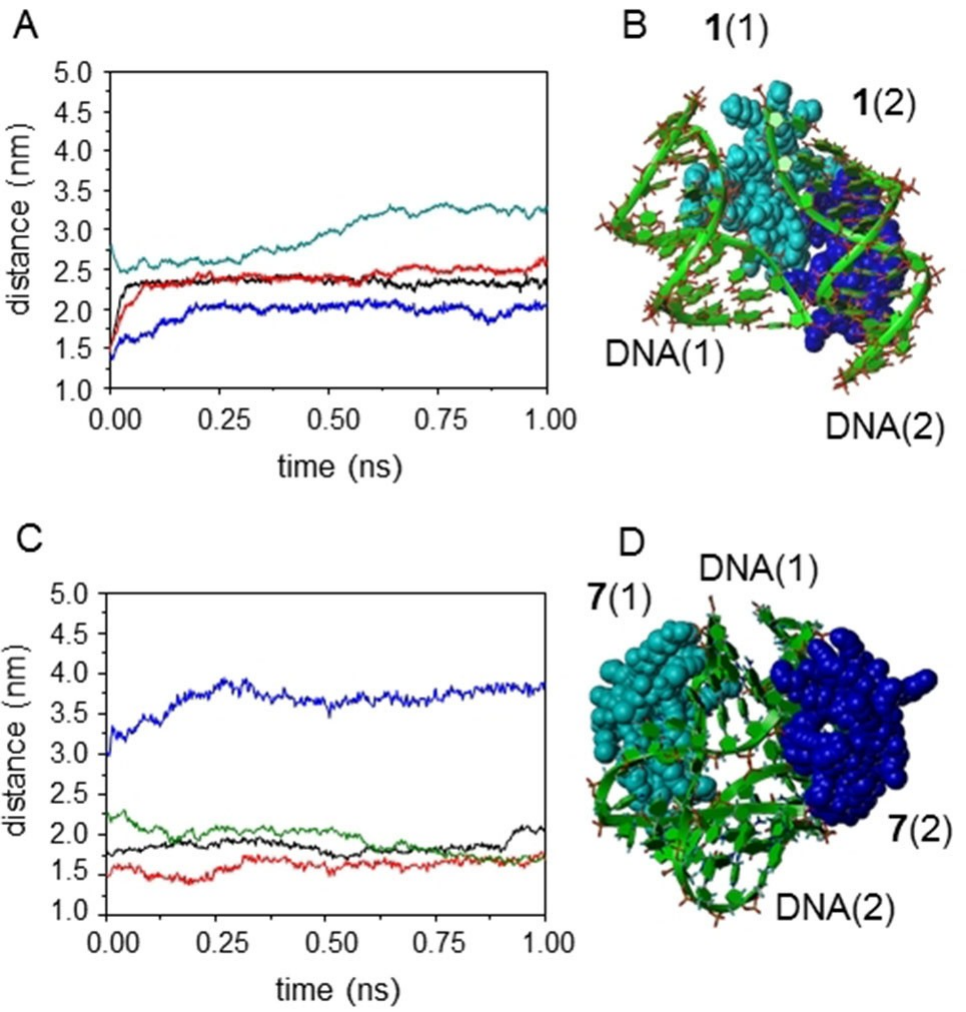
A) MD histories for the distances DNA(1)‐**1**(1) (black), DNA(2)‐**1**(2) (red), **1**(1)‐**1**(2) (blue) and DNA(1)‐DNA(2) (green). B) Structure of the (**1**‐dimer)(DNA)_2_ nanocomplex averaged throughout the 1 ns MD trajectory. C) MD histories for the distances DNA(1)‐**7**(1) (black), DNA(2)‐**7**(2) (red), **7**(1)‐**7**(2) (blue) and DNA(1)‐DNA(2) (green). D) Structure of the (**7**‐dimer)(DNA)_2_ nanocomplex averaged throughout the 1 ns MD trajectory (DNA fragments in green; the individual vector monomer constituents **1** or **7** are colored in light and deep blue).

Initially, the minima binding energy (MBE) structures for **1** or **7** perhydrochloride dimers were optimized (MM), then devoid from the chlorine atoms (net charge of each patchy CT3 derivative +6 and +12 esu for **1** and **7**, respectively) and used as the starting conformations for the computation in water of (MM–MNP)_2_/(DNA)_2_ complexes (Figures S41–S44, Supporting Information). Subsequent MD simulations showed that the charged cyclohexasaccharide dimersreadily dissociated in the absence of the flanking DNA fragments. Reciprocally, the DNA chains fall apart in the absence of the interposed cationic vectors. However, the supramolecular (MM–MNP)_2_/(DNA)_2_ complexes remained stable during 1 ns MD simulations in the presence of water. Some differences arose, however, between the complex built from the C_1_L_2_ derivative **1** and the C_2_L_1_ counterpart **7**: whereas in the first case the distance between the constitutive vector monomers remained stable throughout the whole MD trajectory (Figure 5A and B), in the second such distance became significantly larger (Figure 5C and D). Conceivably, the presence of a single lipophilic patch in **7** results in weaker van der Waals interactions between the vector molecules as compared with **1**, which is compensated because the cationic patches in **7** can bridge the DNA segments in the complex. Such differences are expected to lead to variances in external/internal charge as well as in the degradability of the corresponding CTplexes, which are factors that greatly influence the in vivo tropism.^[32]^ Both **1** and **7** were thus retained for the comparative assessment of their transfection capabilities in vitro and in vivo.

### Toxicity and in vitro cell transfection

**Figure 6:**
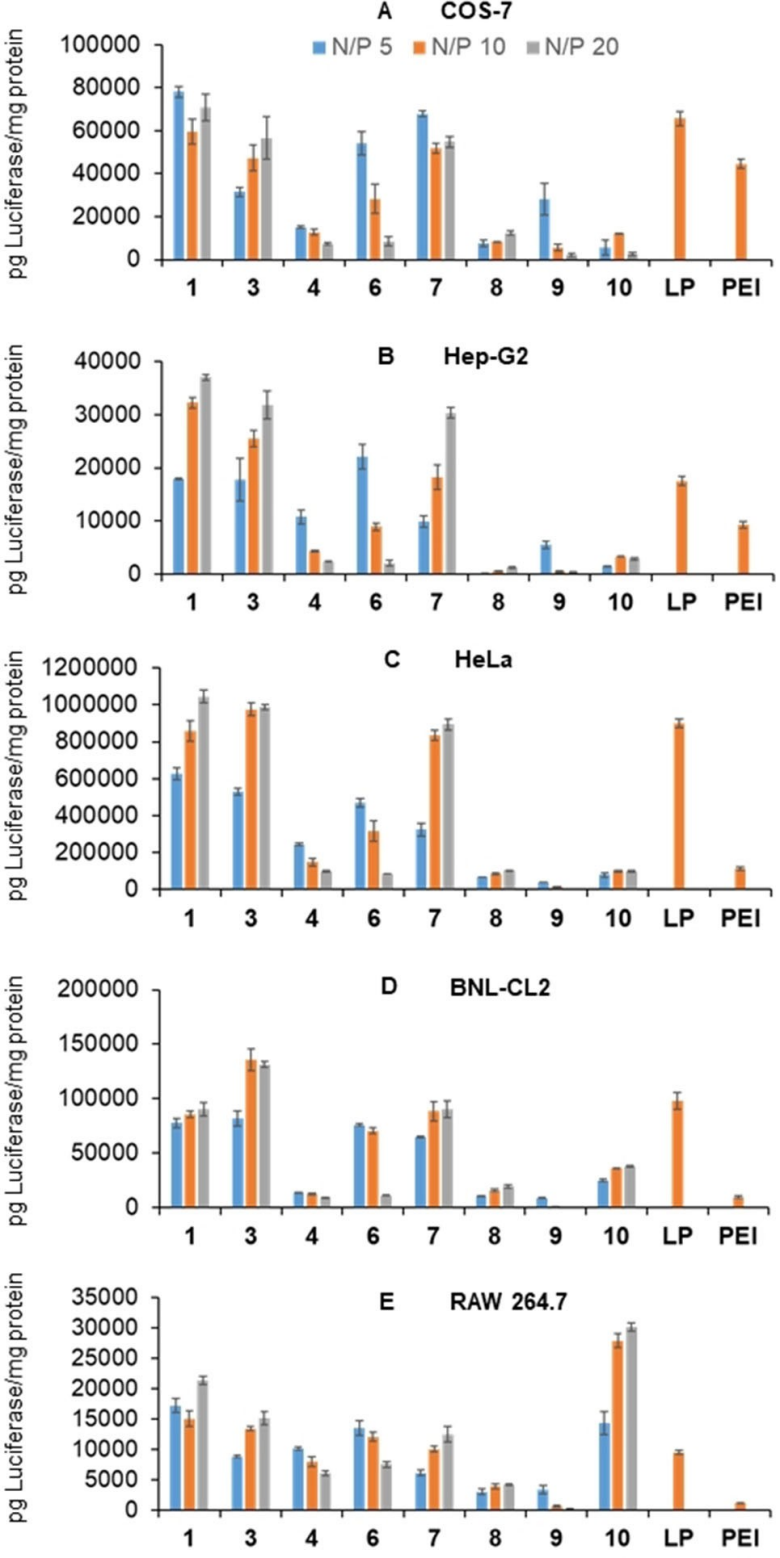
Luciferase expression in (A) COS‐7, (B) HepG2, (C) HeLa, (D) BNL‐CL2, and (E) RAW 264.7 cells promoted by CTplexes formulated with the MM‐MNPs **1**, **3**, **4** or **6**–**10** and the luciferase‐encoding reporter gene pCMV‐Luc VR1216 at N/P 5, 10, and 20 in the presence of fetal bovine serum (FBS, 10 %). Data obtained with Lipofectamine 3000® polyplexes and bPEI polyplexes (N/P 10) under identical conditions are included for comparative purposes. The data represent the mean ± standard deviation (SD) values of three wells and are representative of three independent determinations.

CTplexes formulated from the CT3‐scaffolded Mickey Mouse molecular nanoparticles **1**, **3**, **4**, **6**–**10** and the luciferase‐encoding reporter gene pCMV‐Luc VR1216 at N/P 5, 10, and 20 were next assayed for their transfection capabilities in vitro in African green monkey epithelial kidney COS‐7, human hepatocellular carcinoma HepG2, human cervical carcinoma HeLa, murine embryonic hepatocyte BNL‐CL2, and murine macrophage RAW 264.7 cells (Figure 6). The latter linage is known to be notably more recalcitrant to transfection.^[33]^ Polyplexes generated frombranched polyethylenimine (bPEI, MW=25 kDa; N/P 10), the gold standard cationic polymer for nonviral gene delivery,^[34]^ and polyplexes formulated from Lipofectamine 3000® (LP, a commercial cationic lipid formulation) were also included in our screening as controls for comparative purposes. All experiments were conducted in the presence of 10 % serum, the optimal conditions for the controls.

Patchy CT3 derivatives giving rise to spherical particles with a well‐defined lamellar organization, namely **1** and **3** in the C_1_L_2_ series and **7** in the C_2_L_1_ series, outperformed bPEI at all N/P values and rivalled Lipofectamine 3000® performance in all cell lines. Nanocomplexes with an analogous topology obtained from polycationic amphiphilic cyclodextrins were previously found to enter the cell preferentially through a caveolae‐mediated endocytic pathway that favored their rapid accumulation at the vicinity of the nucleus and led to high transfection levels.^[35]^ The evident analogies suggest that a similar mechanism might operate here. Excepting in COS‐7 cells (Figure 6A), compounds **4** and **6**, affording core‐shell nanoparticles upon co‐assembly with pDNA, also overpassed bPEI polyplexes in these experiments. However, the transfection efficiency monotonically decreased with the N/P value, suggesting that formation of the external compact shell results in very stable CTplexes from which release of the pDNA cargo intracellularly takes place at a lower rate. The situation is analogous to that encountered for CTplexes obtained from dendronized CT3‐centred star polymers.^[12]^ The irregular morula and globular shaped CTplexes formulated from **8** and **9** showed comparatively poorer transfection efficiencies. Indeed, nanocomplexes obtained from pDNA and molecular vectors forming stable micelles typically have a higher tendency to aggregate and poorer transfection capabilities^.[17,28a,36]^ Remarkably, the cylindrical **10**/pDNA CTplexes displayed very high selectivity towards the macrophage cell line RAW 264.7, reaching expressions of the encoded luciferase protein over one order of magnitude higher than bPEI polyplexes, about 3‐fold as compared with LP lipoplexes (Figure 6E). This is consistent with reports showing that macrophages internalize ellipsoidal particles much faster than spherical particles.^[37]^ Altogether, the results demonstrate that the size, nature and relative distribution of the cationic and lipophilic patches in the cyclohexasaccharide core exerts a noteworthy influence on the cell selectivity of the resulting MM–MNP based nanocomplexes, highlighting the interest of strategies compatible with macromolecular tailoring for specific applications. Most notably, several of the new vectors performed better than bPEI and Lipofectamine 3000® for all the cell lines, even at the lowest N/P 5 ratio, while showing no toxicity in the whole range of N/P ratios and cell linages screened (Figure S45, Supporting Information).

### In vivo transfection

**Figure 7:**
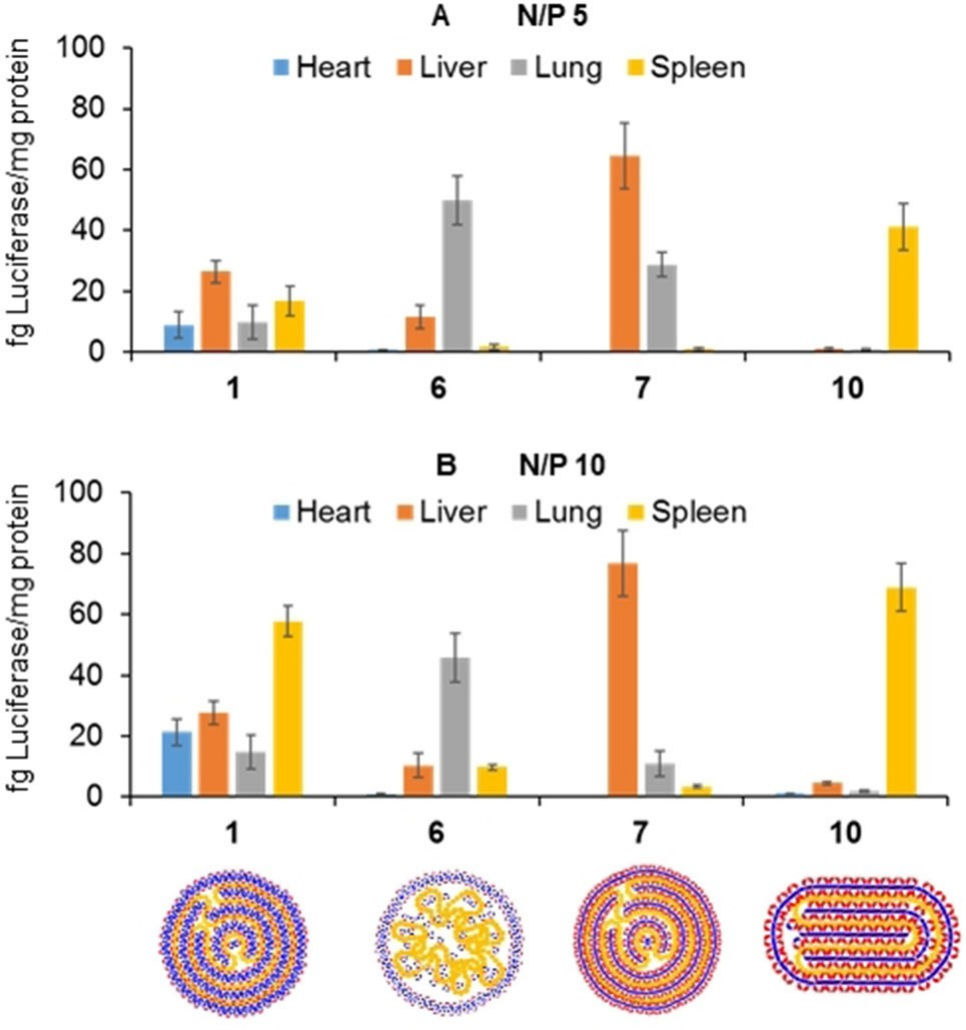
Gene expression conducted in vivo after intravenous administration of 60 μg of pCMV‐Luc VR1216 formulated with the MM‐MNPs **1**, **6**, **7** and **10** at (A) N/P 5 or (B) N/P 10. Bars represent the mean±SD (n=8 animals). Schematic representations of the nanocomplex shape and internal structure (spherical multilamellar for **1** and **7**, core‐shell for **6** and cylindrical for **10**) as also depicted

The shape and size of nanoparticles are known to considerably affect their biodistribution and option of endocytic pathways and have been found to be main determinants regarding the in vivo tropism of molecular vector‐based pDNA nanocomplexes.^[38]^ The possibility to program different topologies at the nanoscale by tailoring the vector architecture at the molecular level, enabled by the Mickey Mouse CT3 prototype, offers exciting prospects in this sense. To explore this concept, CTplexes formulated from pDNA (pCMV‐Luc VR1216) and the C_1_L_2_ type MM‐MNPs **1** and **6** or the C_2_L_1_ members **7** and **10** at N/P 5 and 10 were systemically injected into mice, and their activity in the liver, heart, lungs, and spleen was compared with PBS and the naked DNA as negative controls. All animals were studied in accordance with guidelines established by Directive 86/609/EEC and with the approval of the Committee on Animal Research at the University of Navarra (accreditation number CEEA 017‐19). The results, based on the luciferase reporter gene expression, indicated that 24 h after the intravenous administration of N/P 5 **1**/pDNA CTplexes, which formed spherical multilamellar particles, similar transfection levels were reached at all the analyzed organs. At N/P 10 transfection occurred preferentially at the spleen, but it was still significant at the heart, liver and lung. Sharply differently, the core‐shell **6**/pDNA nanocomplexes exhibited a marked tropism to the lung that was slightly higher at N/P 5 than at N/P 10. Compound **7**, which similarly to **1** afforded quasi‐spherical multilamellar CTplexes, mediated instead transfection in the liver with high selectivity, especially at N/P 10. This is ascribable to differences in the internal structure as suggested by the computational studies above discussed. In further support of this hypothesis, in vivo results obtained for N/P 10 CTplexes of comparable shape and size formulated with compound **3** portrayed an organ transfection profile closely matching that of **1** (less than 10 % difference in normalized luciferase expression values in heart, liver, lung and spleen; data not shown), with which **3** shares the same C_2_L_1_ architecture. Finally, the cyclindrical CTplexes formulated from **10** almost exclusively transfected the spleen at either N/P ratio, with negligible luminescence detected in other organs (Figure 7).

The fact that the MM–MNP vectors allow simultaneous transfection of multiple internal organs or selective delivery of the nucleic acid cargo to a specific organ is remarkable. Even more notable is that the organ destination can be switched between lung, liver or spleen using the same CT3 platform by appropriately designing the different patches on the CT3 core, with no need of a targeting ligand. Differential mRNA delivery to the liver or the spleen has been previously achieved with lipid nanoparticles (LNPs) by adjusting the N/P ratio, thus the charge of the lipoplexes.^[39]^ Very recently, Siegwart and co‐workers reported that a variety of LNPs can be engineered to exclusively deliver mRNA to extrahepatic tissues via addition of supplemental molecules that allow tuning the internal charge of the lipoplexes, so‐called selective organ targeting (SORT) molecules.^[40]^ Lung‐, spleen‐ and liver‐targeted SORT LNPs were designed in this manner. In our case, tuning the topological landscape of the nanocomplexes let achieve similar organ‐selective transfection profiles, with the notable difference that the MM‐MNPs here reported are single molecule, perfectly monodispersed species effective in monoformulation.

## Conclusions

The ensemble of data provides conclusive evidence on the strong potential of judiciously installing different arrangements of functional elements onto a cyclotrehalan core to efficiently access perfectly monodisperse patchy molecular nanoparticles with tailored structures and properties beyond the Janus archetype. The Mickey Mouse materials here reported were conceived to condense pDNA into nanocomplexes through concurrent electrostatic and hydrophobic interactions. The data spotlight the advantage of macromolecular diversity‐oriented strategies compatible with strict control over the structural parameters, including the nature and valency of the patches, to modulate the self‐ and co‐assembling behaviors. The remarkable cell selectivity and, especially, organ selectivity differences observed within the series of MM‐MNPs prepared emphasize the compelling effect of both the vector architecture and the nanocomplex topology on the biological activity. Taken together, these results inform a versatile prototype for the construction of fully synthetic anisotropic nonviral gene delivery systems uniquely suited for optimization schemes.

## Experimental Section

Experimental details are given in the Supporting Information.

## Conflict of interest

The authors declare no conflict of interest.

## Supporting information

As a service to our authors and readers, this journal provides supporting information supplied by the authors. Such materials are peer reviewed and may be re‐organized for online delivery, but are not copy‐edited or typeset. Technical support issues arising from supporting information (other than missing files) should be addressed to the authors.

Supplementary
