# Supplementary material for: Trifaceted Mickey Mouse Amphiphiles for Programmable Self‐Assembly, DNA Complexation and Organ‐Selective Gene Delivery
Source: Chemistry. 2021 May 26;27(36):9429–38. doi: 10.1002/chem.202100832 (PMC8361672; doi:10.1002/chem.202100832)
Supplement: Supplementary file 1 — Supplementary [file CHEM-27-9429-s001.pdf]

Chemistry–A European Journal

Supporting Information

Trifaceted Mickey Mouse Amphiphiles for Programmable Self-Assembly, DNA Complexation and Organ-Selective Gene Delivery

Ana I. Carbajo-Gordillo[+], Manuel González-Cuesta[+], José L. Jiménez Blanco, Juan M. Benito, María L. Santana-Armas, Thais Carmona, Christophe Di Giorgio, Cédric Przybylski, Carmen Ortiz Mellet,* Conchita Tros de Ilarduya,* Francisco Mendicuti,* and José M. García Fernández*

LIST OF CONTENTS

1. Materials, general methods, charaterization techniques, cell culture
and cell and in vivo assay specifications…………………………………………..S1
1. Synthesis, Schemes S-S6..S6
4. ^{1}H and ^{13}C NMR spectra of the new compounds, Figs. S1-S28………………......S27
5. Mass Spectra, Figures S29-S38…………………………………………………S55
6. High magnification TEM images of representative nanocomplexes formulated
with pDNA and the MM-MNPs **1**, **4**, **7** and **8**, Figure S39...S58
7. Complete EMSA gels with individual references, Figure S40................................S59
7. Detailed protocols for the computational assessment of CTplex assembly
from the C_1L_2 or C_2L_1 MM-MNPs **1** or **7** and DNA, Figures. S41-S44……………...S60
8. Cell viability (MTT assay) for the C_1L_2 MM-NPs **1**, **3**, **5** and **7** and for
the C_2L_1 MM-MNPs **7-10**, Figure S45.……………………………..……..……S64
9. UV-Vis and circular dichroism spectra of the MM-MNPs **1** and **7** and their
complexes with ctDNA, Figures 46-48……………………………………..…..S65
References………………………………………………………………….……...S67

Materials

Reagents and solvents, unless otherwise indicated, were purchased from commercial sources and used without further purification. For MALDI-TOF mass spectrometry experiments single strand oligodeoxyribonucleotide (DNAi), with the following sequence 5’-AAGCCCGCCCAA-3’ (M_W: 3584.4 g·mol^{-1}) was purchased from Eurogentec (Seraing, Belgium). Polyethyleneimine 25 (bPEI, MW 25 kDa, branched) was purchased from Aldrich. The plasmid (luciferase-encoding plasmid pCMV-Luc VR1216, 6934 base pairs) was purchased from Clontech (Palo Alto, CA, USA). The following materials were used for DNAse I protection assays: agarose D-1 (Pronadisa, Madrid, Spain), Tris-boric acid-EDTA Buffer (10x TBE buffer) (Invitrogen, Barcelona, Spain), DNAse I and ethidium bromide (Gibco BRL, Barcelona, Spain). Sodium dodecyl sulphate (SDS) and NaCl (RoigFarma, Barcelona, Spain) were used to release DNA from the complexes. Ethylenediaminetetraacetic acid (EDTA) and DMSO Hibry-Max® were supplied from Sigma. AlamarBlue® dye was purchased from Accumed International Companies (Westlake, OH, USA). The synthesis of all cyclotrehalan-based MM-MNPs included in this study, as well as the structural characterization data for all of them and the corresponding synthetic intermediates, is described in the Supporting Information.

General methods

^{1}H (^{13}C) NMR spectra were recorded at 600 (150.9), 500 (125.7) and 400 (100.6) MHz. 2D COSY, 1D TOCSY, and HMQC experiments were used to assist assignments (see the Supporting Information). Thin-layer chromatography (TLC) was carried out on aluminium sheets coated with Kieselgel 60 F245 (E. Merck), with visualization by UV light and by charring with 10% H_2SO_4 or 0.1% ninhydrin in EtOH. Column chromatography was carried out on silica gel 60 (E. Merck, 230-400 mesh). Electrospray mass spectrometry (ESI-MS) was run in a Bruker Esquire6000 instrument. Elemental analyses were performed at the Institute for Chemical Research (IIQ, CSIC – Univ. Sevilla, Spain).

MALDI-TOF experiments by preformation of MM-MNP/ssDNA complexes.

Samples for these experiments were prepared as follows: a stock solution of ssDNA (5’-AAGCCCGCCCAA-3’; DNAi) was prepared at 100 μM in aqueous ammonium acetate (100 mM). Then, the solution was further diluted to 20 μM in 100 mM ammonium acetate. Several mixtures involving various N/P ratios, from 1 to 30, were tested. Matrix solutions of DHAP or DHB at 100 mM, mixed with ammonium citrate at 10 mM, were prepared in 50/50 (v/v) water/methanol. Just before MS analysis, equal volumes of matrix solution and sample solutions at different N/P values were mixed, and let during 15 min at 37 °C. Then, the mixture was spotted onto the MALDI MTP 384 polished steel plate by the dried droplet method and allowed to dry at

room temperature during 15 min before analysis. Subsequent MALDI-TOF MS experiments were performed using an Autoflex III MALDITOF/TOF spectrometer (Bruker Daltonics, Bremen, Germany). This instrument was equipped with a Nd:YAG SmartBeam laser (λ= 355 nm) pulsed at a 200 Hz frequency. The mass spectrometer was operated in the linear mode with an accelerating potential of 19 kV. The extraction delay was optimised from 0 to 300 ns. Mass spectra were obtained by accumulation of 1000 laser shots and processed using Flex Analysis 3.0 software (Bruker Daltonics). The instrument was calibrated using standard peptide and protein mixtures provided by the manufacturer. For samples, both positive and negative ionization mode were investigated, but only spectra acquired in positive polarity have given exploitable signal.

Preparation of CTplexes

The quantity of cyclooligosacharide used in each formulation was calculated according to the desired DNA concentration, the N/P ratio, the molecular weight and the number of protonable nitrogen atoms in the corresponding cationic derivative. Typically, pDNA was diluted in HEPES (10 mM, pH 7.4) to the desired final concentration as specified hereinafter, and then the desired amount of CD derivative was dispersed in this solution from a stock solution in DMSO (typically 1-10 mM). The resulting mixture (with a final DMSO content below 1% in all cases) was instantly vortexed thoroughly and the complexes were incubated for 1 h prior to subjecting them to characterization or transfection experiments.

Electrophoresis mobility shift assay

Each MM-MNP:pDNA complex (20 µL, 0.4 µg of plasmid or ctDNA) was submitted to electrophoresis for about 30 min under 150 V through a 0.8% agarose gel in TAE 1× (Tris-acetate-EDTA) buffer and stained by spreading ethidium bromide. The pDNA was then visualized after photographing on an Alphaimager Mini UV transilluminator. The plasmid integrity in each sample was confirmed by electrophoresis after decomplexation with sodium dodecyl sulfate (SDS, 8%).

DNA condensation/protection assays

50 µL of CTplexes were prepared in water at different N/P ratios to a final concentration of 50 µg/mL. Then, samples were electrophoresed for 30 min under 150 mV in 0.8% agarose gel. For protection assays, DNAse I (1 U/µg pDNA) was added to each sample and stirred for 30 min at 37 ºC. 20 µL of 0.25 M EDTA was added to inactivate DNAase I and the sample was vortexed and incubated for 5 min. 20 µL of 25% SDS was then added and further incubated for 5 min. Samples were electrophoresed as described above. Plasmid integrity was compared with free pDNA both treated and untreated (Figure S40).

Particle size and ζ-potential measurements

The size of the CDplexes was measured by dynamic light scattering (DLS), and the overall charge (ζ-potential) by "Mixed Mode Measurement" phase analysis light scattering (M3-PALS) measurements using a Zetasizer Nano ZS (Malvern Instruments). All measurements were performed by triplicate. Size results are given as intensity distribution of the major population by the mean diameter with its standard deviation. ζ-potentials measurements on the MM-MNPs and CTplexes were made using Malvern Zetasizer Nano ZS instrument with "mixed-mode measurement" phase analysis light scattering (M3-PALS). The following specifications were applied: sampling time, automatic; number of measurements, 12 per sample; medium viscosity, 1.054 cP; medium dielectric constant, 80; temperature, 25 °C. Before each series of experiments, the performance of the instruments was checked with either a 90 nm monodisperse latex beads (Coulter) for DLS or with DTS 50 standard solution (Malvern) for ζ-potentials.

Transmission electron microscopy (TEM)

Formvar-carbon coated grids previously made hydrophilic by glow discharge were placed on top of small drops of the CTplexes (HEPES 20 mM, pH 7.4, DNA 303 μM phosphate) prepared as describe above using N/P 20 ratios. After 1-3 min, grids were negatively stained with a few drops of 1% aqueous solution of uranyl acetate. The grides were then dried and observed with a Philips CM12 electron microscope working under standard conditions. All these experiments were reproduced twice on each formulation.

Cell transfection efficiency

Cells (COS-7, HepG2, HeLa, BNL-CL2 or RAW 246.7) were seeded in medium in 48-well plates (Iwaki Microplate, Japan), and incubated for 24 h at 37 °C in 5% CO_2. The medium was removed and 0.3 mL of complete medium (activated FBS) and 0.2 mL of CTplexes (containing 1 µg of pDNA) were added to each well. After 4 h incubation the medium was replaced for complete medium and the cells were further incubated for 48 h. Cells were washed with phosphate-buffered saline (PBS) and lysed with 100 µL of Reporter Lysis Buffer (Promega, Madison, WI, USA) at room temperature for 10 min, followed by a freeze-thaw cycle. 20 µL of the supernatant was assayed for total luciferase activity using the luciferase assay reagent (Promega), according to the manufacturer's protocol. A luminometer (Sirius-2, Berthold Detection Systems, Innogenetics, Diagnóstica y Terapéutica, Barcelona, Spain) was used to measure luciferase activity. The protein content of the lysates was measured by de DC protein Assay Reagent (Bio-Rad, Hercules, CA, USA) using bovine serum albumin as the standard. The data were expressed as nano(pico)grams of luciferase (based on a standard curve for luciferase activity) per milligram of protein. Samples

were analyzed in a plate spectrophotometer Power Wave XS and a data processor KC junior, BioTek®.

Cell viability

For MTT assay $3{\cdot}10^3$ cells (COS-7, HepG2, HeLa, BNL-CL2 or RAW 264.7) were seeded in 96 well plates and incubated at 37 °C, in a humidified atmosphere containing 5% CO_2 till confluence. 50 μL per well of the selected dilution of the vector (or the controls PEI, Lipofectamine 3000®) formulations were then added. The plates were incubated for 24 hours at 37 °C and 5% CO_2. 10 μL per well of MTT (3-(4,5-dimethylthiazol-2-yl)-2,5-diphenyltetrazolium bromide) were then added at the final concentration of 5 mg/mL (MTT can be diluted in sterile PBS or in medium) and after an incubation of 6 h 100 μL per well of a lysant (10% SDS and 0.01 M HCl in H_2O) were added. Plates were sealed and incubated overnight at 37 °C and 5% CO_2. The absorbance of the color complex was read at 620 or 540 nm after shaking of the plates. The rate of toxicity/survival was determined respect to the corresponding cell line incubated in absence of the compound.

***In vivo* transfection activity**

Female Balb-c mice (6-8 weeks of age, 20-25 grams weigh) were purchased from Harlan Ibérica Laboratories. All animals were studied in accordance with guidelines established by Directive 86/609/EEC and with the approval of the Committee on Animal Research at the University of Navarra. Individual mice in groups of eight were injected via the tail vein with 200 μL of nanoplexes containing 60 μg of pCMV-Luc VR1216 plasmid DNA at N/P 5 and 10. Naked DNA was injected as control. Twenty-four hours after injection the mice were sacrificed. The liver, heart, lungs and spleen were collected and washed with cold PBS. The organs were homogenized with 1 mL lysis buffer using a homogenizer at 5000 rpm (Mini-Beadbeater; BioSpec Products, Inc., Bartlesville, OK, USA) and centrifuged at 10000 rpm for 3 min. 20 μL of the supernatant were analysed for luciferase activity following the same procedure as for in vitro assays.

Statistical analysis

Statistical analyses were performed using SPSS software from SPSS Inc. (Chicago, IL, USA). The analysis of the transfection efficiency of CDplexes was performed with a two-tailed unpaired Student´s t-test. $P < 0.05$ was considered statistically significant.

Synthesis

Compounds 2-[*N,N*-bis(2-(*N*-*tert*-butoxyaminocarbonyl)ethylamino]ethyl isothiocyanate (**11**),[1] 6,6'-dideoxy-6,6'-diisothiocyanato-2,3,4,2',3',4'-hexa-*O*-hexanoyl-α,α'-trehalose (**12**)[2] 6,6'-dideoxy-6,6'-diisothiocyanato-2,3,4,2',3',4'-hexa-*O*-tetradecanoyl-α,α'-trehalose (**13**),[3] 6,6'-diazido-6,6'-dideoxy-α,α'-trehalose (**16**),[4] 6,6'-diamino-6,6'-dideoxy-2,3,4,2',3',4'-hexa-*O*-(3-(2-*N*-*tert*-butoxycarbonylaminoethylthio)propyl)-α,α'-trehalose (**20**)[2] and *N*^{I},*N*^{II}-Bis-[6,6'-dideoxy-2,3,4,2',3',4'-2-*N*-*tert*-butoxycarbonylaminoethyl-thiopropyl-6'-isothiocyanato-α,α'-trehalos-6-yl]thiourea (**30**),[5] were prepared according to reported procedures.

Synthesis of the C_1L_2 MM-MNPs 1-6

*Preparation of the acylated tetrasaccharidic diisothiocyanates **14** and **15***

A solution of the corresponding trehalose diisothiocyanate derivative **12** or **13** (0.395 mmol) in pyridine-H_2O (10:1, 11 mL) was stirred at 45 ºC for 4 h. After evaporation of the solvents, the resulting residue was purified by column chromatography using the solvent indicated in each case (Scheme S1).

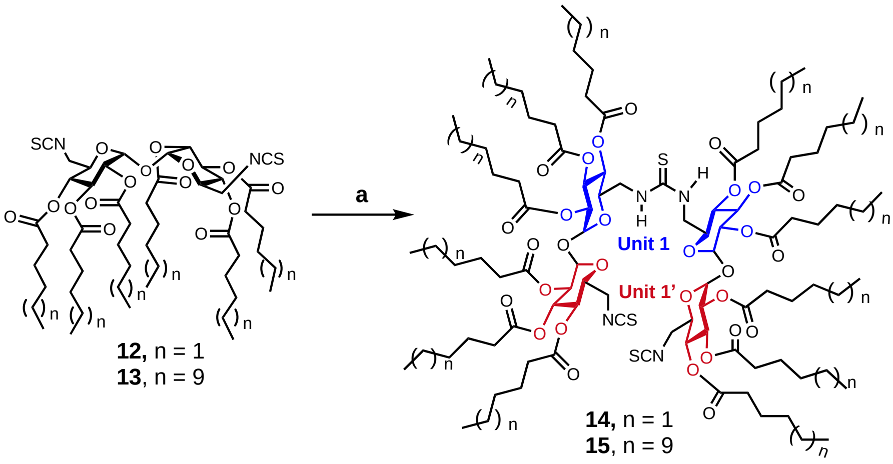

Scheme S1. Synthesis of dimeric precursors of α,α´-trehalose **14** y **15**. Reagents and conditions: (a) 10:1 pyridine-H_2O, 45 ºC, 4 h, 20% (47% y 33% of starting material is recovered, respectively). Unit 1and unit 1' refers to spin systems for NMR assignments.

*N^I,N^{II}-Bis-[6,6'-dideoxy-2,3,4,2',3',4'-hexa-O-hexanoyl-6'-isothiocyanato-α,α'-trehalos-6-yl]thiourea (**14**).*

Compound **14** was obtained from **12** following the general procedure above described. Column chromatography 1:6 EtOAc-cyclohexane. Yield: 155 mg (20%; 47% of unreacted starting material recovered). R_f = 0.26 (1:4 EtOAc-cyclohexane); $[\alpha]_D$ = +90.8 (*c* 1.0, DCM); IR (ATR): υ_{max} = 2077 cm^{-1}; UV (MeOH): λ_{max} 243 nm (ε_{mM} = 45.2). ^{1}H NMR (500 MHz, $CDCl_3$, 323 K): δ = 6.17 (bd, 2 H, NH), 5.60 (d, 2 H, $J_{1',2'}$ = 4.0 Hz, H-1'), 5.56 (t, 2 H, $J_{2',3'}$ = $J_{3',4'}$ = 9.7 Hz, H-3'), 5.49 (t, 2 H, $J_{2,3}$ = $J_{3,4}$ = 9.7 Hz, H-3), 5.40 (d, 2 H, $J_{1,2}$ = 4.0 Hz, H-1), 5.12 (dd, 2 H, H-2), 5.03 (t, 2 H, $J_{4',5'}$ = 9.7 Hz, H-4'), 4.99 (t, 2 H, $J_{4,5}$ = 9.7 Hz, H-4), 4.94 (dd, 2 H, H-2'), 4.04 (ddd, 2 H, $J_{5',6'a}$ = 6.3 Hz, $J_{5',6'b}$ = 3.7 Hz, H-5'), 3.61 (bdd, 2 H, $J_{6a,6b}$ = 14.0 Hz, $J_{5,6a}$ = 6.3 Hz, H-6a), 4.04 (td, 2 H, $J_{5,6b}$ = 8.0 Hz, H-5), 3.64 (bdd, 2 H, $J_{6'a,6'b}$ = 14.6 Hz, H-6'a), 3.54 (dd, 2 H, H-6'b), 3.33 (ddd, 2 H, $J_{NH,6b}$ = 3.4 Hz, H-6b), 2.37-2.23 (m, 24 H, CH_2CO), 1.67-1.55 (m, 24 H, CH_2),1.37-1.28 (m, 48 H, CH_2), 0.94-0.90 (m, 36 H, CH_3). ^{13}C NMR (125.7 MHz, $CDCl_3$, 323 K): δ = 184.7 (CS), 173.6, 172.3, 172.2, 172.1 (CO), 136.6 (NCS), 92.2 (C-1), 91.9 (C-1'), 70.7 (C-2'), 69.8 (C-5), 69.7 (C-3, C-2, C-4'), 69.3 (C-4), 69.1 (C-3'), 68.6 (C-5'), 46.0 (C-6), 45.1 (C-6'), 33.9 (CH_2), 31.2 (CH_2), 24.4 (CH_2), 22.2 (CH_2), 13.8 (CH_3). ESI-MS: *m/z* = 2006.9 $[M + Na]^+$. Anal. Calcd for $C_{99}H_{162}N_4O_{30}S_3$: C, 59.92; H, 8.23; N, 2.82; S, 4.85. Found: C, 60.17; H, 8.38; N, 2.67; S, 4.63.

*N^I,N^{II}-Bis-[6,6'-dideoxy-2,3,4,2',3',4'-hexa-O-myristoyl-6'-isothiocyanato-α,α'-trehalos-6-yl]thiourea (**15**).*

Compound **15** was obtained from **13** following the general procedure above described. Column chromatography cyclohexane → 1:6 acetone-cyclohexane. Yield: 87 mg (20%; 33% of unreacted starting material. R_f = 0.25 (1:6 EtOAc-cyclohexane); $[\alpha]_D$ = +77.1 (*c* 1.0, DCM); IR (ATR): υ_{max} = 2082 cm^{-1}; UV (DCM): λ_{max} 249 nm (ε_{mM} = 18.0). ^1H NMR (500 MHz, $CDCl_3$, 323 K): δ = 6.18 (m, 2 H, NH), 5.62 (d, 2 H, $J_{1,2}$ = 3.5 Hz, H-1), 5.56 (t, 2 H, $J_{2',3'}$ = $J_{3',4'}$= 9.5 Hz, H-3'), 5.49 (t, 2 H, $J_{2,3}$ = $J_{3,4}$ = 9.5 Hz, H-3), 5.40 (d, 2 H, $J_{1',2'}$ = 3.0 Hz, H-1'), 5.13 (dd, 2 H, H-2), 5.04 (t, 2 H, $J_{4',5'}$ = 9.5 Hz, H-4'), 4.97 (t, 2 H, H-4), 4.92 (dd, 2 H, $J_{2',3'}$ = 9.5 Hz, H-2'), 4.06-4.02 (m, 2 H, H-5), 3.96 (m, 2 H, H-6a), 3.89-3.85 (m, 2 H, H-5'), 3.67-3.62 (m, 2 H, H-6a'), 3.57-3.53 (m, 2 H, H-6b'), 3.34 (m, 2 H, H-6b), 2.38-2.25 (m, 24 H, CH_2CO), 1.62-1.55 (m, 24 H, CH_2), 1.30 (m, 240 H, CH_2), 0.91 (m, 36 H, CH_3). ^{13}C NMR (125.7 MHz, $CDCl_3$, 323K): δ = 184.7 (CS), 172.2 (CO), 135.5 (NCS), 92.3 (C-1), 91.8 (C-1'), 70.7 (C-2'), 69.7 (C-2), 69.8 (C-4'), 69.7 (C-5), 69.7 (C-3), 69.2 (C-4), 69.1 (C-3'), 68.6 (C-5'), 46.0 (C-6'), 45.1 (C-6), 34.0 (CH_2CO), 24.8 (CH_2), 13.9 (CH_3). MALDI-TOF MS: *m/z* = 3354.9 $[M + Na]^+$. Anal. Calcd for $C_{195}H_{354}N_4O_{30}S_3$: C, 70.31; H, 10.71; N, 1.68; S, 2.89. Found: C, 70.52; H, 10.88; N, 1.46; S, 2.61.

*Preparation of the hexylated tetrasaccharidic diisothiocyanate **19**.*

Compound **19** was obtained by hexylation of 6,6'-diazido-6,6'-dideoxy-α,α'-trehalose (**16**) (→**17**), subsequente isothiocyanation of the azido groups in **16** by reaction with triphenylphosphine (TPP)-carbon disulfide (CS_2) to give the α,α´-trehalose diisothiocyanate **18** and controlled self-condensation of the later in pyridine-water (Scheme S2).

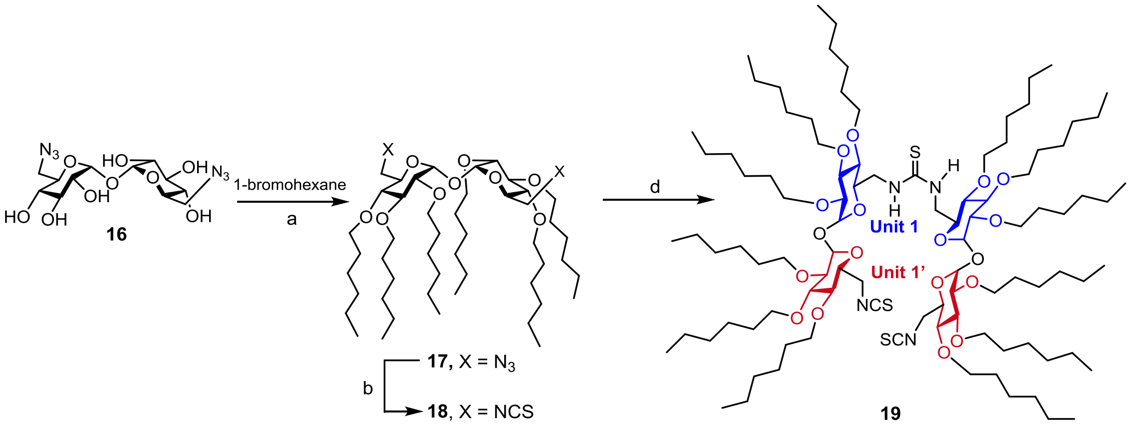

Scheme S2. Synthesis of hexylated tetrasaccharidic diisothiocyanate **19**. Reagents and conditions: (a) NaH, DMF, 60 ºC, 24 h, 75%. (b) TPP, THF, NH_4OH, 50 ºC, 24 h, 58-59%. (c) TPP, CS_2, dioxane, 24 h, 90%. (d) 10:1 pyridine-H_2O, 45 ºC, 4 h, 30% (51% starting material recovered). Unit 1and unit 1' refer to spin systems for NMR assignments.

*6,6'-Diazido-6,6'-dideoxy-2,3,4,2',3',4'-hexa-O-hexyl-α,α'-trehalose (**17**)*

To a solution of **16** (471 mg, 1.20 mmol) in dry DMF (9.50 mL), NaH (777 mg, 32.4 mmol) was added and the mixture was stirred at 0 ºC for 10 min. 1-Bromohexane (4.54 mL, 32.4 mmol) was added dropwise, under Ar atmosphere, and the mixture was stirred 24 h at 60 ºC. The reaction was quenched with MeOH (5 mL) and stirred for 10 min. Solvents were removed and the resulting residue was suspended in DCM (50 mL). The suspension was washed with H_2O (3 x 15 mL) and the organic layer was dried ($MgSO_4$), filtered, concentrated and purified by column chromatography (1:20 EtOAc-cyclohexane). Yield: 1.15 g (75%). R_f = 0.57 (1:10 EtOAc-cyclohexane); $[\alpha]_D$ = +97.0 (c 1.0, DCM). ^{1}H NMR (300 MHz, $CDCl_3$): δ = 5.11 (d, 2 H, $J_{1,2}$ = 3.5 Hz, H-1), 4.04 (ddd, 2 H, $J_{4,5}$ = 9.5 Hz, $J_{5,6a}$ = 4.2 Hz, $J_{5,6b}$ = 2.9 Hz, H-5), 3.81 (m, 4 H, CH_2O), 3.64-3.34 (m, 14 H, CH_2O, H-3, H-6a, H-6b), 3.25 (dd, 2 H, $J_{2,3}$ = 9.5 Hz, H-2), 3.16 (t, 2 H, $J_{3,4}$ = 9.5 Hz, H-4), 1.62-1.48 (m, 12 H, CH_2), 1.34-1.24 (bs, 36 H, CH_2), 0.88 (m, 18 H, CH_3). ^{13}C NMR (75.5 MHz, $CDCl_3$): δ = 93.5 (C-1), 81.6, 80.3 (C-3, C-2), 78.7 (C-4), 73.5, 73.3, 71.9 (CH_2O), 70.6 (C-5), 51.5 (C-6), 31.8, 31.7, 30.6, 30.3, 30.2, 25.9, 25.8, 22.6 (CH_2), 14.0 (CH_3). ESI-MS: *m/z* = 919.8 $[M + Na]^+$. Anal. Calcd for $C_{48}H_{92}N_6O_9$: C, 64.25; H, 10.33; N, 9.37. Found: C, 64.31; H, 10.27; N, 9.17.

*6,6'-Dideoxy-6,6'-diisothiocyanato-2,3,4,2',3',4'-hexa-O-hexyl-α,α'-trehalose (**18**)*

To a solution of **17** (1.19 g, 1.32 mmol) in dioxane (50 mL), TPP (761 mg, 2.90 mmol) and CS_2 (1.94 mL, 26.4 mmol) were added, under Ar atmosphere. The solution was stirred at rt for 24 h. Then the solvents were evaporated and the residue was purified by column chromatography (cyclohexane → 1:100 EtOAc-cyclohexane). Yield: 1.10 g (90%); R_f = 0.64 (1:10 EtOAc-cyclohexane); $[\alpha]_D$ = +63.7 (*c* 1.0, DCM); IR(ATR): υ_{max} = 2087 cm^{-1}. ^{1}H NMR (400 MHz, $CDCl_3$): δ = 5.23 (d, 2 H, $J_{1,2}$ = 3.2 Hz, H-1), 4.09-4.03 (m, 2 H, H-5), 3.92-3.52 (m, 18 H, CH_2O, H-3, H-6a, H-6b), 3.30 (dd, 2 H, $J_{2,3}$ = 10.1 Hz, H-2), 3.15 (t, 2 H, $J_{3,4}$ = 8.0 Hz, H-4), 1.63-1.53 (m, 12 H, *C*H_2), 1.33 (bs, 36 H, CH_2), 0.91 (m, 18 H, CH_3). ^{13}C NMR (100.6 MHz, $CDCl_3$): δ = 133.1 (NCS), 93.5 (C-1), 81.3 (C-3), 80.4 (C-2), 78.8 (C-4), 73.5 (CH_2O), 69.5 (C-5), 46.1 (C-6), 31.5, 30.9, 26.3, 22.5 (CH_2), 14.3 (CH_3). ESI-MS: *m/z* = 951.6 $[M + Na]^+$. Anal. Calcd for $C_{50}H_{92}N_2O_9S_2$: C, 64.62; H, 9.98; N, 3.01; S, 6.90. Found: C, 64.77; H, 10.21; N, 2.86; S, 6.69.

*N^I,N^{II}-Bis-[6,6'-dideoxy-2,3,4,2',3',4'-hexa-O-hexyl-6'-isothiocyanato-α,α'-trehalos-6-yl]thiourea (**19**).*

A solution of the tetrasaccharidic diisothiocyanate **18** (0.395 mmol) in pyridine-H_2O (10:1, 11 mL) was stirred at 45 ºC for 4 h. After evaporation of the solvents, the resulting residue was purified by column chromatography (cyclohexane → 1:10 EtOAc-cyclohexane). Yield: 153 mg (30%; 51% of unreacted starting material recovered). R_f = 0.19 (1:10 EtOAc-cyclohexane); $[\alpha]_D$ = +49.0 (*c* 1.0, DCM); IR (ATR): υ_{max} = 2085 cm^{-1}; UV (MeOH): λ_{max} 247 nm (ε_{mM} = 4.1). ^{1}H

NMR (400 MHz, $CDCl_3$): δ = 5.12 (d, 4 H, $J_{1,2} = J_{1',2'}$ = 3.2 Hz, H-1, H-1'), 4.08-4,00 (m, 4 H, H-5, H-5'), 3.90-3.53 (m, 36 H, C*H*$_2$O, H-3, H-3', H-6, H-6'), 3.29-3.25 (dd, 4 H, $J_{2,3} = J_{2',3'}$ = 9.0 Hz, H-2, H-2'), 3.21-3.08 (m, 4 H, H-4, H-4'), 1.59 (m, 24 H, CH_2), 1.36 (bs, 72 H, CH_2), 0.94-0.82 (m, 36 H, CH_3). ^{13}C NMR (100.6 MHz, $CDCl_3$): δ = 93.5 (C-1, C-1'), 81.5 (C-3, C-3'), 80.1 (C-2, C-2'), 78.9 (C-4, C-4'), 73.5, 72.4 (*C*H$_2$O), 69.5 (C-5, C-5'), 46.4 (C-6, C-6'), 31.9, 30.5, 25.9, 22.5 (CH_2), 14.1 (CH_3). ESI-MS: *m/z* = 1839.1 $[M + Na]^+$. Anal. Calcd for $C_{99}H_{186}N_4O_{18}S_3$: C, 65.45; H, 10.32; N, 3.08; S, 5.29. Found: C, 65.58; H, 10.40; N, 2.91; S, 5.14.

*Preparation of the unbranched C_1L_2 MM-MNP representatives **1-3**.*

Condensation reactions involving the hexacysteaminylated α,α'-trehalose diamine **20** (0.045 mmol) and the tetrasaccharidic diisothiocyanates **14**, **15** or **19** (0.045 mmol) in pyridine-dimethylaminopyridine (DMAP) at 40 ºC for 24 h, followed by column chromatography in the solvent indicated in each case, afforded the Boc-protected CT3 patchy macrocycles **21-23**, respectively. Trifluoroacetic acid (TFA)-promoted hydrolysis of the carbamate groups and final lyophilization from 0.1 M HCl (x 2) provided the target cationic Mickey Mouse molecular nanoparticles **1-3** in quantitative yield (Scheme S3).

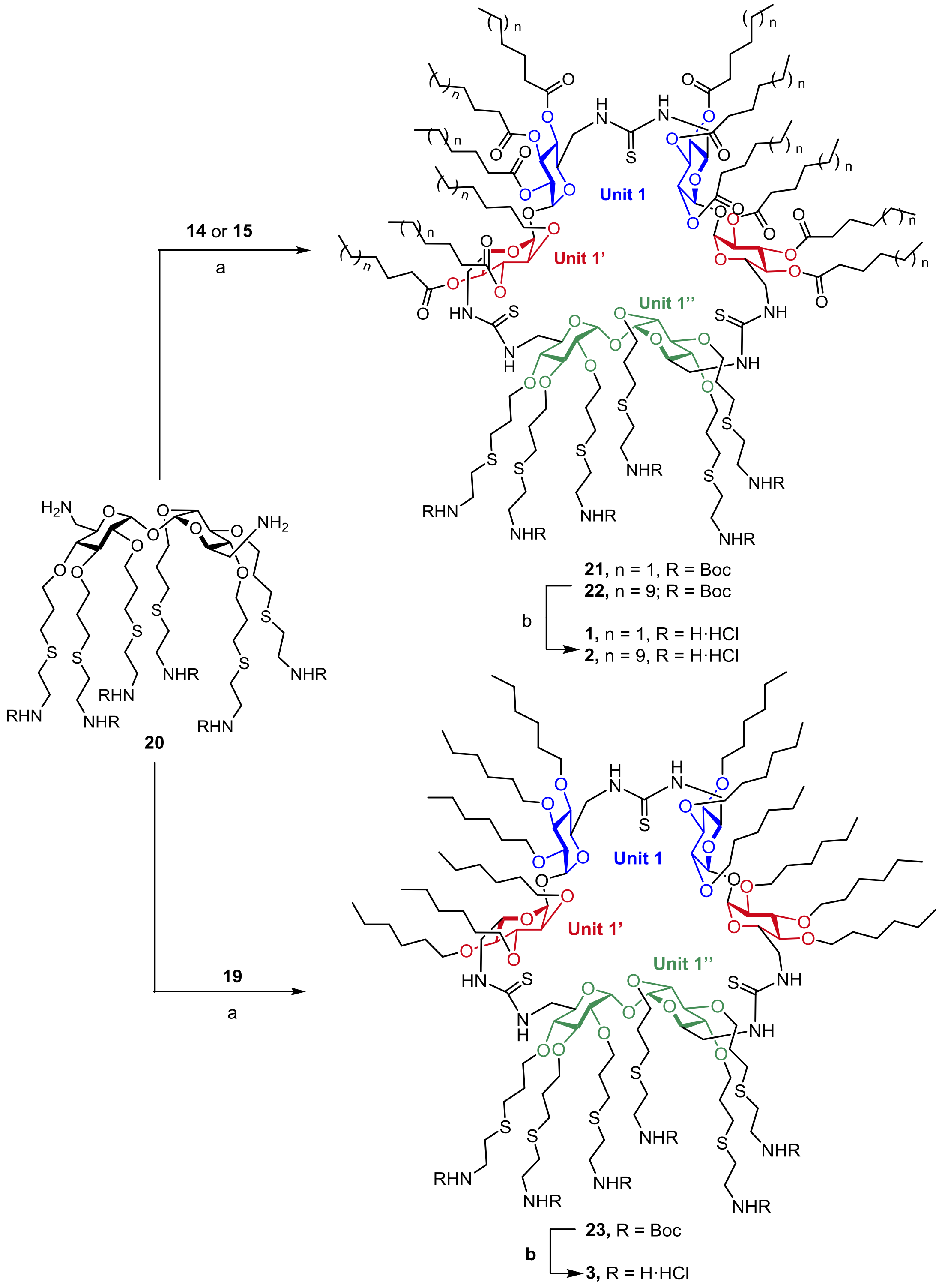

Scheme S3. Synthesis of the unbranched C_1L_2 MM-MNPs **1**-**3**. Reagents and conditions: (a) DMAP, pyridine, 40 ºC, 24 h, 44-62%. (b) 1:1 TFA-DCM, r.t., 1 h, quantitative. Unit 1, unit 1' and unit 1'' refer to spin systems for NMR assignments.

*Hexanoylated Boc-protected CT3 derivative **21***

Compound **21** was obtained from **14** and **20** following the general procedure above described. Column chromatography 1:1→ 2:1 EtOAc-cyclohexane. Yield: 100 mg (48%). R_f = 0.55 (1:1 EtOAc-cyclohexane); $[\alpha]_D$ = +89.0 (*c* 1.0, DCM); UV (MeOH): λ_{max} 246 nm (ε_{mM} = 30.4). ^{1}H NMR (500 MHz, $CDCl_3$, 323 K): δ = 6.37, 6.21 (2 bs, 6 H, NHCS), 5.51 (m, 4 H, H-3', H-3"), 5.43 (bs, 2 H, H-1'), 5.38 (bs, 2 H, H-1"), 5.23-5.03 (m, 6 H, NHBoc), 5.06 (bs, 2 H, H-1), 4.99-4.91 (m, 8 H, H-2', H-2", H-4', H-4"), 4.04 (m, 4 H, H-6'a, H-6"a), 3.98-3.62 (m, 12 H, CH_2O), 3.92-3.87 (m, 6 H, H-5, H-5', H-5"), 3.83 (m, 2 H, H-6a), 3.68 (m, 2 H, H-6b), 3.60 (t, 2 H, $J_{2,3}$ = $J_{3,4}$ = 9.7 Hz, H-3), 3.44 (bs, 4 H, H-6'b, H-6"b), 3.32 (m, 12 H, CH_2N), 3.28 (dd, 2 H, $J_{1,2}$ = 2.5 Hz, H-2), 3.07 (t, 2 H, $J_{4,5}$ = 9.7 Hz, H-4), 2.70-2.63 (m, 24 H, SCH_2), 2.38-2.23 (m, 24 H, CH_2), 1.92-1.83 (m, 12 H, CH_2), 1.65-1.56 (m, 24 H, CH_2), 1.47, 1.48, 1.46 (3 s, 54 H, C*Me*$_3$), 1.36-1.29 (m, 48 H, CH_2), 0.94-0.90 (m, 36 H, CH_3). ^{13}C NMR (100.6 MHz, $CDCl_3$, 323 K): δ = 184.6, 184.2 (CS), 173.7, 173.4, 172.4, 172.1 (CO ester), 156.0, 155.7 (CO carbamate), 92.9 (C-1), 91.9 (C-1', C-1"), 81.0 (C-3), 80.1 (C-2), 79.5 (C-4), 79.3 (*C*Me$_3$), 71.7, 71.6 (C-5, CH_2O), 70.6, 70.4 (C-2', C-2"), 69.9, 69.8 (C-5', C-5'', CH_2O), 69.4 (C-3', C-3"), 68.9 (C-4', C-4"), 45.4, 44.8, 44.6 (C-6, C-6', C-6"), 40.1 (CH_2N), 34.1, 34.0 (CH_2), 32.3 (SCH_2), 31.2, 30.7, 30.3, 30.1, 29.6 (CH_2), 28.6 (CH_2S), 28.4 (C*Me*$_3$), 24.5, 24.4, 22.2 (CH_2), 13.7, 13.6 (CH_3). ESI-MS: *m/z* = 3652.0 $[M + Na]^+$, 1836.8 $[M + 2 Na]^{2+}$. Anal. Calcd for $C_{171}H_{300}N_{12}O_{51}S_9$: C, 56.60; H, 8.33; N, 4.63; S, 7.95. Found: C, 56.73; H, 8.47; N, 4.45; S, 7.52.

Myristoylated Boc-protected CT3 derivative ***22***

Compound **22** was obtained from **15** and **20** following the general procedure above described. Column chromatography 1:4→1:1 EtOAc-cyclohexane. Yield: 83 mg (44%); R_f = 0.65 (1:1 EtOAc-cyclohexane); $[\alpha]_D$ = +61.0 (*c* 1.0, DCM); UV (DCM): λ_{max} 248 nm (ε_{mM} = 44.0). ^{1}H NMR (500 MHz, $CDCl_3$, 333 K): δ = 6.34, 6.16 (2 bs, 6 H, NHCS), 5.51 (m, 4 H, H-3, H-3'), 5.42, 5.38 (2 d, 4 H, H-1, H-1'), 5.19 (m, 6 H, NHBoc), 5.06 (bs, 2 H, H-1"), 4.99-4.90 (m, 8 H, H-2, H-2', H-4, H-4'), 4.03 (m, 4 H, H-6a, H-6'a), 3.92-3.85 (m, 12 H, CH_2O), 3.87-3.81 (m, 6 H, H-5, H-5', H-5"), 3.68 (m, 4 H, H-6"a, H-6"b), 3.60 (t, 2 H, $J_{2'',3''}$ = $J_{3'',4''}$ = 9.7 Hz, H-3"), 3.44 (bs, 4 H, H-6b, H-6'b), 3.32 (m, 12 H, CH_2N), 3.27 (dd, 2 H, $J_{1'',2''}$ = 2.5 Hz, H-2"), 3.07 (t, 2 H, $J_{4'',5''}$ = 9.7 Hz, H-4"), 2.71-2.64 (m, 24 H, SCH_2), 2.40-2.35 (m, 24 H, CH_2CO), 1.93-1.87 (m, 12 H, C*H*$_2$CH$_2$O), 1.62-1.59 (m, 24 H, CH_2), 1.48 (s, 54 H, C*Me*$_3$), 1.30 (m, 240 H, CH_2), 0.93-0.90 (m, 36 H, CH_3). ^{13}C NMR (125.7 MHz, $CDCl_3$): δ = 184.2 (CS), 173.9, 173.5, 172.4, 172.3 (CO ester), 156.0, 155.8 (CO carbamate), 91.5 (C-1, C-1', C-1"), 80.8 (C-3"), 79.9 (C-2"), 79.6 (C-4"), 79.3 (*C*Me$_3$), 71.7, 71.6 (C-5", CH_2O), 70.3 (C-2, C-2'), 69.6 (C-5, C-5', CH_2O), 69.2 (C-3, C-3'), 68.7 (C-4, C-4'), 45.2, 44.4 (C-6, C-6', C-6"), 39.9 (CH_2N), 34.1, 34.0 (*C*H$_2$CO), 31.9 (SCH_2), 29.7, 29.6, 29.5, 29.4 (CH_2), 29.2 (CH_2S), 28.4 (C*Me*$_3$), 24.8, 22.7 (CH_2), 14.1 (CH_3). ESI-MS:

m/z = 2509.92 [M + 2 Na]$^{2+}$. Anal. Calcd for $C_{267}H_{492}N_{12}O_{51}S_9$: C, 64.46; H, 9.97; N, 3.38; S, 5.80. Found: C, 64.70; H, 10.18; N, 3.26; S, 5.63.

Hexylated Boc-protected CT3 derivative ***23***

Compound **23** was obtained from **19** and **20** following the general procedure above described. Column chromatography 1:1 EtOAc-cyclohexane→45:5:3 EtOAc-EtOH-H_2O). Yield: 112 mg (62%). R_f = 0.66 (45:5:3 EtOAc-EtOH-H_2O); $[\alpha]_D$ = +52.8 (*c* 1.0, DCM); UV (MeOH): λ_{max} 243 nm (ε_{mM} = 46.1). ^{1}H NMR (400 MHz, $CDCl_3$, 323 K): δ = 6.17, 6.12 (2 bs, 6 H, NHCS), 5.20 (bs, 6 H, NHBoc), 5.03 (m, 6 H, H-1, H-1', H-1''), 4.00 (m, 6 H, H-5, H-5', H-5''), 3.83-3.57 (m, 30 H, H-6, H-6', H-6'', CH_2O, H-3, H-3', H-3''), 3.33 (m, 12 H, CH_2N), 3.20 (m, 6 H, H-2, H-2', H-2''), 3.06 (m, 6 H, H-4, H-4', H-4''), 2.70-2.62 (m, 24 H, CH_2S), 1.92-1.85 (m, 12 H, CH_2), 1.60 (m, 48 H, CH_2), 1.47 (s, 54 H, C*Me*$_3$), 1.34-1.29 (m, 48 H, CH_2), 0.91 (m, 36 H, CH_3). ^{13}C NMR (100.6 MHz, $CDCl_3$, 323 K): δ = 184.0, 183.8 (CS), 156.8, 155.7 (CO carbamate), 93.2, 92.8 (C-1, C-1', C-1''), 81.0 (C-3, C-3', C-3''), 80.1 (C-2, C-2', C-2''), 79.3 (*C*Me$_3$), 79.2 (C-4, C-4', C-4''), 73.5 (CH_2O), 71.9, 69.9 (C-5, C-5', C-5''), 45.4 (C-6, C-6', C-6''), 40.1 (CH_2N), 34.1, 34.0 (CH_2), 32.3 (SCH_2), 31.7 (C-4_{Hex}), 30.4 (CH_2), 29.6 (CH_2S), 28.4 (C*Me*$_3$), 24.8, 22.5 (CH_2), 13.9 (CH_3). ESI-MS: *m/z* = 3483.6 [M + Na]$^+$, 1752.9 [M + 2 Na]$^{2+}$. Anal. Calcd for $C_{171}H_{324}N_{12}O_{39}S_9$: C, 59.34; H, 9.44; N, 4.86; S, 8.34. Found: C, 59.16; H, 9.19; N, 4.61; S, 8.07.

Hexanoylated C_1L_2 MM-MNP ***1***

Compound **21** (80 mg, 0.022 mmol) was treated with 1:1 TFA-DCM (4 mL) at rt for 1 h. Then, the solvent was eliminated under pressure and coevaporated several times with toluene. The residue was dissolved in 0.1 M HCl and freeze-dried (x 2) to yield **1** as the corresponding hexahydrochloride salt. Yield: 71 mg (quantitative). $[\alpha]_D$ = +72.5 (*c* 1.0, DCM); UV (MeOH): λ_{max} 246 nm (ε_{mM} = 22.9). ^{1}H NMR (500 MHz, 5:1 CD_3OD-$CDCl_3$, 333 K): δ = 5.28 (m, 4 H, H-3', H-3''), 5.25 (d, 2 H, $J_{1',2'}$ = 4.0 Hz, H-1'), 5.19 (d, 2 H, $J_{1'',2''}$ = 4.0 Hz, H-1''), 5.09 (bd, 2 H, H-1), 4.88-4.81 (m, 4 H, H-2', H-2'') 4.72-4.67 (m, 4 H, H-4', H-4''), 4.21 (bd, 4 H, H-6'a, H-6''a), 3.90 (bd, 2 H, H-6a), 3.81-3.42 (m, 12 H, CH_2O), 3.74 (bd, 2 H, H-6b), 3.69-3.56 (m, 4 H, H-5', H-5''), 3.52 (m, 2 H, H-5), 3.38 (t, 2 H, $J_{2,3}$ = $J_{3,4}$ = 9.1 Hz, H-3), 3.12 (dd, 2 H, $J_{1,2}$ = 3.4 Hz, H-2), 2.99-2.91 (m, 16 H, H-6'b, H-6''b, CH_2N), 2.83 (t, 2 H, $J_{4,5}$ = 9.7 Hz, H-4), 2.67-2.64 (m, 12 H, SCH_2), 2.53-2.40 (m, 12 H, CH_2S), 2.21-1.98 (m, 24 H, CH_2), 1.72-1.59 (m, 12 H, CH_2), 1.46-1.31 (m, 24 H, CH_2), 1.14-1.07 (m, 48 H, CH_2), 0.72-0.67 (m, 36 H, CH_3).^{13}C NMR (125.7 MHz, 5:1 CD_3OD-$CDCl_3$, 333 K): δ = 183.7, 183.4 (CS), 172.6, 172.5, 172.4, 172.3 (CO), 90.9 (C-1), 90.3, 90.1 (C-1', C-1''), 80.7 (C-3), 79.7 (C-2), 79.5 (C-4), 71.1, 70.9 (C-5, CH_2O), 70.1, 70.0, 69.8 (C-2', C-2'', C-5', C-5''), 69.7, 69.5, 69.3 (CH_2O, C-3', C-3''), 69.1, 68.9 (C-4', C-4''), 45.0, 44.5, 44.1 (C-6, C-6', C-6''), 38.9, 38.8 (CH_2N), 33.8-33.6 (CH_2), 30.9 (CH_2), 30.8

(C-4$_{Hex}$), 30.2, 30.1, 29.9 (CH_2), 29.1, 28.7, 28.6 (SCH_2), 28.5, 28.3, 28.2 (CH_2S), 24.1, 24.0, 21.7 (CH_2), 13.1, 13.0 (CH_3). ESI-MS: *m/z* = 1514.8 [M + 2 H]$^{2+}$. Anal. Calcd for $C_{141}H_{252}N_{12}O_{39}S_9$·6 HCl: C, 52.16; H, 8.01; N, 5.18; S, 8.89. Found: C, 51.85; H, 7.78; N, 4.81; S, 8.50.

Myristoylated C_1L_2 MM-MNP **2**

Compound **22** (44 mg, 8.8 μmol) was treated with 1:1 TFA-DCM (8 mL) at rt for 1 h. Then, the solvent was eliminated under pressure and coevaporated several times with toluene. The residue was dissolved in 0.1 M HCl and freeze-dried (x 2) to yield **2** as the corresponding hexahydrochloride salt. Yield: 40 mg (quantitative); R_f = 0.15 (45:5:3 EtOAc-EtOH-H_2O); $[\alpha]_D$ = +39.4 (*c* 1.0, DCM); UV (DCM): λ_{max} 244 nm (ε_{mM} = 71.7). ^{1}H NMR (500 MHz, CD_3OD, 323 K): δ = 5.57-5.50 (m, 4 H, H-3, H-3'), 5.48 (m, 2 H, H-1), 5.39 (m, 2 H, H-1'), 5.14-5.07 (m, 4 H, H-2, H-2'), 5.00-4.93 (m, 4 H, H-4, H-4'), 4.45 (m, 4 H, H-6a, H-6a'), 4.21 (m, 4 H, H-6b, H-6b'), 3.94-3.67 (m, 24 H, CH_2O, H-5, H-5', H-5", H-6a", H-6b", H-3"), 3.42-3.40 (m, 2 H, H-2"), 3.33 (m, 12 H, CH_2N), 3.10-3.06 (t, 2 H, H-4"), 2.90-2.86 (m, 12 H, SCH_2), 2.77-2.66 (m, 12 H, CH_2S), 2.43-2.24 (m, 24 H, CH_2CO), 1.95-1.86 (m, 12 H, CH_2), 1.64-1.60 (m, 24 H, CH_2), 1.33 (m, 240 H, CH_2), 0.94-0.93 (m, 36 H, CH_3). ^{13}C NMR (100.6 MHz, CD_3OD, 323 K): δ = 183.4 (CS), 172.4 (CO), 90.2 (C-1, C-1', C-1"), 81.1 (C-3"), 79.7 (C-2"), 79.6 (C-4"), 71.1, 70.9 (C-5", CH_2O), 70.1 (C-2, C-2'), 69.6 (C-5, C-5', CH_2O), 69.0 (C-3, C-3'), 68.6 (C-4, C-4'), 45.1 (C-6, C-6', C-6"), 39.1 (CH_2N), 34.1, 34.0 (CH_2CO), 31.6 (SCH_2), 30.2, 29.7, 29.5, 29.2, 29.0, 28.9 (CH_2), 28.1, 27.9 (CH_2S), 24.5, 22.3 (CH_2), 13.1 (CH_3). ESI-MS: *m/z* = 2187.12 [M + 2 H]$^{2+}$. Anal. Calcd for $C_{237}H_{444}N_{12}O_{39}S_9$·6 HCl: C, 61.97; H, 9.87; N, 3.66; S, 6.28. Found: C, 61.72; H, 9.95; N, 3.41; S, 5.99.

Hexylated C_1L_2 MM-MNP **3**

Compound **23** (118 mg, 0.034 mmol) was treated with 1:1 TFA-DCM (4 mL) at rt for 1 h. Then, the solvent was eliminated under pressure and coevaporated several times with toluene. The residue was dissolved 0.1 M HCl and freeze-dried (x 2) to yield **3** as the corresponding hexahychloride salt. Yield: 102 mg (97%). $[\alpha]_D$ = +48.0 (*c* 1.0, MeOH); UV (MeOH): λ_{max} 241 nm (ε_{mM} = 5). ^{1}H NMR (500 MHz, CD_3OD, 323 K): δ = 5.39 (bs, 12 H, NH_2), 5.34 (d, 6 H, $J_{1,2}$ = 3.1 Hz, H-1, H-1', H-1"), 3.99 (m, 6 H, H-5, H-5',H-5"), 3.95-3.56 (m, 30 H, H-6, H-6', H-6", CH_2O, H-3, H-3', H-3"), 3.37 (dd, 6 H, $J_{2,3}$ = 9.5 Hz, H-2, H-2', H-2"), 3.31-3.28 (m, 6 H, H-4, H-4', H-4"), 3.19 (m, 12 H, CH_2N), 2.80-2.68 (m, 24 H, CH_2S), 1.96-1.88 (m, 12 H, CH_2), 1.60 (m, 48 H, CH_2), 1.42-1.30 (m, 48 H, CH_2), 0.93 (m, 36 H, CH_3). ^{13}C NMR (125.7 MHz, $CDCl_3$): δ = 92.5 (C-1, C-1', C-1"), 80.9 (C-3, C-3', C-3"), 80.2 (C-2, C-2', C-2"), 73.5 (C-4, C-4', C-4"), 71.8 (CH_2O), 69.6 (C-5, C-5', C-5"), 45.6 (C-6, C-6', C-6"), 39.3 (CH_2N), 33.6 (CH_2), 31.8 (SCH_2), 30.7, 30.4 (CH_2), 29.6 (CH_2S), 25.8, 22.6 (CH_2), 14.1 (CH_3). ESI-MS: *m/z* = 1462.1 [M

+ 2 Na]$^{2+}$. Anal. Calcd for $C_{141}H_{276}N_{12}O_{27}S_9 \cdot 6\ HCl$: C, 55.00; H, 9.23; N, 5.46; S, 9.37. Found: C, 54.74; H, 8.97; N, 5.12; S, 9.01.

Preparation of the dendronized C_1L_2 MM-MNP representatives ***4-6***

To a solution of **1-3** (0.045 mmol) in pyridine (4 mL), a solution of **11**[1] (0.045 mmol) and DMAP (17 mg, 0.135 mmol) in pyridine (4 mL) was slowly added and the mixture was stirred at 40 ºC overnight. Column chromatography with the solvent indicated in each case afforded the dendronized Boc-protected CT3 patchy macrocycles **24-26**, respectively. Trifluoroacetic acid (TFA)-promoted hydrolysis of the carbamate groups and final lyophilization from 0.1 M HCl (x 2) provided the target cationic Mickey Mouse molecular nanoparticles **4-6** in quantitative yield (Scheme S4).

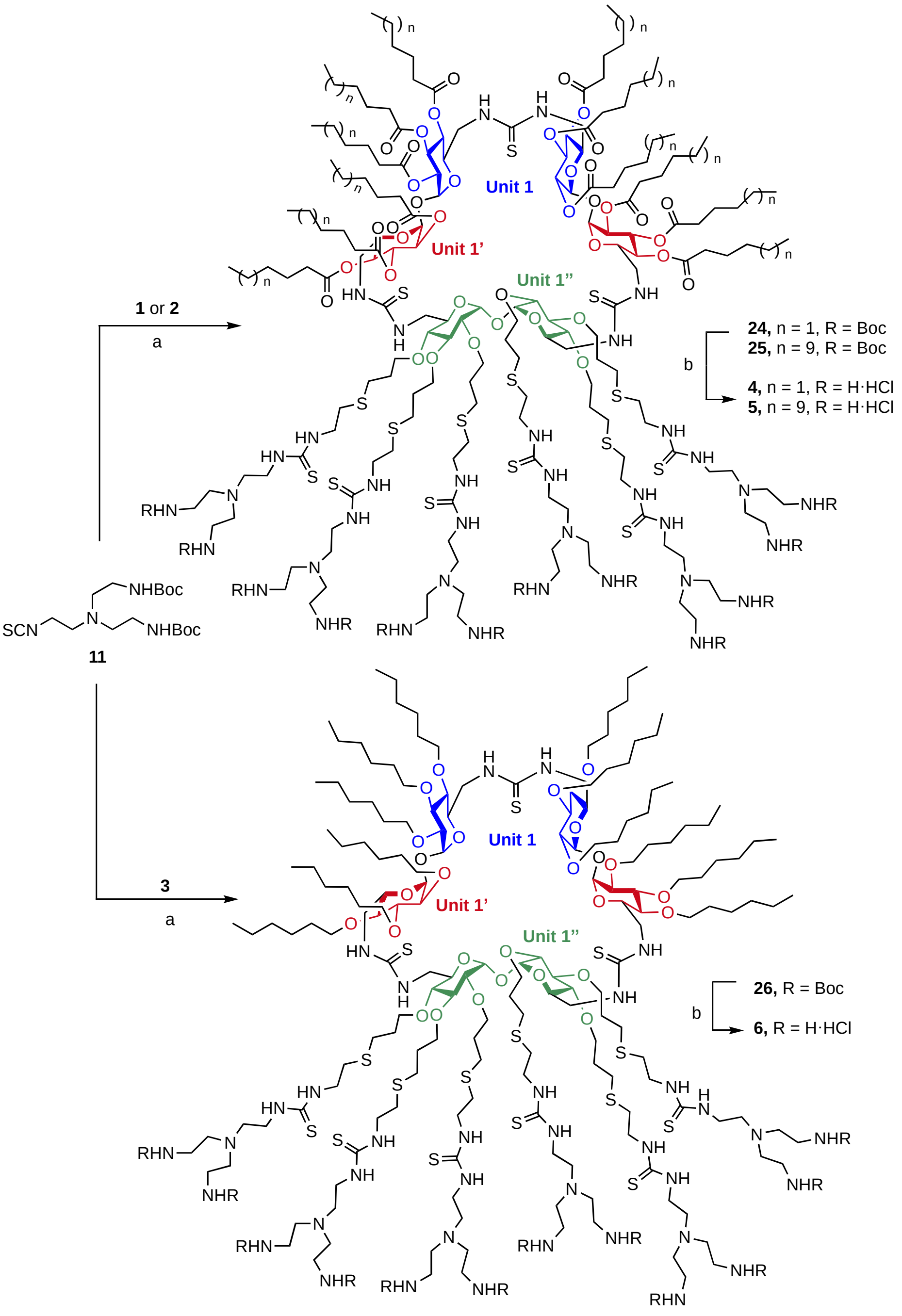

Scheme S4. Synthesis of the dendronized C_1L_2 MM-MNPs **4**-**6**. Reagents and conditions: (a) DMAP, pyridine, 40 ºC, 24 h, 62-75%. (b) 1:1 TFA-DCM, r.t., 1 h, quantitative. Unit 1, unit 1' and unit 1'' refer to spin systems for NMR assignments.

*Hexanoylated Boc-protected C_1L_2 MM-MNP **24**.*

Column chromatography 2:1 EtOAc-cyclohexane→45:5:3 EtOAc-EtOH-H_2O. Yield: 75 mg (71%). R_f = 0.68 (45:5:3 EtOAc-EtOH-H_2O); $[\alpha]_D$ = +38.0 (*c* 1.0, DCM); UV (MeOH): λ_{max} 245 nm (ε_{mM} = 17.1). ^{1}H NMR (500 MHz, DMSO-d_6, 343 K): δ = 6.33 (s, 18 H, NHCS), 5.39 (m, 8 H, H-3, H-3', H-1, H-1'), 5.24 (d, 2 H, H-1''), 5.11-5.08 (dd, 4 H, $J_{1,2}$ = 2.5 Hz, H-2, H-2'), 4.95

(t, 2 H, $J_{4,5}$ = 9.7 Hz, H-4, H-4'), 4.09-4.22 (m, 4 H, H-6"a, H-6"b), 3.80 (m, 4 H, H-5, H-5'), 3.62-3.58 (m, 18 H, H-5", H-6, H-6', CH_2O), 3.43 (m, 14 H, H-3", CH_2N), 3.30 (m, 2 H, H-2"), 3.00 (m, 14 H, H-4", CH_2N), 2.69-2.52 (m, 48 H, SCH_2, NCH_2), 2.53-2.48 (m, 24 H, CH_2N), 2.31-2.19 (m, 24 H, H-2_{Hex}), 1.85-1.70 (m, 12 H, *C*H_2), 1.57-1.49 (m, 24 H, CH_2), 1.41 (3 s, 108 H, C*Me*$_3$), 1.31-1.24 (m, 48 H, CH_2), 0.89-0.85 (m, 36 H, CH_3). ^{13}C NMR (125.7 MHz, DMSO-d_6, 323 K): δ = 183.2 (CS), 172.2, 171.9 (CO ester), 156.1 (CO carbamate), 90.4 (C-1, C-1', C-1"), 81.1, 80.0, 79.7 (C-2", C-3", C-4"), 79.2 (*C*Me_3), 71.6, 70.4 (C-5, C-5"), 70.0, 69.7, 69.4 (C-4, C-4', C-3, C-3', C-2, C-2', CH_2O), 54.39 (CH_2N), 44.3, 44.0, 42.3 (C-6, C-6', C-6", CH_2N), 40.0, 39.8, 39.1 (CH_2N), 33.9, 33.8 (CH_2), 31.2, 31.1, 31.0 (CH_2, CH_2S), 28.7, 28.6 (C*Me*$_3$, SCH_2), 24.4, 24.2, 22.0 (CH_2), 14.0, 13.9 (CH_3). ESI-MS: *m/z* = 5726.1 $[M + Na]^+$. Anal. Calcd for $C_{243}H_{444}N_{36}O_{63}S_{15}$: C, 54.46; H, 8.35; N, 9.41; S, 8.97. Found: C, 54.81; H, 8.63; N, 9.06; S, 8.59.

Myristoylated Boc-protected C_1L_2 MM-MNP ***25***

Column chromatography 2:1 EtOAc-cyclohexane→1:2 acetone-cyclohexane. Yield: 110 mg (75%); R_f = 0.48 (2:3 acetone-cyclohexane); $[\alpha]_D$ = +41.5 (*c* 1.0, DCM); UV (DCM): λ_{max} 248 nm (ε_{mM} = 135.0). ^{1}H NMR (500 MHz, 3:1 $CDCl_3$-CD_3OD, 323 K): δ 5.49 (m, 4 H, H-3, H-3'), 5.44 (bs, 2 H, H-1), 5.33, 5.23 (2 bs, 4 H, H-1', H-1"), 4.89 (bs, 4 H, H-2, H-2'), 4.80 (bd, 4 H, H-4, H-4'), 4.49-3.78 (m, 18 H, H-6a, H-6'a, H-6"a, H-6b, H-6'b, H-6"b, H-5, H-5', H-5"), 3.92-3.82 (m, 12 H, CH_2O), 3.45-3.39 (m, 14 H, H-3", CH_2N), 3.19 (m, 2 H, H-2"), 2.98 (bt, 2 H, H-4''), 2.77 (m, 24 H, CH_2N), 2.69-2.47 (m, 70 H, SCH_2, NCH_2), 2.39-2.25 (m, 24 H, C*H*$_2$CO), 2.03-1.85 (m, 12 H, CH_2), 1.63-1.57 (m, 24 H, CH_2), 1.47-1.44 (m, 108 H, C*Me*$_3$), 1.34-1.28 (bs, 240 H, CH_2), 0.89 (t, 36 H, $^3J_{H,H}$ = 6.5 Hz, CH_3). ^{13}C NMR (125.7 MHz, 3:1 $CDCl_3$-CD_3OD, 323 K): δ = 182.7 (CS), 172.5 (CO ester), 157.0 (CO carbamate), 90.9 (C-1, C-1', C-1"), 79.1 (*C*Me_3), 69.9 (CH_2O), 54.4-53.5 (CH_2S, NCH_2), 44.8, 43.8, 42.1 (CH_2N), 38.7 (CH_2N), 34.0, 33.9 (*C*H_2CO), 29.5, 29.3, 29.2 (CH_2), 27.9, 27.8 (C*Me*$_3$), 24.7, 24.8, 24.6 (CH_2), 22.4 (CH_2), 13.4 (CH_3). ESI-MS: *m/z* = 2278.4 $[M +2 Na]^{3+}$. Anal. Calcd for $C_{340}H_{637}N_{35}O_{63}S_{15}$: C, 60.91; H, 9.58; N, 7.31; S, 7.17. Found: C, 60.58; H, 9.30; N, 6.98; S, 6.83.

Hexylated Boc-protected C_1L_2 CT3 MM-MNP ***26***

Column chromatography (3:1 EtOAc-cyclohexane→45:5:3 EtOAc-EtOH-H_2O). Yield: 75 mg (66%). R_f = 0.78 (45:5:3 EtOAc-EtOH-H_2O); $[\alpha]_D$ = +23.5 (*c* 1.0, DCM); UV (MeOH): λ_{max} 243 nm (ε_{mM} = 14.1). ^{1}H NMR (500 MHz, CD_3OD, 323 K): δ = 5.25 (d, 6 H, H-1, H-1', H-1"), 3.93 (m, 6 H, H-5, H-5',H-5"), 3.86-3.51 (m, 78 H, H-6, H-6', H-6", CH_2O, H-3, H-3', H-3",CH_2N), 3.28 (m, 6 H, H-2, H-2', H-2"), 3.14 (m, 24 H, NCH_2), 3.04 (m, 6 H, H-4, H-4', H-4"), 2.79 (m, 12 H, CH_2), 2.71 (m, 12 H, SCH_2), 2.62 (m, 12 H, CH_2S), 1.94 (m, 12 H, CH_2), 1.61 (m, 24 H, CH_2), 1.47 (m, 108 H, C*Me*$_3$), 1.36 (m, 72 H, CH_2), 0.93 (m, 36 H, CH_3).^{13}C NMR (125.7 MHz,

CD_3OD): δ = 183.5 (CS), 159.8 (CO), 93.5 (C-1), 82.3 (C-3), 81.5 (C-2), 80.9 (C-4), 80.4 (*C*Me3), 74.4 (CH_2O), 71.1 (C-5), 55.7 (CH_2N), 54.8 (NCH_2), 45.1 (CH_2N), 43.3 (CH_2), 39.8 (CH_2N), 33.1 (SCH_2), 29.7 (CH_2), 29.1 (CMe_3), 27.1 (CH_2S), 23.9 (CH_2), 14.6 (CH_3). ESI-MS: *m/z* = 2617.7 $[M + Na]^+$. Anal. Calcd for $C_{243}H_{468}N_{36}O_{51}S_{15}$: C, 56.22; H, 9.09; N, 9.71; S, 9.26. Found: C, 56.33; H, 9.16; N, 9.64; S, 9.10.

Hexanoylated C_1L_2 MM-MNP ***4***.

Compound **24** (75 mg, 0.014 mmol) was treated with 1:1 TFA-DCM (4 mL) at rt for 1 h. Then, the solvent was eliminated under pressure and coevaporated several times with toluene. The residue was dissolved in 0.1 M HCl and freeze-dried (x 2) to give **4** as the corresponding dodecahydrochloride salt. Yield: 55 mg (quantitative). $[\alpha]_D$ = +16.9 (*c* 1.0, DCM); UV (MeOH): λ_{max} 243 nm (ε_{mM} = 54.8). ^{1}H NMR (500 MHz, DMSO-d_6, 343 K): δ = 5.36 (m, 10 H, H-3, H-3', H-3", H-1, H-1', H-1"), 5.23 (bs, 18 H, NHCS), 5.08 (m, 4 H, H-2, H-2'), 4.90 (m, 4 H, H-4, H-4'), 4.26 (dd, 2H, H-6a"), 4.15 (dd, 2H, H-6b"), 3.85 (m, 4 H, H-5, H-5'), 3.80-3.53 (m, 44 H, H-6, H-6', H-1_{Hex}, CH_2O, CH_2N), 3.70 (m, 2 H, H-5"), 3.20 (s, 24 H, CH_2N), 2.83 (s, 24 H, CH_2N), 2.75 (m, 2 H, H-4"), 2.72-2.53 (m, 36 H, CH_2S, NCH_2, SCH_2), 2.27 (m, 24 H, H-2_{Hex}), 1.83 (s, 12 H, CH_2), 1.50 (m, 24 H, H-3_{Hex}), 1.27 (m, 24 H, H-4_{Hex}, H-5_{Hex}), 0.83 (bt, 36 H, H-6_{Hex}). ^{13}C NMR (125.7 MHz, DMSO-d_6, 343 K): δ = 184.7 (CS), 172.8, 172.3 (CO), 91.1 (C-1, C-1', C-1"), 71.38, 70.35 (C-3, C-3', C-3", CH_2O), 69.5, 69.4, 69.2 (C-2, C-2', C-2", C-4, C-4', C-4", C-5, C-5', C-5"), 62.3 (C-6a", C-6b"), 51.6 (CH_2N), 44.4, 44.1 (C-6, C-6'), 37.5 (CH_2N), 34.0, 33.8 (C-2_{Hex}), 31.0, 30.9 (CH_2), 29.3, 28.8 (CH_2S), 27.1 (C-4_{Hex}), 24.8 (SCH_2), 24.2 (C-3_{Hex}), 22.1 (C-5_{Hex}), 13.9 (C-6_{Hex}). Anal. Calcd for $C_{183}H_{348}N_{36}O_{39}S_{15}\cdot12\ HCl\cdot6\ H_2O$: C, 46.73; H, 7.97; N, 10.72; S, 10.22. Found: C, 46.75; H, 7.90; N, 10.47; S, 10.01. MALDI-TOF MS, *m/z* calculated for [M + 5'-AAGCCCGCCCAA-3' (ssDNA)]$^+$: 7728.28. Found: 7728.39. Mass accuracy: 14 ppm.

Myristoylated C_1L_2 MM-MNP 5

Compound **25** (70 mg, 0.010 mmol) was treated with 1:1 TFA-DCM (4 mL) at rt for 1 h. Then, the solvent was eliminated under pressure and coevaporated several times with toluene. The residue was dissolved in 0.1 M HCl and freeze-dried (x 2) to give **5** as the corresponding dodecahydrochloride salt. Yield: 59 mg (99%). R_f = 0.15 (4:1:1 CH_3CN-H_2O-NH_4OH); $[\alpha]_D$ = +18.4 (*c* 1.0, DCM); UV (MeOH): λ_{max} 244 nm (ε_{mM} = 35.4). ^{1}H NMR (500 MHz, 10:1 CD_3OD-D_2O, 323 K): δ = 5.32 (m, 6 H, H-1, H-1', H-1"), 3.58-2.62 (m, 130 H, CH_2O, CH_2N, SCH_2), 2.28 (m, 12 H, CH_2), 2.00 (m, 24 H, CH_2CO), 1.50 (m, 24 H, CH_2), 1.25 (m, 240 H, CH_2), 0.87 (m, 36 H, CH_3). ^{13}C NMR (125.7 MHz, 10:1 CD_3OD-D_2O, 323 K): δ = 46.1 (CH_2N), 31.7 (CH_2CO), 29.1, 25.0, 22.7 (CH_2), 14.1 (CH_3). Anal. Calcd for $C_{279}H_{540}N_{36}O_{39}S_{15}\cdot12\ HCl$: C,

54.39; H, 9.13; N, 8.18; S, 7.81. Found: C, 54.02; H, 8.79; N, 7.80; S, 7.44. MALDI-TOF MS, *m/z* calculated for [M + 5'-AAGCCCGCCCAA-3' (ssDNA)]$^+$: 9089.91. Found: 9089.98. Mass accuracy: 8 ppm.

Hexylated C_1L_2 MM-MNP ***6***

Compound **26** (75 mg, 0.014 mmol) was treated with 1:1 TFA-DCM (4 mL) at rt for 1 h. Then, the solvent was eliminated under pressure and coevaporated several times with toluene. The residue was dissolved in 0.1 M HCl and freeze-dried (x 2) to give **6** as the corresponding dodecahydrochloride salt. Yield: 64 mg (quantitative); $[\alpha]_D$ = +30.8 (*c* 1.0, MeOH); UV (MeOH): λ_{max} 250 nm (ε_{mM} = 3.9). ^{1}H NMR (500 MHz, CD_3OD): 5.26 (m, 6 H, H-1, H-1', H-1''), 3.87-3.45 (m, 48 H, CH_2N, H-5, H-5', H-5'', H-6, H-6', H-6'', CH_2O, H-3, H-3', H-3''), 3.12 (m, 6 H, H-2, H-2', H-2''), 2.93 (m, 6 H, H-4, H-4', H-4''), 2.82 (m, 12 H, CH_2N), 2.69 (m, 12 H, SCH_2), 2.67 (m, 12 H, CH_2S), 1.83 (m, 12 H, CH_2), 1.49 (m, 24 H, CH_2), 1.23 (m, 72 H, CH_2), 0.83 (m, 36 H, CH_3). ^{13}C NMR (125.7 MHz, CD_3OD): δ = 93.1 (C-1), 82.3 (C-3), 81.5 (C-2), 74.5 (C-4), 72.9 (CH_2O), 71.3 (C-5), 53.5 (CH_2N), 52.4 (NCH_2), 45.1 (CH_2N), 42.1 (CH_2), 38.2 (CH_2N), 33.1 (SCH_2), 31.8, 29.5 (CH_2), 27.2 (CH_2S), 24.0 (CH_2), 15.0 (CH_3). Anal. Calcd for $C_{183}H_{372}N_{36}O_{27}S_{15}\cdot 12$ HCl: C, 49.64; H, 8.74; N, 11.39; S, 10.86. Found: C, 49.29; H, 8.80; N, 11.11; S, 10.52. MALDI-TOF MS, *m/z* calculated for [M + 5'-AAGCCCGCCCAA-3' (ssDNA)]$^+$: 7575.52. Found: 7575.41. Mass accuracy: 15 ppm.

Synthesis of the C_2L_1 MM-MNPs 7-10

Preparation of the unbranched C_2L_1 MM-MNPs ***7*** *and* ***8***

Compounds **7** and **8** were obtained by macrocyclization reactions of the corresponding α,α'-trehalose diamine derivative **28** or **29** and the tetrasaccharidic diisothiocyanate derivative **30** as follows: to a solution of the corresponding diamine **28** or **29** (0.045 mmol) in pyridine (4 mL), a solution of **30** (0.045 mmol) and DMAP (17 mg, 0.135 mmol) in pyridine (4 mL) was slowly added and the mixture was stirred at 40 ºC overnight. The mixture was concentrated, and the resulting residue purified by column chromatography using the solvent indicated in each case to afford the Boc-protected patchy CT3 adduct **31** or **32**. The carbamate protecting groups were removed by treatment with 1:1 TFA-DCM at room temperature, quantitatively yielding the unbranched MM-MNPs **7** and **8** (Scheme S5)

The synthesis of the diamine precursors **28** and **29** was accomplished in two steps from diazide **16**[4] by acylation of the secondary hydroxyls by reaction with hexyl or tetradecyl bromide, to give **17** (see Scheme S2) and **27** (Scheme S5) respectively, followed by reduction of the azido groups as described hereinafter.

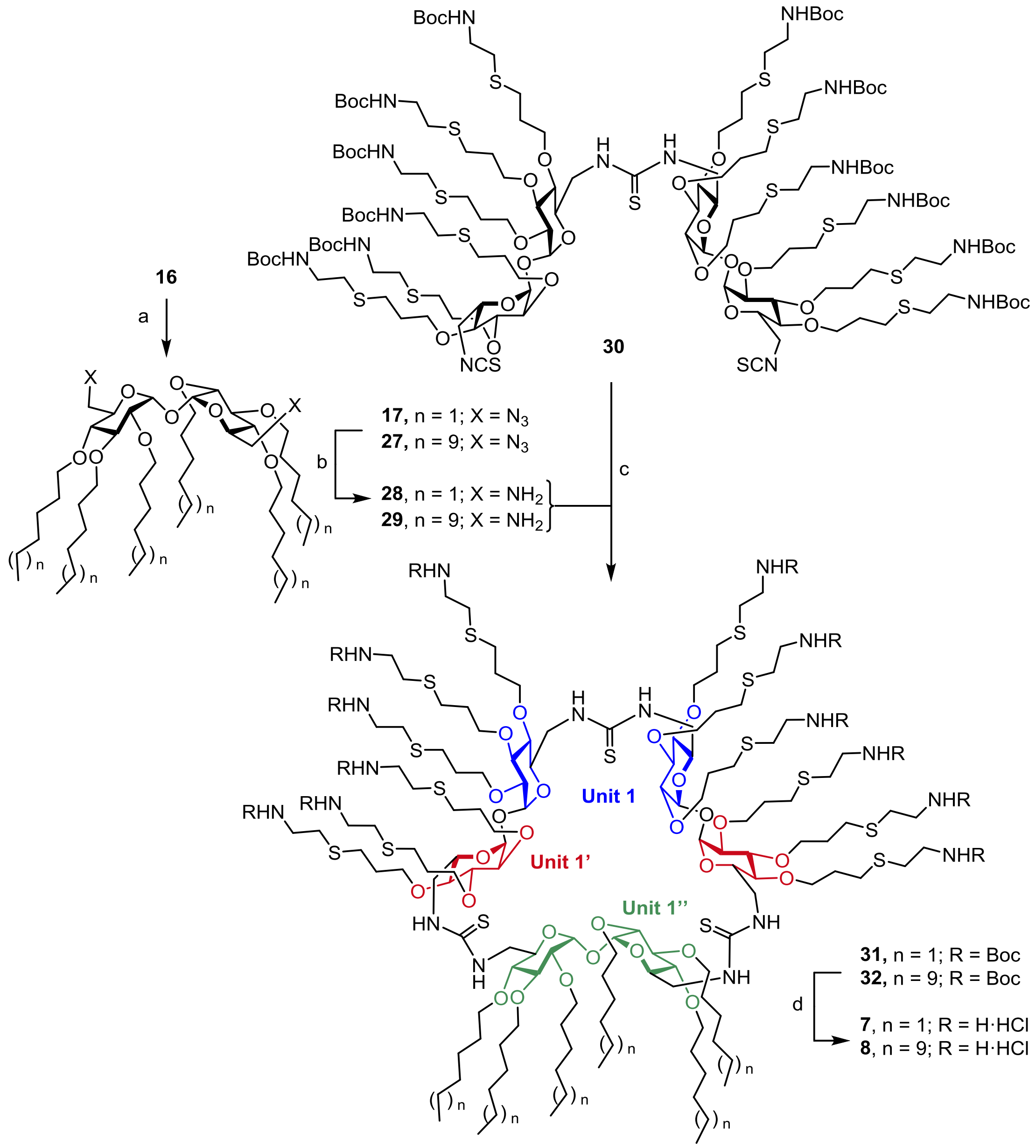

Scheme S5. Synthesis of the unbranched C_2L_1 MM-MNPs **7** and **8**. Reagents and conditions: (a) 1-bromohexane or 1-bromotetradecane, NaH, DMF, 60 ºC, 24 h, 75-87%. (b) TPP, THF, NH_4OH, 50 ºC, 24 h, 58-59%. (c) DMAP, pyridine, 40 ºC, 24 h, 40-52%. (d) 1:1 TFA-DCM, r.t., 1 h, quantitative. (c) **11**, DMAP, pyridine, 40 ºC, 24 h, 71-99%. Unit 1, unit 1' and unit 1'' refers to spin systems for NMR assigments.

*6,6'-Diazido-6,6'-dideoxy-2,3,4,2',3',4'-hexa-O-tetradecyl-α,α'-trehalose (**27**)*

To a solution of **16** (399 mg, 1.01 mmol) in dry DMF (24 mL), NaH (439 mg, 18.3 mmol) was added and the mixture was stirred at 0 ºC for 10 min. 1-Bromotetradecane (5.45 mL, 18.3 mmol) was dropwise added, under Ar atmosphere, and the mixture was stirred for 24 h at 60 ºC. Solvents were removed and the resulting residue was suspended in DCM (50 mL). The suspension was washed with H_2O (3 x 15 mL) and the organic layer was dried ($MgSO_4$), filtered, concentrated

and purified by column chromatography (1:100 EtOAc-cyclohexane). Yield: 193 mg (87%); R_f = 0.26 (1:40 EtOAc-cyclohexane); $[\alpha]_D$ = +68.0 (*c* 1.0, DCM). ^{1}H NMR (300 MHz, $CDCl_3$): δ = 5.11 (d, 2 H, $J_{1,2}$ = 3.7 Hz, H-1), 4.04 (ddd, 2 H, $J_{4,5}$ = 9.7 Hz, $J_{5,6a}$ = 4.4 Hz, $J_{5,6b}$ = 2.9 Hz, H-5), 3.81 (m, 4 H, CH_2O), 3.70-3.47 (m, 10 H, CH_2O, H-3), 3.40 (m, 4 H, H-6), 3.25 (dd, 2 H, $J_{2,3}$ = 9.7 Hz, H-2), 3.16 (t, 2 H, $J_{3,4}$ = 9.7 Hz, H-4), 1.60-1.48 (m, 12 H, CH_2), 1.26 (bs, 132 H, CH_2), 0.88 (t, 18 H, $^3J_{H,H}$ = 6.7 Hz, CH_3). ^{13}C NMR (75.5 MHz, $CDCl_3$): δ = 93.5 (C-1), 81.0 (C-3), 80.3 (C-2), 78.7 (C-4), 73.6, 73.4, 71.7 (CH_2O), 70.5 (C-5), 51.4 (C-6), 31.9, 30.7, 30.4, 30.2, 29.7, 29.6, 29.4, 26.3, 26.2, 22.7 (CH_2), 14.1 (CH_3). ESI-MS: *m/z* = 1592.4 $[M + Na]^+$. Anal. Calcd for $C_{96}H_{188}N_6O_9$: C, 73.42; H, 12.07; N, 5.35. Found: C, 73.61; H, 12.12; N, 5.27.

*6,6'-Diamino-6,6'-dideoxy-2,3,4,2',3',4'-hexa-O-hexyl-α,α'-trehalose dihydrochloride (**28**)*

To a solution of **17** (250 mg, 0.278 mmol) in THF (22.5 mL), TPP (293 mg, 0.39 mmol) was added and the mixture was stirred at rt for 15 min. Then NH_4OH (2.5 mL) was added and the solution was stirred 24 h at 50 ºC. The mixture was concentrated and the resulting residue purified by column chromatography (EtOAc→45:5:3 AcOEt-EtOH-H_2O→5:1 DCM-MeOH) and freeze-drying from a 1 M HCl solution, affording **28**. Yield: 137 mg (58%). R_f = 0.40 (5:1 DCM-MeOH); $[\alpha]_D$ = +95.0 (*c* 1.1, DCM).^{1}H NMR (300 MHz, $CDCl_3$): δ = 5.06 (d, 2 H, $J_{1,2}$ = 3.5 Hz, H-1), 3.79 (m, 6 H, CH_2O, H-5), 3.71-3.46 (m, 8 H, CH_2O), 3.59 (t, 2 H, $J_{2,3}$ = $J_{3,4}$ = 9.5 Hz, H-3), 3.19 (dd, 2 H, H-2), 3.09 (t, 2 H, $J_{4,5}$ = 9.5 Hz, H-4), 2.93 (dd, 2 H, $J_{6a,6b}$ = 13.5 Hz, $J_{5,6a}$ = 2.4 Hz, H-6a), 2.77 (dd, 2 H, $J_{5,6b}$ = 5.5 Hz, H-6b), 1.59-1.49 (m, 12 H, CH_2), 1.34-1.27 (m, 36 H, CH_2), 0.90-0.84 (m, 18 H, CH_3). ^{13}C NMR (75.5 MHz, $CDCl_3$): δ= 93.0 (C-1), 81.2 (C-3), 80.5 (C-2), 79.2 (C-4), 73.4, 73.2 (CH_2O), 71.8 (C-5), 71.6 (CH_2O), 42.7 (C-6), 31.8, 30.4, 30.6, 30.2, 25.9, 25.8, 22.6 (CH_2), 14.0 (CH_3). ESI-MS: *m/z* = 845.6 $[M + H]^+$. Anal. Calcd for $C_{48}H_{98}Cl_2N_2O_9$: C, 62.79; H, 10.76; N, 3.05. Found: C, 62.43; H, 10.41; N, 2.87.

*6,6'-Diamino-6,6'-dideoxy-2,3,4,2',3',4'-hexa-O-tetradecyl-α,α'-trehalose dihydrochloride (**29**).*

To a solution of **27** (447 mg, 0.285 mmol) in THF (23 mL), TPP (105 mg, 0.40 mmol) was added and the mixture was stirred at rt for 15 min. Then NH_4OH (2.6 mL) was added and the solution was stirred 16 h at 50 ºC. The mixture was concentrated, the resulting residue purified by column chromatography (EtOAc→45:5:3 AcOEt-EtOH-H_2O→5:1 DCM-MeOH) and freeze-drying from a 1 M HCl solution affording **25**. Yield: 137 mg (59%); R_f = 0.47 (9:1 DCM-MeOH); $[\alpha]_D$ = +24.4 (*c* 1.0, DCM). ^{1}H NMR (300 MHz, 10:1 $CDCl_3$-CD_3OD): δ = 4.97 (d, 2 H, $J_{1,2}$ = 3.5 Hz, H-1), 3.70 (m, 6 H, CH_2O, H-5), 3.61-3.37 (m, 8 H, CH_2O), 3.47 (t, 2 H, $J_{2,3}$ = $J_{3,4}$ = 9.4 Hz, H-3), 3.12 (dd, 2 H, H-2), 2.95 (t, 2 H, $J_{4,5}$ = 9.4 Hz, H-4), 2.88 (dd, 2 H, $J_{6a,6b}$ = 13.8 Hz, $J_{5,6a}$ = 3.3 Hz, H-6a), 2.73 (dd, 2 H, $J_{5,6b}$ = 5.6 Hz, H-6b), 1.49-1.37 (m, 12 H, CH_2), 1.15 (bs, 132 H, CH_2),

0.77, 0.76 (2 t, 18 H, $^3J_{H,H}$ = 6.7 Hz, CH_3). ^{13}C NMR (75.5 MHz, 10:1 $CDCl_3$-CD_3OD): δ = 92.7 (C-1), 80.9 (C-3), 79.9 (C-2), 78.8 (C-4), 73.4, 73.1, 71.6 (CH_2O), 69.8 (C-5), 41.2 (C-6), 31.8-22.5 (CH_2), 13.9 (CH_3). ESI-MS: *m/z* = 1519.4 $[M + 2 H]^{2+}$, 1541.4 $[M + H + Na]^{2+}$. Anal. Calcd for $C_{96}H_{194}Cl_2N_2O_9$: C, 72.45; H, 12.29; N, 1.76. Found: C, 72.13; H, 11.95; N, 1.51.

*Hexylated Boc-protected C_2L_1 MM-MNP **31***

Compound **31** was obtained from **30** and **28** following the general procedure above described. Column chromatography (1.5:4 acetone-toluene). Yield: 77 mg (40%). R_f = 0.60 (1:2 acetone-toluene); $[\alpha]_D$ = +58.0 (*c* 1.0, DCM); UV (MeOH): λ_{max} 245 nm (ε_{mM} = 36.2^{1}H NMR (500 MHz, $CDCl_3$, 333 K): δ = 6.57, 6.20, 5.75 (bs, 6 H, NHCS), 5.03 (m, 6 H, H-1, H-1', H-1"), 4.14 (m, 6 H, H-6a, H-6'a, H-6"a), 3.97-3.87 (m, 6 H, H-5, H-5', H-5"), 3.98-3.62 (m, 12 H, CH_2O), 3.81-3.58 (m, 6 H, H-6b, H-6'b, H-6"b), 3.61-3.58 (m, 12 H, H-1_{Hex}), 3.32-3.29 (m, 6 H, H-3, H-3', H-3"), 3.25 (m, 24 H, CH_2N), 3.23-3.20 (m, 6 H, H-2, H-2', H-2"), 3.11-3.04 (m, 6 H, H-4, H-4', H-4"), 2.67-2.6 (m, 48 H, CH_2S), 1.9-1.81 (m, 12 H, CH_2), 1.59-1.53(m, 12 H, CH_2), 1,44 (s, 108 H, C*Me*$_3$), 1.41-1.24 (m, 36 H, CH_2), 0.94-0.89 (m, 18 H, CH_3). ^{13}C NMR (125.7 MHz, $CDCl_3$, 333 K): δ = 184.1 (CS), 155.8, 155.7 (CO), 92.9, 92.6 (C-1, C-1', C-1"), 81.1, 81.0 (C-3, C-3', C-3"), 80.3, 80.2 (C-2, C-2', C-2"), 79.5 (C-4, C-4', C-4"), 73.5, 73.3 (CH_2O), 71.9, 71.7, 71.5 (C-5, C-5', C-5"), 70.2, 69.9 (C-1_{Hex}), 45.6, 45.3 (C-6, C-6', C-6"), 40.2 (CH_2N), 32.4, 31.7 (SCH_2), 30.7, 30.5, 30.4, 30.3 (CH_2S, CH_2), 29.6 (*C*H_2CH_2O), 28.7 (C*Me*$_3$), 26.9, 22.5 (CH_2), 13.9, 13.8, 13.8 (CH_3). ESI-MS: *m/z* = 2153.9 $[M + 2 Na]^{2+}$. Anal. Calcd for $C_{195}H_{366}N_{18}O_{51}S_{15}$: C, 54.98; H, 8.66; N, 5.92; S, 11.29. Found: C, 55.13; H, 8.51; N, 5.75; S, 10.97.

Tetradecylated Boc-protected MNP 32.

Compound **32** was obtained from **30** and **29** (0.045 mmol) following the general procedure above described. Column chromatography (1:1 EtOAc-cyclohexane and 100:10:1 DCM-MeOH-H_2O). Yield: 183 mg (52%). R_f = 0.50 (100:10:1 DCM-MeOH-H_2O); $[\alpha]_D$ = +31.4 (*c* 1.0, DCM); UV (MeOH): λ_{max} 244 nm (ε_{mM} = 80.6). ^{1}H NMR (400 MHz, $CDCl_3$, 323K): δ = 6.34, 6.24 (2 bs, 6 H, NHCS), 5.05 (m, 6 H, H-1, H-1', H-1"), 3.99 (m, 6 H, H-6a, H-6'a, H-6"a), 3.91-3.85 (m, 6 H, H-5, H-5', H-5"), 3.84-3.79 (m, 36 H, CH_2O, H-1_{Myr}), 3.77-3.51 (m, 6 H, H-6b, H-6'b, H-6"b), 3.64-3.59 (m, 6 H, H-3, H-3', H-3"), 3.36 (m, 6 H, H-2, H-2', H-2"), 3.33 (m, 24 H, CH_2N), 3.26-3.20 (m, 6 H, H-4, H-4', H-4"), 3.05-2.70 (m, 24 H, SC*H*$_2CH_2N$), 2.69-2.64 (m, 24 H, CH_2S), 1.93-1.85 (m, 24 H, CH_2), 1.55 (s, 108 H, C*Me*$_3$), 1.32 (m, 144 H, CH_2), 0.92 (m, 18 H, CH_3). ^{13}C NMR (100.6 MHz, $CDCl_3$, 323 K): δ = 155.7 (CO), 92.7 (C-1, C-1', C-1"), 81.0 (C-3, C-3', C-3"), 80.3 (C-2, C-2', C-2"), 80.2 (C-4, C-4', C-4"), 79.4 (*C*Me_3) 73.5 (CH_2O), 71.7 (C-5, C-5', C-5"), 70.0 (C-1_{Myr}), 40.1 (C-6, C-6', C-6"), 38.8 (CH_2N), 32.3 (SCH_2), 30.7 (CH_2S), 29.7 (CH_2), 28.4 (C*Me*$_3$), 26.3, 26.2, 23.8, 22.9, 22.6 (CH_2), 13.96 (CH_3). ESI-MS: *m/z* = 2489.5 $[M + 2 Na]^{2+}$.

Anal. Calcd for $C_{243}H_{462}N_{18}O_{51}S_{15}$: C, 59.16; H, 9.44; N, 5.11; S, 9.75. Found: C, 58.83; H, 9.24; N, 4.98; S, 9.53.

*Hexylated C_2L_1 MM-NMP **7***

Compound **31** (74 mg, 0.017 mmol) was treated with 1:1 TFA-DCM (4 mL) at rt for 1 h. Then, the solvent was eliminated under pressure and coevaporated several times with toluene. The residue was dissolved in 0.1 M HCl and freeze-dried (x 2) to yield the product as the corresponding dodecachloride salt. Yield: 60 mg (quantitative). $[\alpha]_D$ = +47.1 (*c* 1.0, MeOH); UV (MeOH): λ_{max} 242 nm (ε_{mM} = 36.5). ^{1}H NMR (500 MHz, CD_3OD, 323 K): δ = 5.35 (m, 6 H, H-1, H-1', H-1"), 3.95-3.90 (m, 6 H, H-5, H-5', H-5"), 3.90-3.60 (m, 42 H, H-6, H-6', H-6", CH_2O), 3.64-3.6 (m, 6 H, H-3, H-3', H-3"), 3.31 (m, 6 H, H-2, H-2', H-2"), 3.21 (m, 24 H, CH_2N), 3.05-3.01 (m, 6 H, H-4, H-4', H-4"), 2.91-2.86 (m, 24 H, SCH_2), 2.79-2.68 (m, 24 H, CH_2S), 1.96-1.86 (m, 24 H, CH_2), 1.63-1.55 (m, 12 H, CH_2), 1.45-1.31 (m, 36 H, CH_2), 0.95-0.90 (m, 18 H, CH_3). ^{13}C NMR (100.6 MHz, CD_3OD, 323 K): δ = 183.4 (CS), 91.4, 91.1 (C-1, C-1', C-1"), 80.9 (C-3, C-3', C-3"), 80.5 (C-2, C-2', C-2"), 80.1, 79.8 (C-4, C-4', C-4"), 73.0, 72.4, 71.3, 71.1 (C-5, C-5', C-5", CH_2O), 70.0, 69.5 (CH_2O), 38.8 (CH_2N), 31.7, 31.5 (CH_2), 30.2 (SCH_2), 29.2 (CH_2), 28.5, 28.4, 28.2 (CH_2S), 25.6 (CH_2), 22.34, 22.3 (CH_2), 13.1, 13.01, 12.9 (CH_3). ESI-MS: *m/z* = 3059.0 $[M + H]^+$, 1529.9 $[M + 2 H]^{2+}$. Anal. Calcd for $C_{135}H_{270}N_{18}O_{27}S_{15}\cdot 12$ HCl: C, 46.38; H, 8.13; N, 7.21; S, 13.76. Found: C, 46.02; H, 7.73; N, 6.86; S, 13.39. MALDI-TOF MS, *m/z* calculated for [M + 5'-AAGCCCGCCCAA-3' (ssDNA)]$^+$: 6842.34.28. Found: 6842.50. Mass accuracy: 7 ppm.

*Tetradecylated C_2L_1 MM-NMP **8**.*

Compound **32** (70 mg, 0.016 mmol) was treated with 1:1 TFA-DCM (4 mL) at rt for 1 h. Then, the solvent was eliminated under pressure and coevaporated several times with toluene. The residue was dissolved in 0.1 M HCl and freeze-dried (x 2) to yield the product as the corresponding dodecachloride salt. Yield: 47 mg (84%). $[\alpha]_D$ = +17.2 (*c* 1.0, MeOH); UV (MeOH): λ_{max} 241 nm (ε_{mM} = 41.1). ^{1}H NMR (400 MHz, 5:1 CD_3OD-D_2O, 323 K): δ = 5.40-5.37 (m, 6 H, H-1, H-1', H-1"), 3.92-3.86 (m, 6 H, H-5, H-5', H-5"), 3.84-3.74 (m, 48 H, H-6, H-6', H-6", CH_2O, H-1_{Hex}), 3.59-3.51 (m, 6 H, H-3, H-3', H-3"), 3.31 (m, 6 H, H-2, H-2', H-2"), 3.21 (m, 24 H, CH_2N), 3.05-3.01 (m, 6 H, H-4, H-4', H-4"), 2.93-2.88 (m, 24 H, SCH_2), 2.78-2.69 (m, 24 H, CH_2S), 1.97-1.89 (m, 24 H, CH_2), 1.44-1.42 (m, 12 H, CH_2), 1.32 (s, 132 H, CH_2), 0.94 (t, 18 H, CH_3). ^{13}C NMR (100.6 MHz, 5:1 CD_3OD-D_2O, 323 K): δ = 91.0 (C-1, C-1', C-1"), 80.7 (C-3, C-3', C-3"), 79.9 (C-2, C-2', C-2"), 79.8 (C-4, C-4', C-4"), 71.5, 71.4 (C-5, C-5', C-5", CH_2O), 69.7 (C-1_{Myr}), 38.8 (CH_2N), 31.8 (C-2_{Myr}), 30.2 (SCH_2), 30.0 (CH_2), 29.6 (CH_2S), 29.5, 29.3, 28.9, 28.4, 27.9, 26.5, 26.4, 26.3, 26.0, 22.5 (CH_2), 13.5 (CH_3). ESI-MS: *m/z* = 1244.79 [M

+ 3 H]$^{3+}$. Anal. Calcd for $C_{183}H_{366}N_{18}O_{27}S_{15}$·12 HCl: C, 52.72; H, 9.14; N, 6.05; S, 11.53. Found: C, 52.40; H, 8.87; N, 5.69; S, 11.16.

*Preparation of the dendronized C_2L_1 MM-MNPs **9** and **10***

To a solution of **7** or **8** (0.045 mmol) in pyridine (4 mL), a solution of **11** (0.045 mmol) and DMAP (17 mg, 0.135 mmol) in pyridine (4 mL) was slowly added and the mixture was stirred at 40 ºC overnight. Column chromatography with the solvent indicated in each case afforded the dendronized Boc-protected CT3 patchy macrocycles **33** and **34**, respectively. Trifluoroacetic acid (TFA)-promoted hydrolysis of the carbamate groups and final lyophilization from 0.1 M HCl (x 2) provided the target cationic Mickey Mouse molecular nanoparticles **4-6** in quantitative yield (Scheme 6).

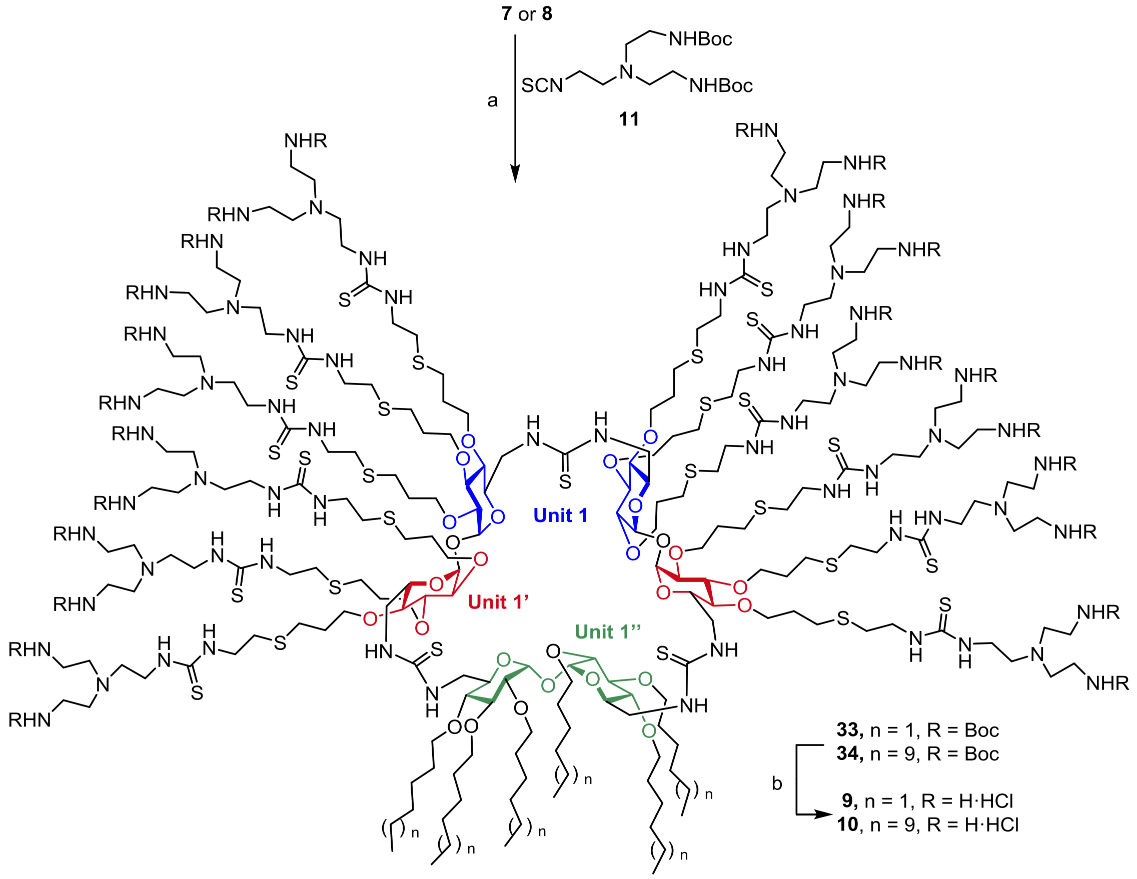

Scheme S6. Synthesis of the dendronized C_2L_1 MM-MNPs **9** and **10**. Reagents and conditions: (a) **11**, DMAP, pyridine, 40 ºC, 24 h, 71-99%. (b) 1:1 TFA-DCM, r.t., 1 h, quantitative. Unit 1, unit 1' and unit 1'' refers to spin systems for NMR assignments.

*Hexylated Boc-protected C_2L_1 MM-MNP **33***

Column chromatography 3:1 EtOAc-cyclohexane→45:5:3 EtOAc-EtOH-H_2O. Yield: 55 mg (71%). R_f = 0.70 (45:5:3 EtOAc-EtOH-H_2O); $[\alpha]_D$ = +19.4 (*c* 1.0, MeOH); UV (MeOH): λ_{max} 243 nm (ε_{mM} = 201.5). ^{1}H NMR (400 MHz, $CDCl_3$, 333 K): δ = 5.11 (m, 6 H, H-1, H-1', H-1"), 3.96-3.68 (m, 60 H, CH_2O, NCH_2), 3.59 (m, 48 H, CH_2N), 3.18 (m, 24 H, NCH_2), 2.80 (m, 24 H, SCH_2), 2.70 (m, 24 H, SCH_2), 2.61 (m, 24 H, CH_2S), 1.95 (m, 24 H, CH_2), 1.61 (m, 12 H, CH_2), 1.48 (m, 216 H, C*Me*$_3$), 1.35 (m, 36 H, CH_2), 0.93 (s, 18 H, CH_3). ^{13}C NMR (100.6 MHz, $CDCl_3$): δ = 182.3 (CS), 157.1 (CO), 79.8 (*C*Me$_3$), 55.4 (NCH_2), 53.9 (CH_2N), 43.9 (*C*H$_2$NHCS), 39.1 (*C*H$_2$NHBoc), 31.4 (CH_2), 30.4 (SCH_2), 29.8 (CH_2S), 28.5 (C*Me*$_3$), 25.8 (CH_2), 14.2 (CH_3). ESI-MS: *m/z* = 2595.8 [M + 3 Na]$^{3+}$. Anal. Calcd for $C_{339}H_{654}N_{66}O_{75}S_{27}$: C, 52.74; H, 8.54; N, 11.97; S, 11.21. Found: C, 52.49; H, 8.37; N, 11.81; S, 10.94.

Tetradecylated Boc-protected C_2L_1 CT3 MM-MNP ***34***

Column chromatography 3:1 EtOAc-cyclohexane→45:5:3 EtOAc-EtOH-H_2O). Yield: 116 mg (99%). R_f = 0.55 (45:5:3 EtOAc-EtOH-H_2O); $[\alpha]_D$ = +17.3 (*c* 1.0, DCM); UV (MeOH): λ_{max} 243 nm (ε_{mM} = 275.1). ^{1}H NMR (500 MHz, CD_3OD, 333 K): δ = 5.19 (m, 6 H, H-1, H-1', H-1"), 3.97-3.83 (m, 36 H, CH_2O), 3.75 (m, 24 H, NCH_2), 3.54 (m, 48 H, CH_2N), 3.15 (m, 24 H, NCH_2), 3.09 (m, 6 H, H-2, H-2', H-2"), 2.98 (m, 6 H, H-4, H-4', H-4"), 2.80 (m, 24 H, CH_2), 2.72 (m, 24 H, SCH_2), 2.62 (m, 24 H, CH_2S), 1.95 (m, 24 H, CH_2), 1.60 (m, 12 H, CH_2), 1.47 (m, 216 H, C*Me*$_3$), 1.32 (bs, 132 H, CH_2), 0.91 (t, 18 H, $^3J_{H,H}$ = 6.5 Hz, CH_3). ^{13}C NMR (125.7 MHz, CD_3OD, 323 K): δ = 182.0 (CS), 158.3 (CO), 91.3 (C-1, 1', 1"), 81.2 (C-3, C-2, C-4), 80.2 (*C*Me$_3$), 71.8 (CH_2O), 70.5 (C-5, 5', 5"), 54.4 (NCH_2), 54.1 (CH_2N), 43.9 (CH_2N), 35.8 (CH_2N), 31.6 (CH_2), 30.6 (SCH_2), 29.4 (CH_2S), 28.4 (CH_2), 27.5 (C*Me*$_3$), 22.3 (CH_2), 13.1 (CH_3). ESI-MS: *m/z* = 1679.6 [M + 5 H]$^{5+}$, 2099.2 [M + 4 H]$^{4+}$. Anal. Calcd for $C_{387}H_{750}N_{66}O_{75}S_{27}$: C, 55.37; H, 9.01; N, 11.01; S, 10.31. Found: C, 55.18; H, 8.79; N, 10.71; S, 9.97.

Heexylated C_2L_1 MM-MNP ***9***

Compound **33** (55 mg, 0.010 mmol) was treated with 1:1 TFA-DCM (3 mL) at rt for 1 h. Then, the solvent was eliminated under pressure and coevaporated several times with toluene. The residue was dissolved in 0.1 M HCl and freeze-dried (x 2) to yield **9** as the corresponding tetraicosahydrochloride salt. Yield: 38 mg (quantitative); $[\alpha]_D$ = +11.4 (*c* 0.8, MeOH); UV (DCM): λ_{max} 242 nm (ε_{mM} = 142.0). ^{1}H NMR (500 MHz, CD_3OD): δ = 3.87-3.41 (m, 108 H, CH_2O, CH_2N), 3.05 (m, 24 H, NCH_2), 2.80 (m, 24 H, CH_2), 2.67 (m, 24 H, SCH_2), 2.65 (m, 24 H, CH_2S), 1.82 (m, 24 H, CH_2), 1.51 (m, 12 H, CH_2), 1.24 (m, 36 H, CH_2), 0.83 (s, 18 H, CH_3). ^{13}C NMR (125.7 MHz, CD_3OD): δ = 52.7 (NCH_2), 52.2 (CH_2N), 42.5 (CH_2N), 38.7 (CH_2N), 31.7 (SCH_2) 30.7 (CH_2), 29.7 (CH_2S), 27.2, 23.8 (CH_2), 14.5 (CH_3). Anal. Calcd for $C_{219}H_{462}N_{66}O_{27}S_{27}\cdot24HCl$: C, 42.47; H, 7.91; N, 14.93; S, 13.98. Found: 42.52; H, 7.59; N, 14.67;

S, 13.60. MALDI-TOF MS, *m/z* calculated for [M + 5’-AAGCCCGCCCAA-3’ (ssDNA)]$^{+}$: 8887.54. Found: 8887.58. Mass accuracy: 4 ppm.

Tetradecylated C_2L_1 MM-MNP ***10***

Compound **34** (71 mg, 0.010 mmol) was treated with 1:1 TFA-DCM (4 mL) at rt for 1 h. Then, the solvent was eliminated under pressure and coevaporated several times with toluene. The residue was dissolved in 0.1 M HCl and freeze-dried (x 2) to yield the product as the corresponding tetraicosahydrochloride salt. Yield: 61 mg (quantitative); $[\alpha]_D$ = +16.8 (*c* 1.0, MeOH); UV (DCM): λ_{max} 242 nm (ε_{mM} = 125.9). ^{1}H NMR (500 MHz, CD_3OD, 323 K): δ = 5.34 (m, 6 H, H-1, H-1’, H-1”), 3.94 (m, 36 H, CH_2O), 3.85 (m, 24 H, NCH_2), 3.74 (m, 48 H, NCH_2), 3.69 (m, 24 H, CH_2N), 3.15 (m, 24 H, CH_2), 2.90 (m, 24 H, SCH_2), 2.80 (m, 24 H, CH_2S), 1.94 (m, 24 H, CH_2), 1.62 (m, 12 H, CH_2), 1.33 (bs, 132 H, CH_2), 0.92 (t, 18 H, $^{3}J_{H,H}$ = 6.5 Hz, CH_3).^{13}C NMR (125.7 MHz, D_2O): δ = 51.9 (NCH_2), 50.0 (CH_2N), 44.7, 36.5 (CH_2NH), 32.1 (SCH_2), 30.0 (CH_2), 29.6 (CH_2S), 28.1, 26.6, 22.7 (CH_2), 14.1 (CH_3). Anal. Calcd for $C_{267}H_{558}N_{66}O_{27}S_{27}$·24 HCl·6 H_2O: C, 45.98; H, 8.58; N, 13.25; S, 12.41. Found: 46.03; H, 8.64; N, 13.18; S, 12.33. MALDI-TOF MS, *m/z* calculated for [M + 5’-AAGCCCGCCCAA-3’ (ssDNA)]$^{+}$: 9581.04. Found: 9581.15. Mass accuracy: 12 ppm.

^{1}H and ^{13}C NMR spectra of the new compounds

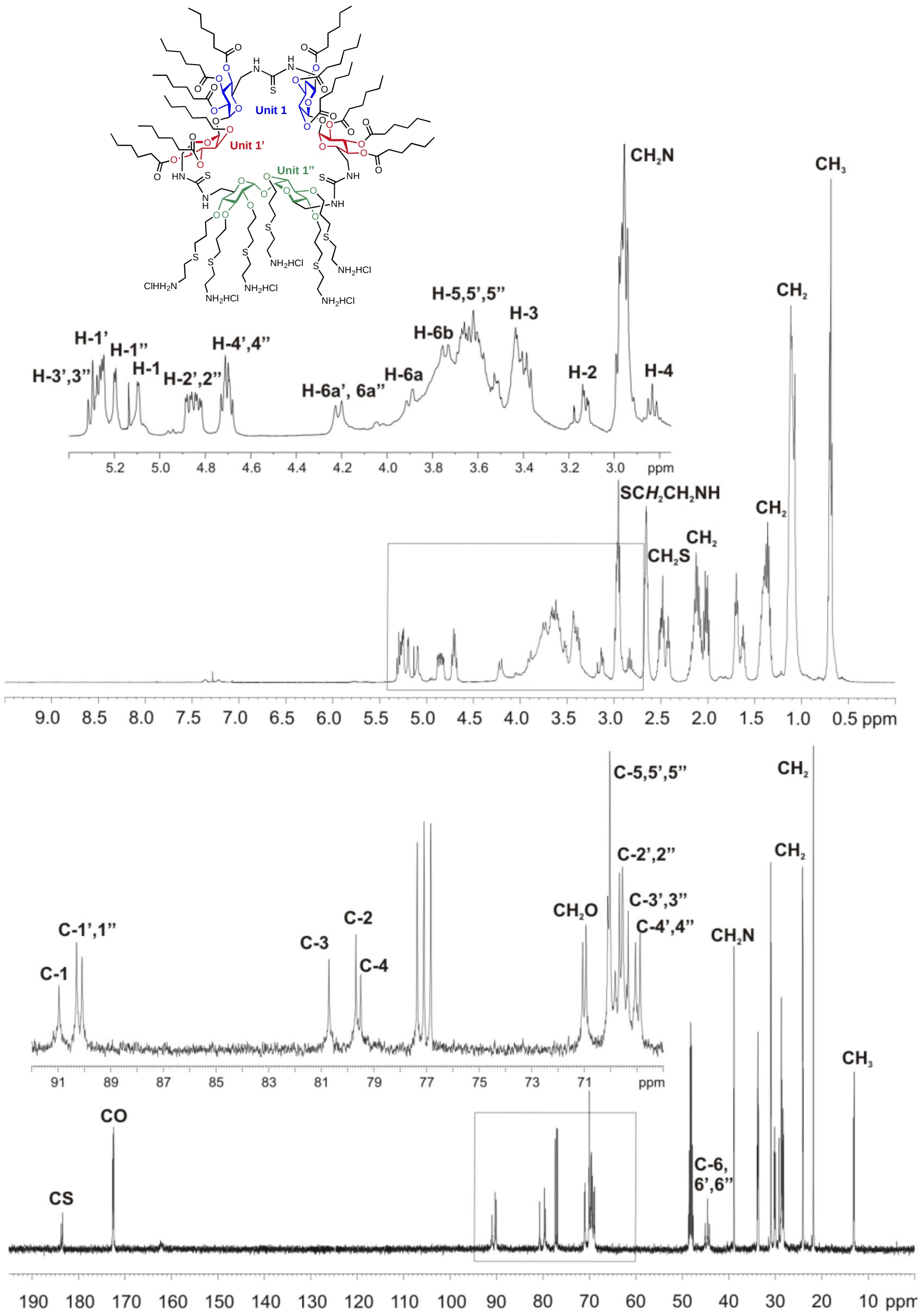

Figure S1. ^{1}H and ^{13}C NMR (500 MHz, 125.7 MHz, 5:1 CD_3OD-$CDCl_3$, 333 K) of **1.**

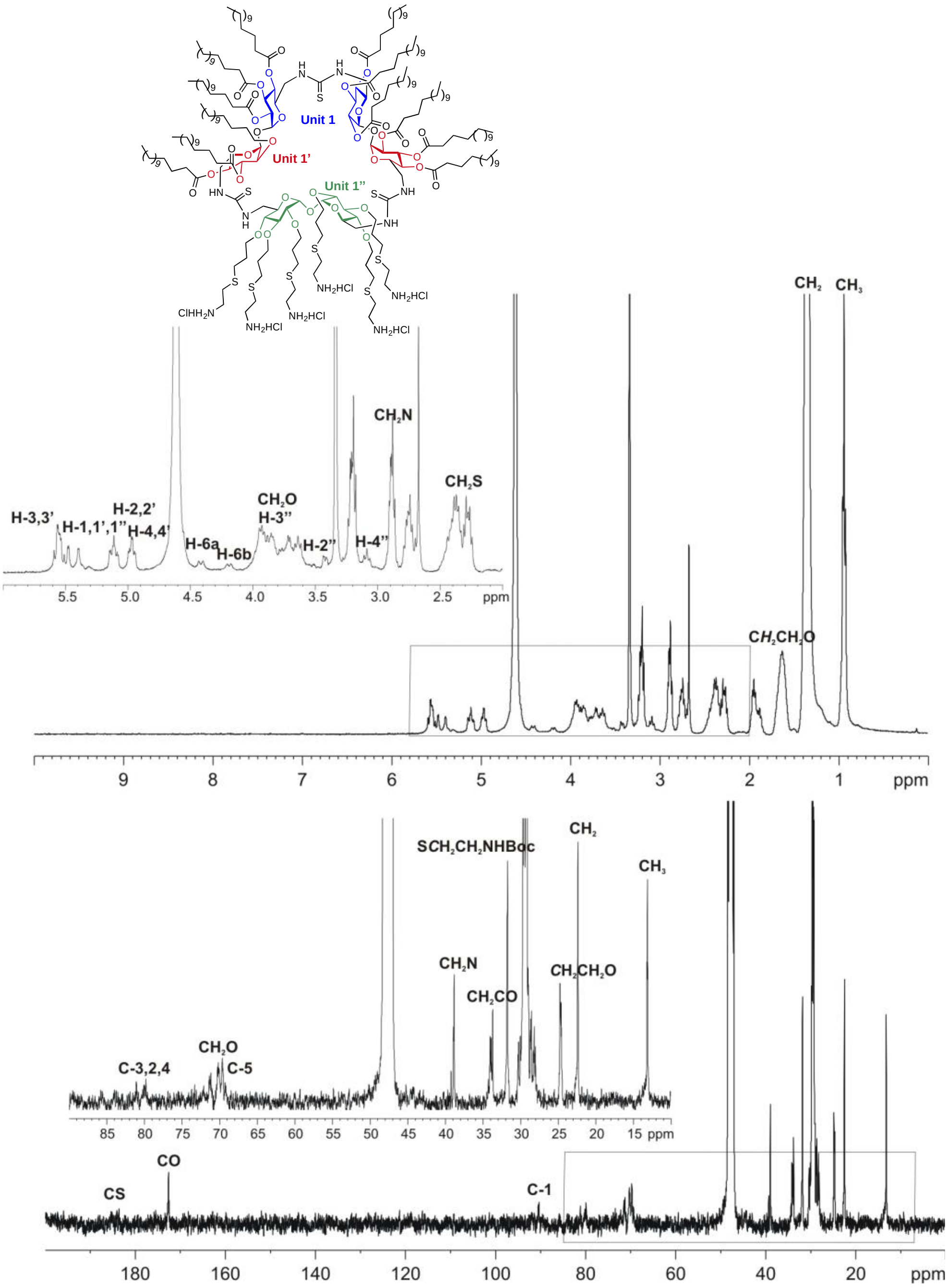

Figure S2. ^{1}H and ^{13}C NMR (500 MHz, 100.6 MHz, CD_3OD, 323 K) of **2**.

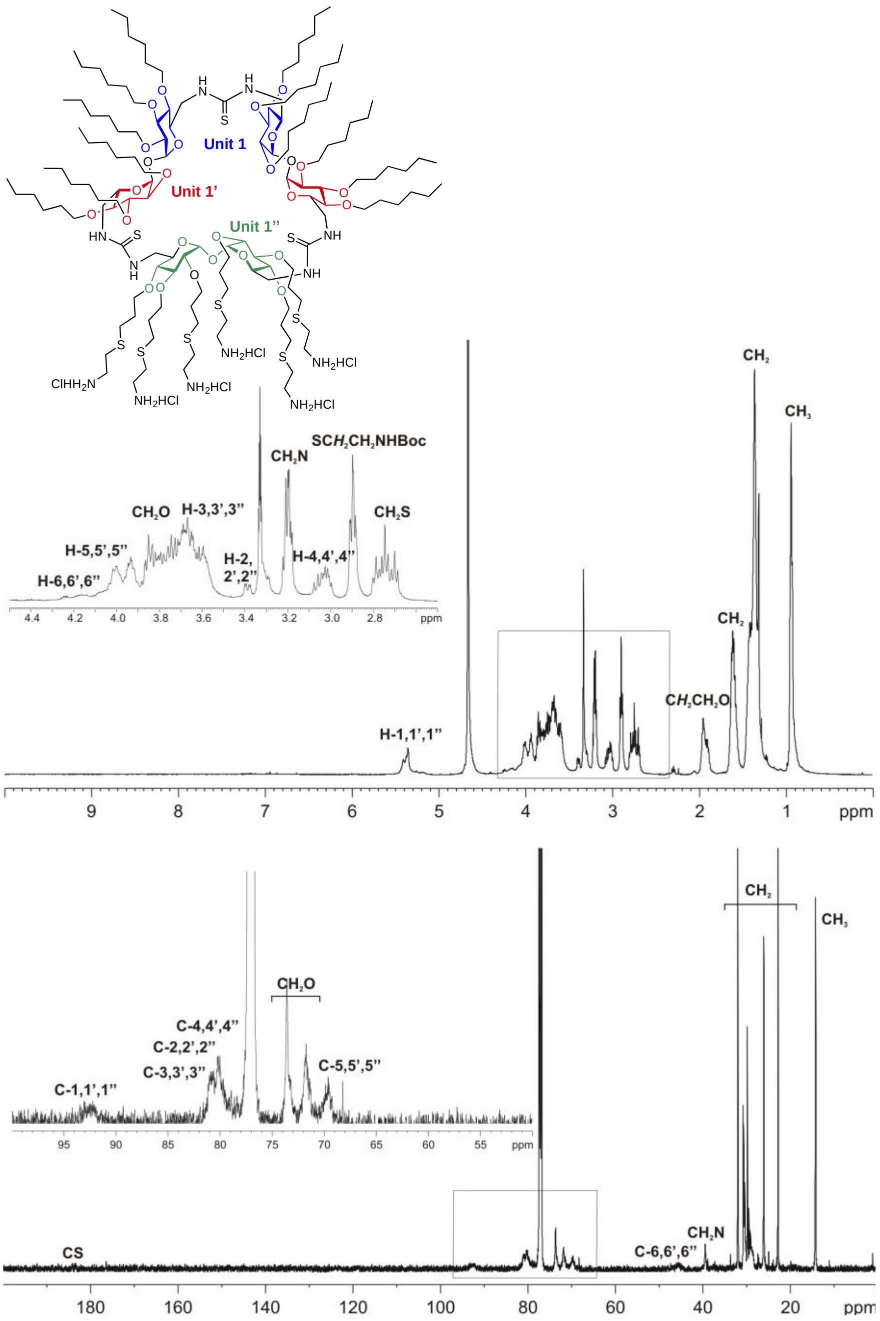

Figure S3. ^{1}H and ^{13}C NMR spectra (500 MHz, CD_3OD and 125.7 MHz, $CDCl_3$, 323 K) of **3**.

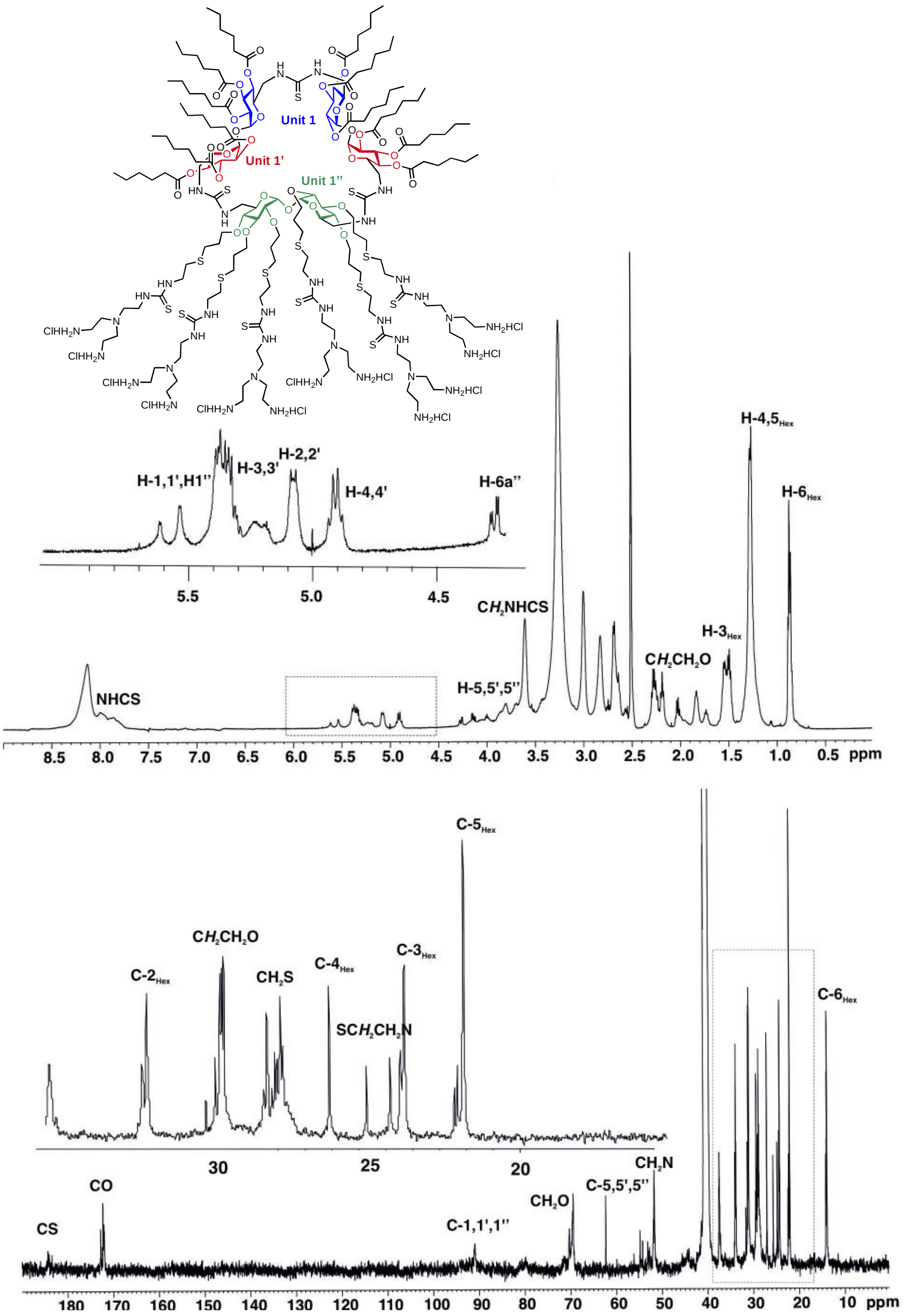

Figure S4. ^{1}H and ^{13}C NMR spectra (500 MHz, 125.7 MHz, DMSO-d_6, 343 K) of **4**.

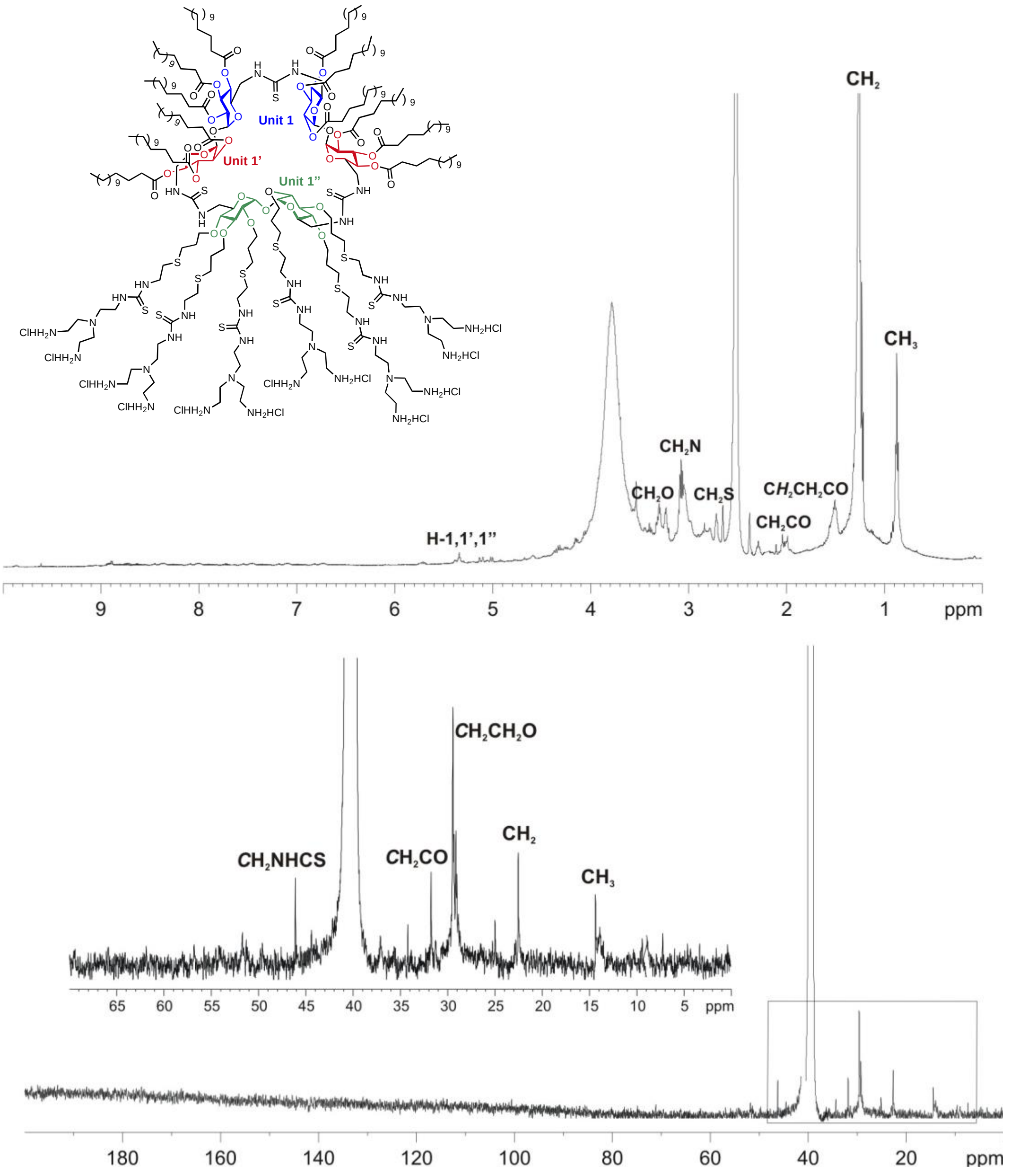

Figure S5. ^{1}H and ^{13}C NMR spectra (500 MHz, 125.7 MHz, 10:1 CD_3OD-D_2O, 323 K) of **5**.

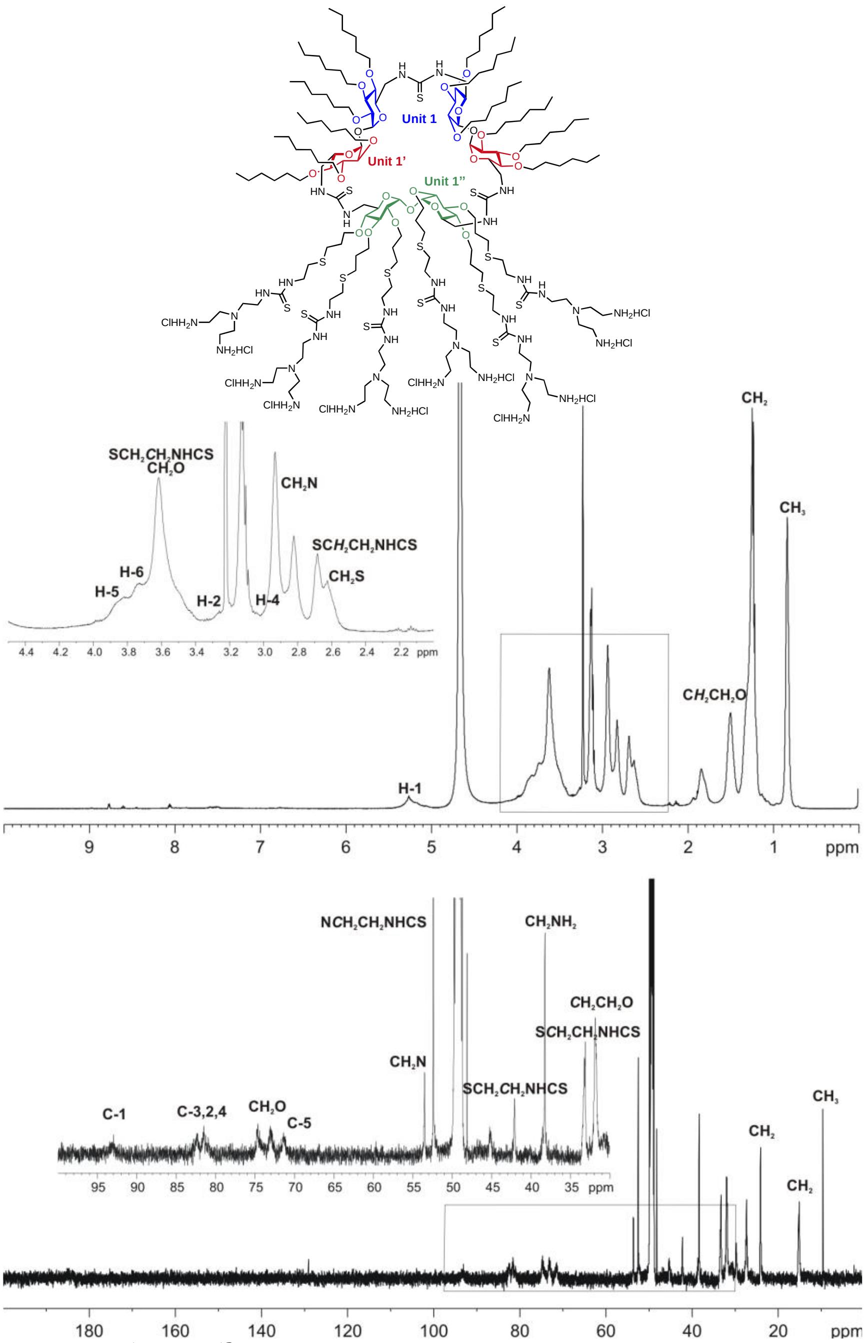

Figure S6. ^{1}H and ^{13}C NMR spectra (500 MHz, 125.7 MHz, CD_3OD) of **6**.

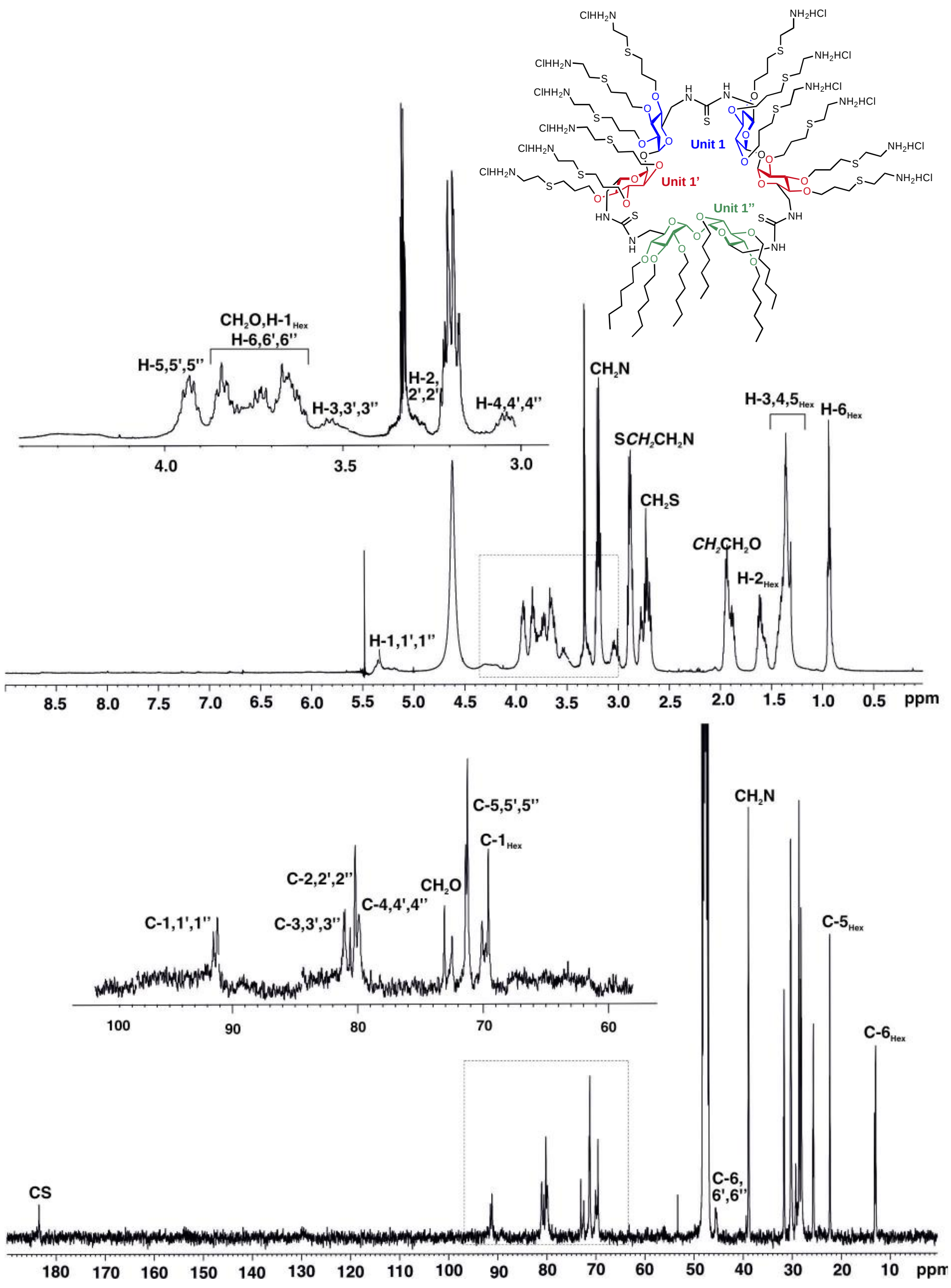

Figure S7. ^{1}H and ^{13}C NMR (500 MHz, 100.6 MHz, CD_3OD, 323 K) of **7**.

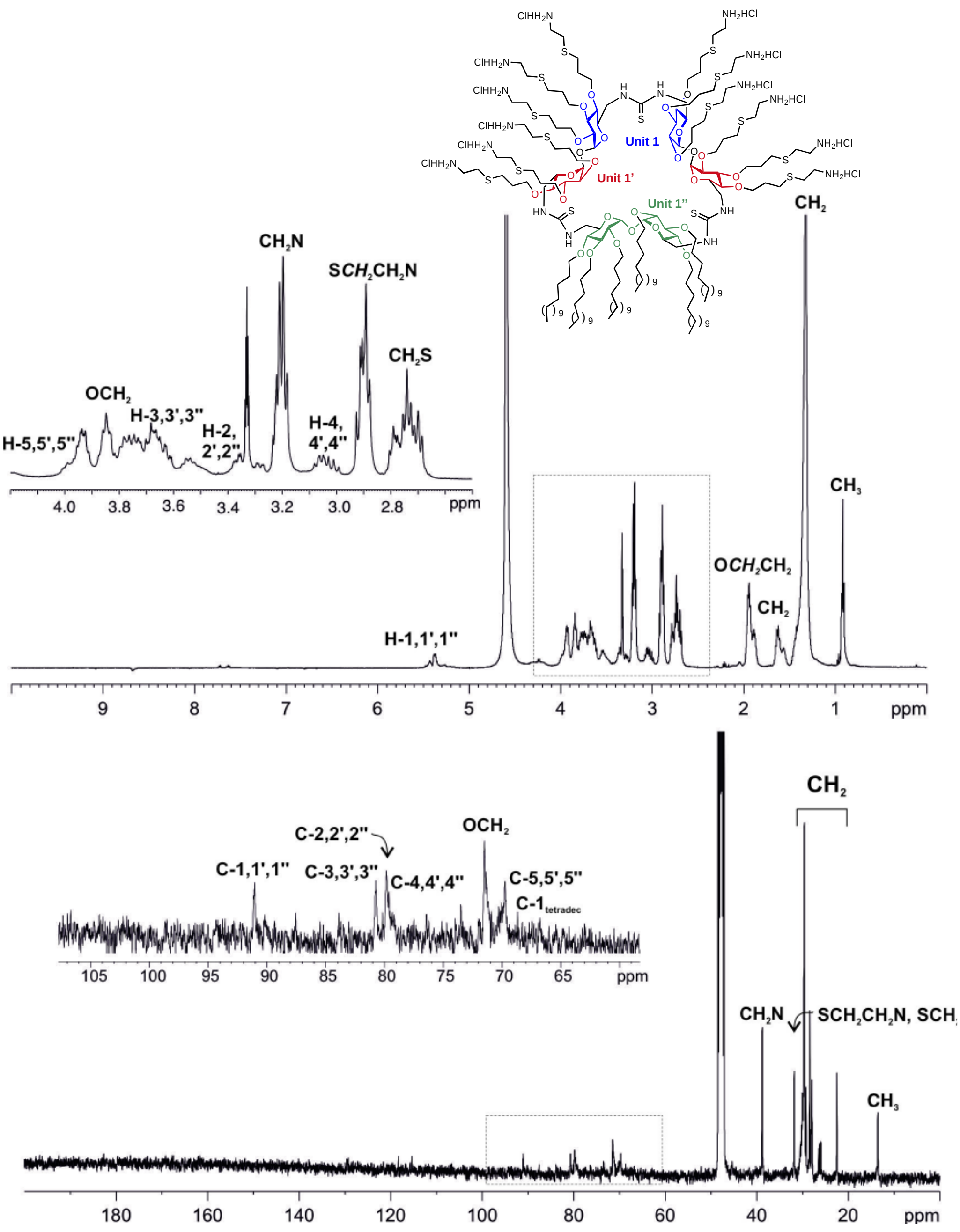

Figure S8. ^{1}H and ^{13}C NMR spectra (400 MHz, 100.6 MHz, 5:1 CD_3OD-D_2O, 323 K) of **8**.

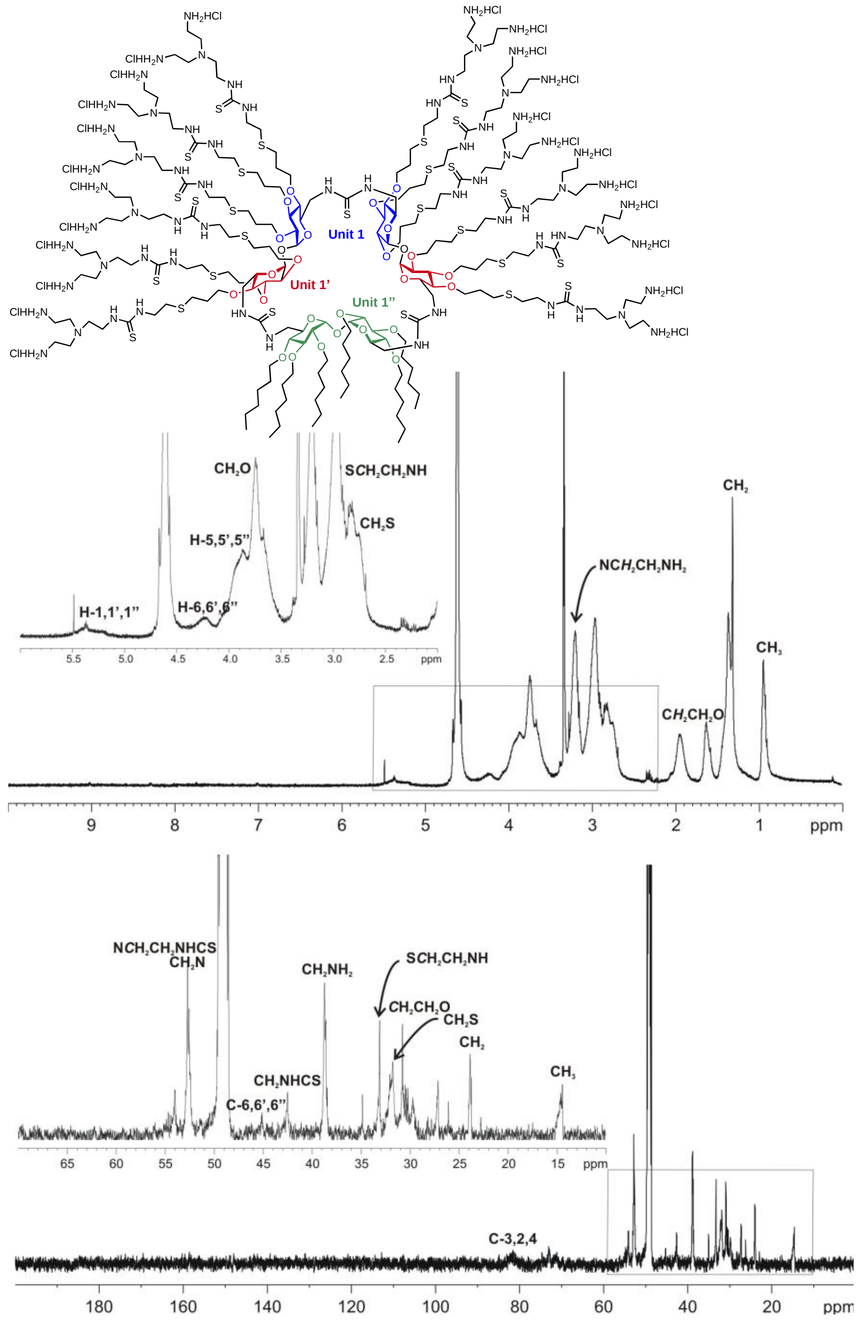

Figure S9. ^{1}H and ^{13}C NMR (500 MHz, 125.7 MHz, CD_3OD) of **9**.

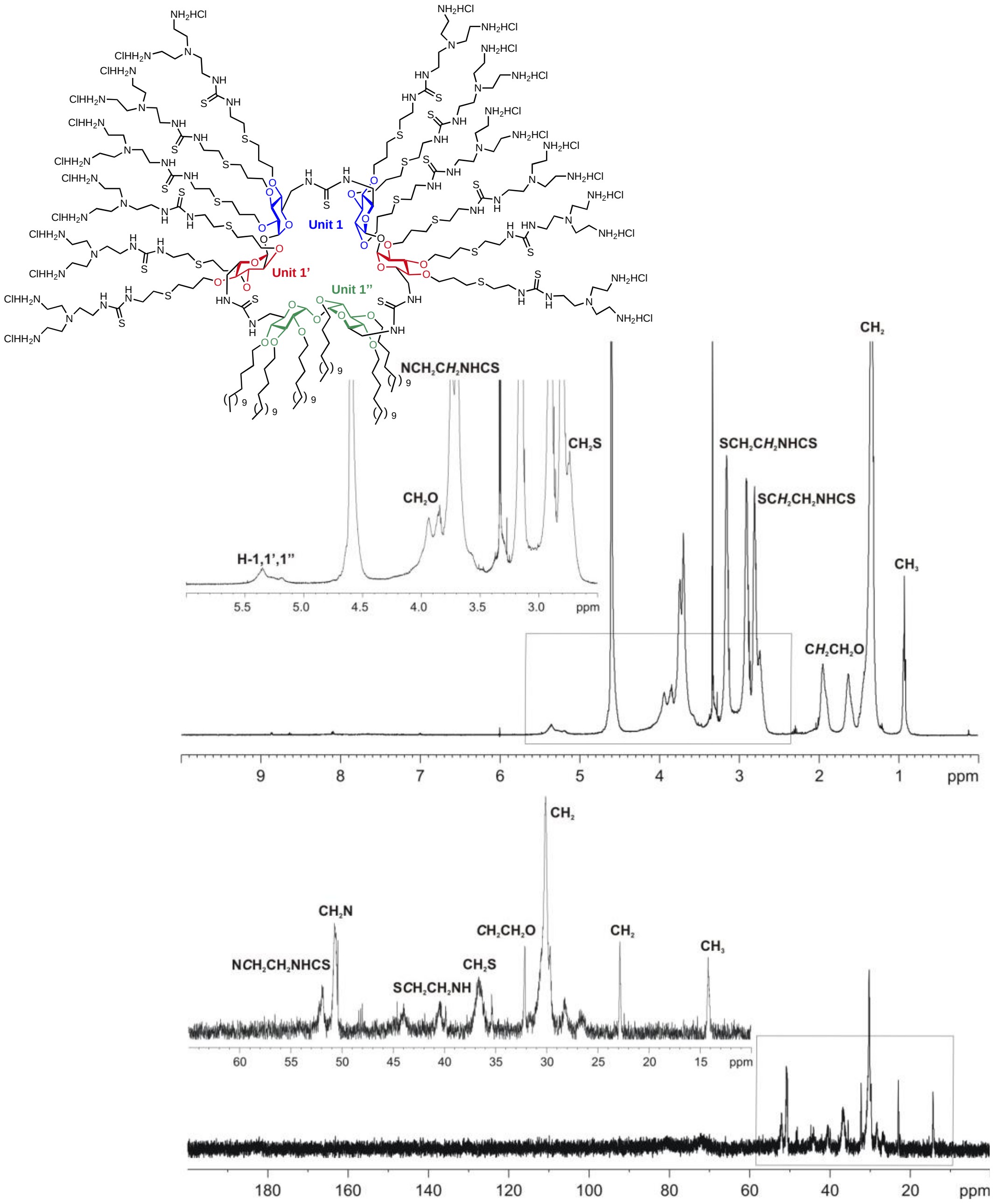

Figure S10. ^{1}H and ^{13}C NMR (500 MHz, CD3OD, 323 K and 125.7 MHz, D_2O) of **10**.

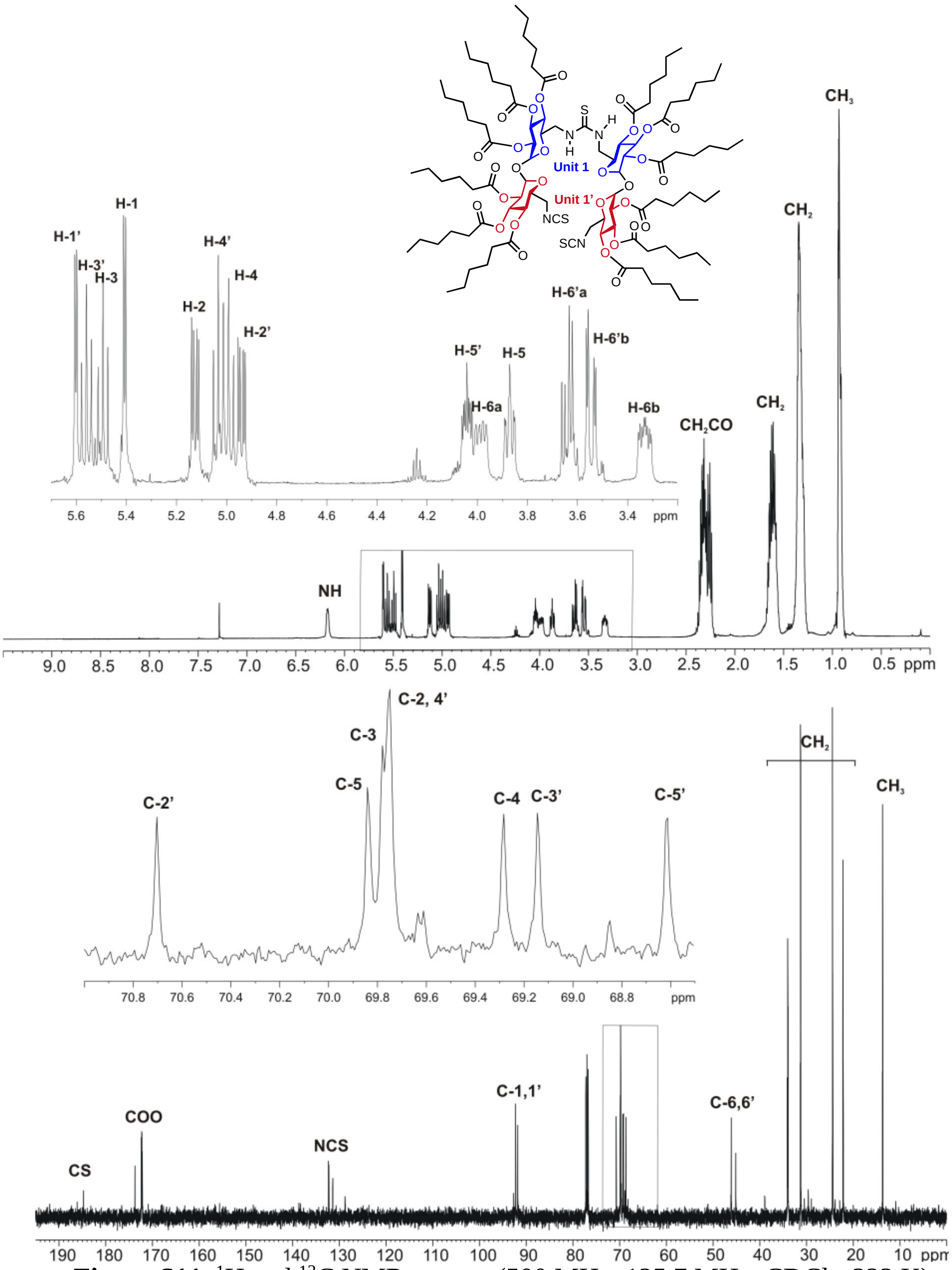

Figure S11. ^{1}H and ^{13}C NMR spectra (500 MHz, 125.7 MHz, $CDCl_3$, 323 K) of **14**.

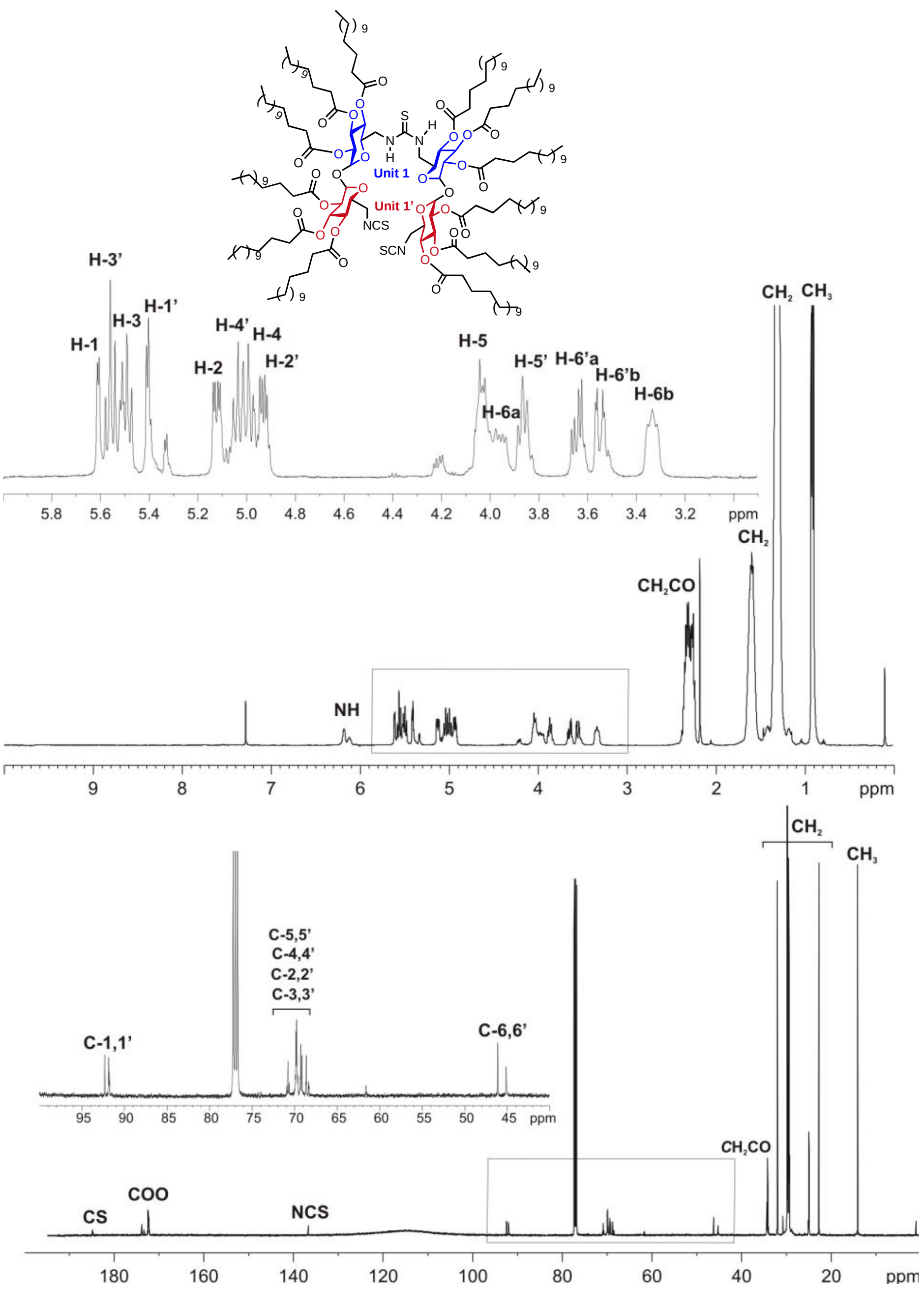

Figure S12. ^{1}H and ^{13}C NMR spectra (500 MHz, 125.7 MHz, $CDCl_3$, 323 K) of **15**.

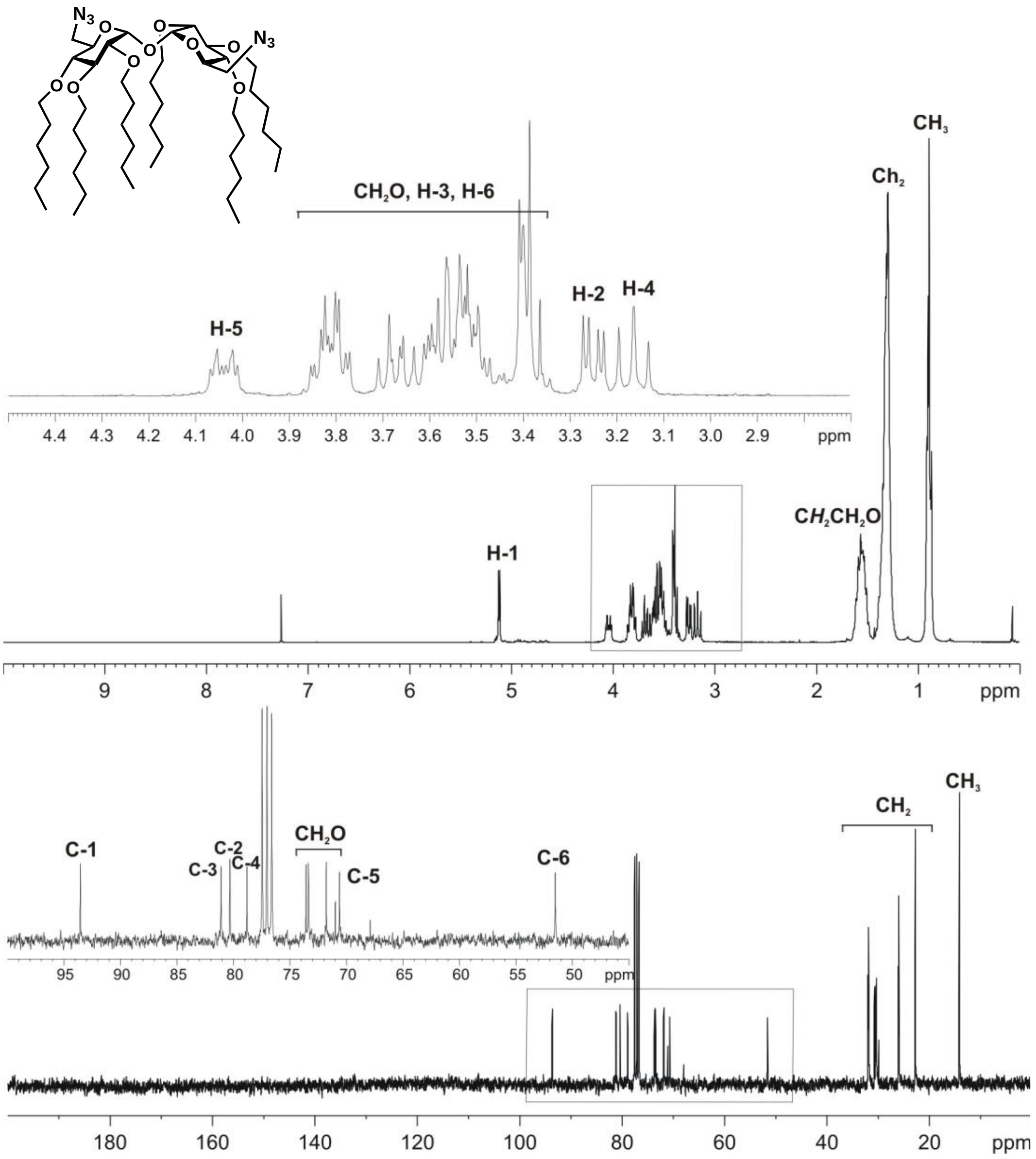

Figure S13. ^{1}H and ^{13}C NMR spectra (300 MHz, 75.5 MHz, $CDCl_3$) of **17**.

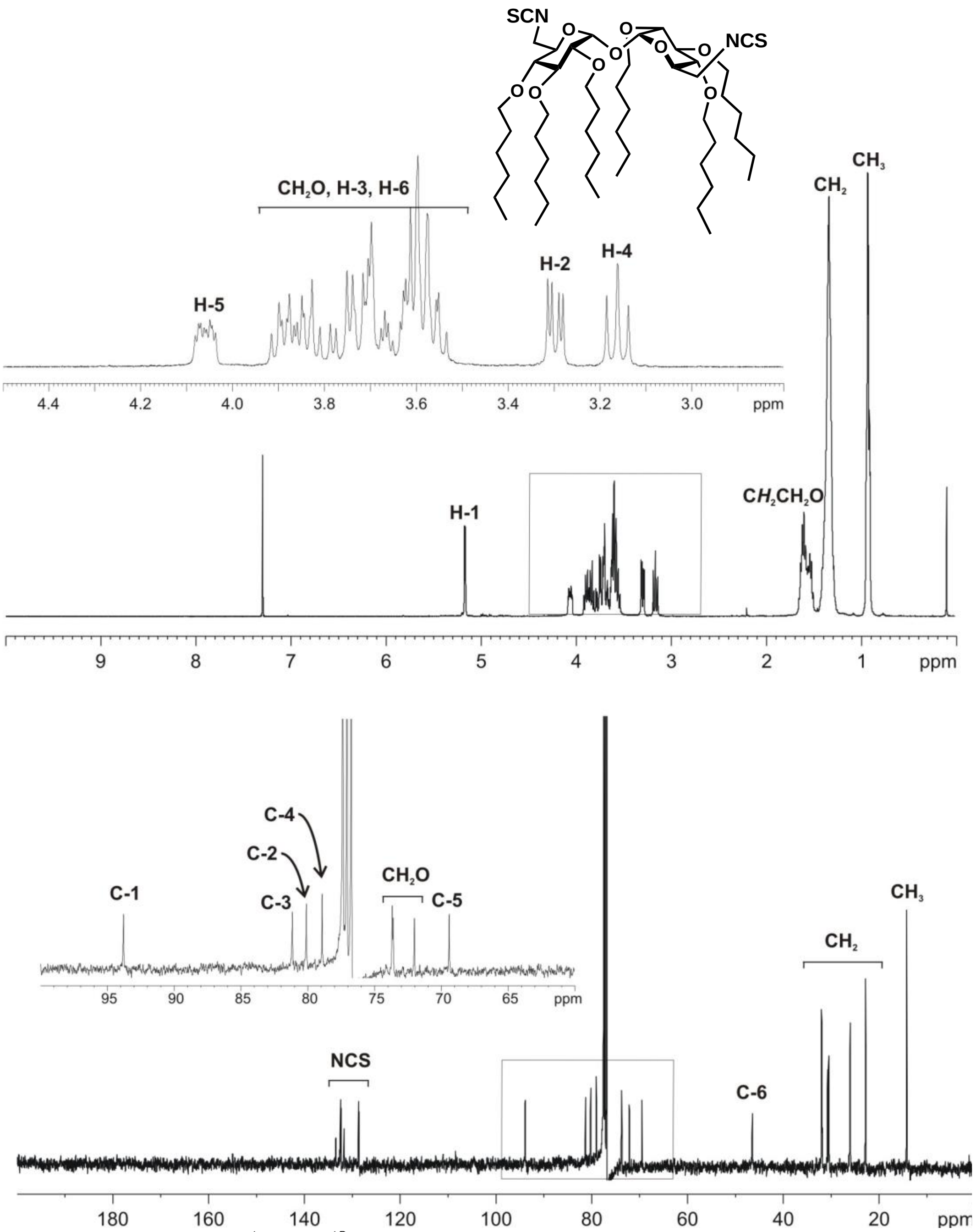

Figure S14. ^{1}H and ^{13}C NMR spectra (400 MHz, 100.6 MHz, $CDCl_3$) of **18**.

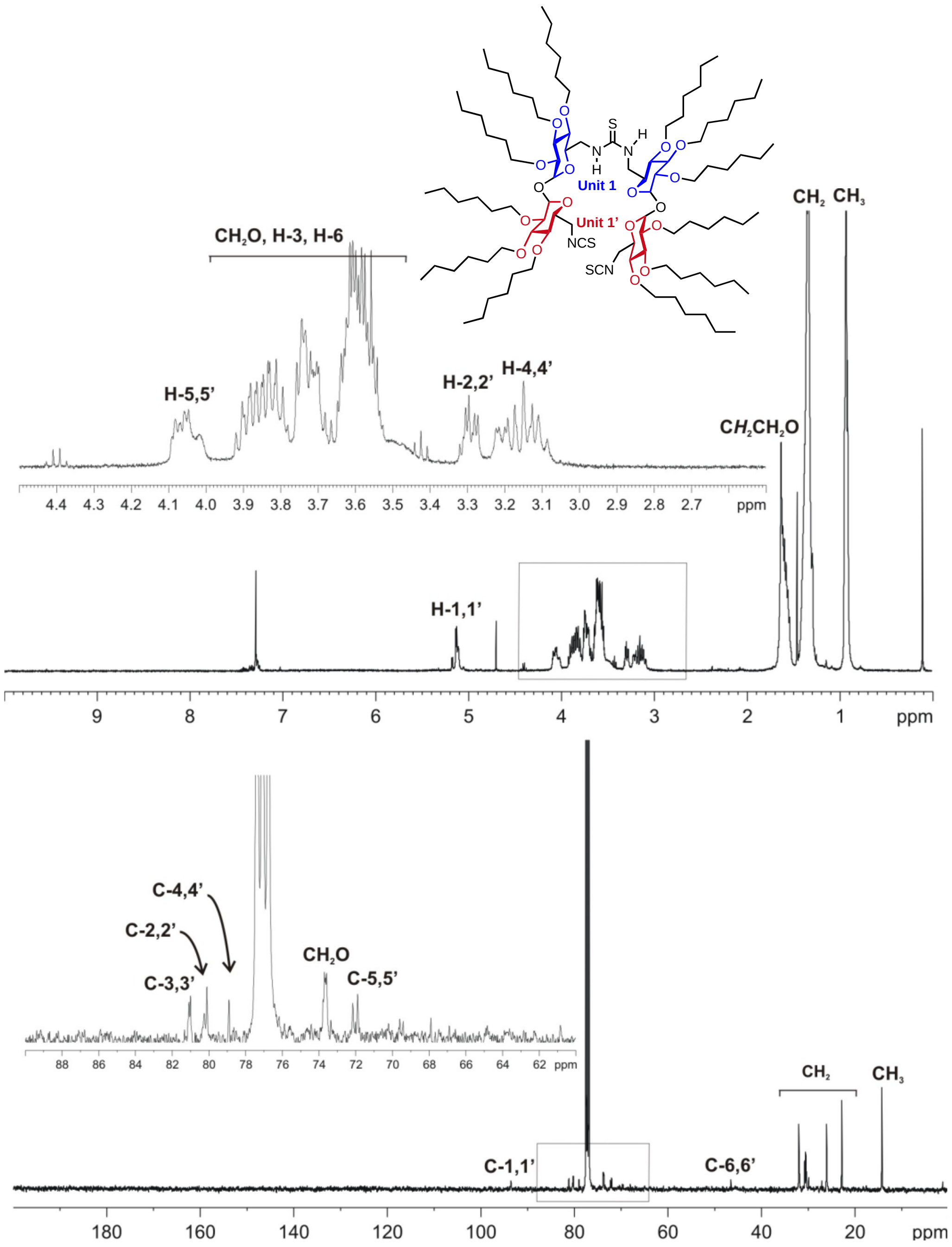

Figure S15. ^{1}H and ^{13}C NMR spectra (400 MHz, 100.6 MHz, $CDCl_3$, 323 K) of **19**.

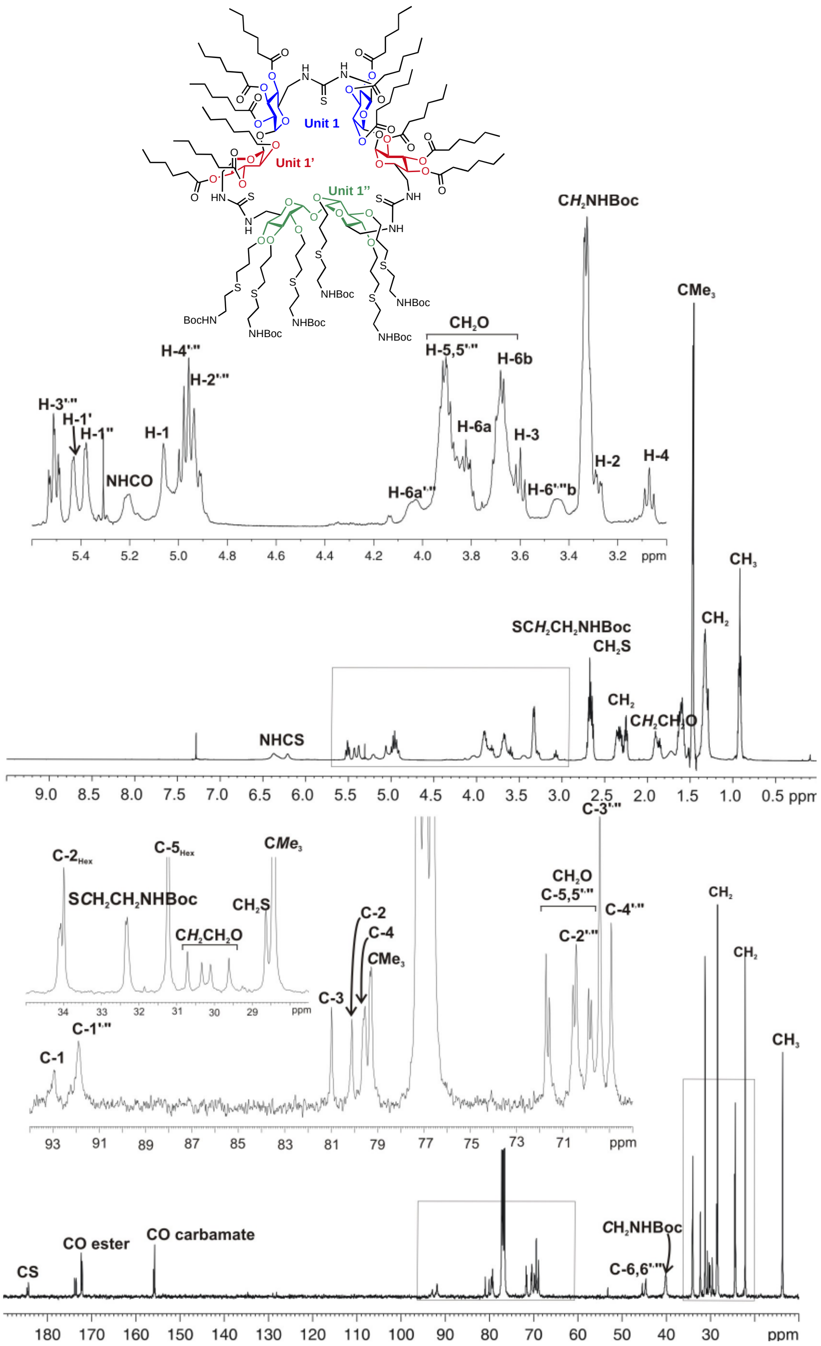

Figure S16. ^{1}H and ^{13}C NMR spectra (500 MHz, 100.6 MHz, $CDCl_3$, 323 K) of **21**.

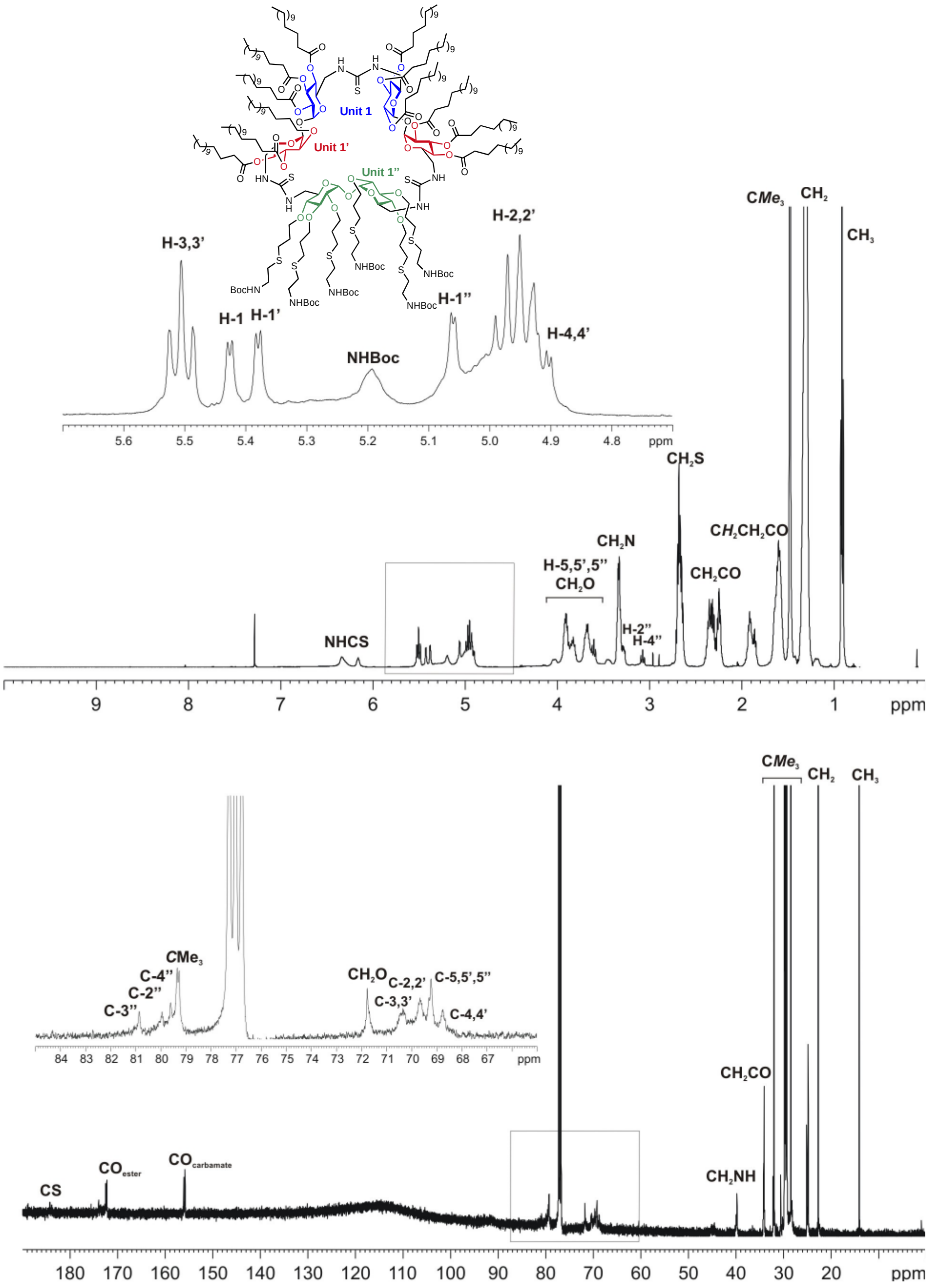

Figure S17. ^{1}H and ^{13}C NMR spectra (500 MHz, 125.7 MHz, $CDCl_3$, 333 K) of **22**.

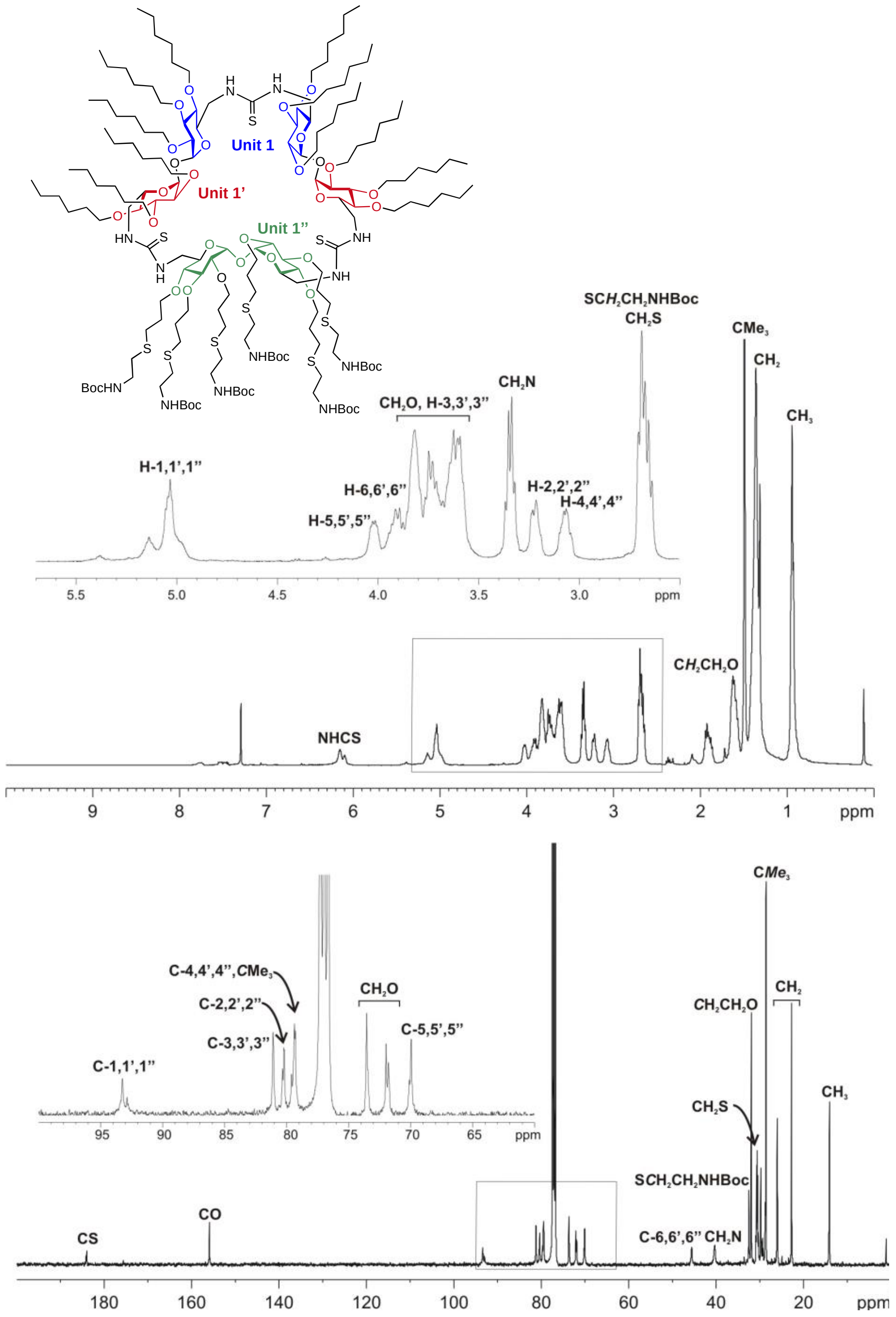

Figure S18. ^{1}H and ^{13}C NMR (400 MHz, 100.6 MHz, $CDCl_3$, 323 K) of **23**.

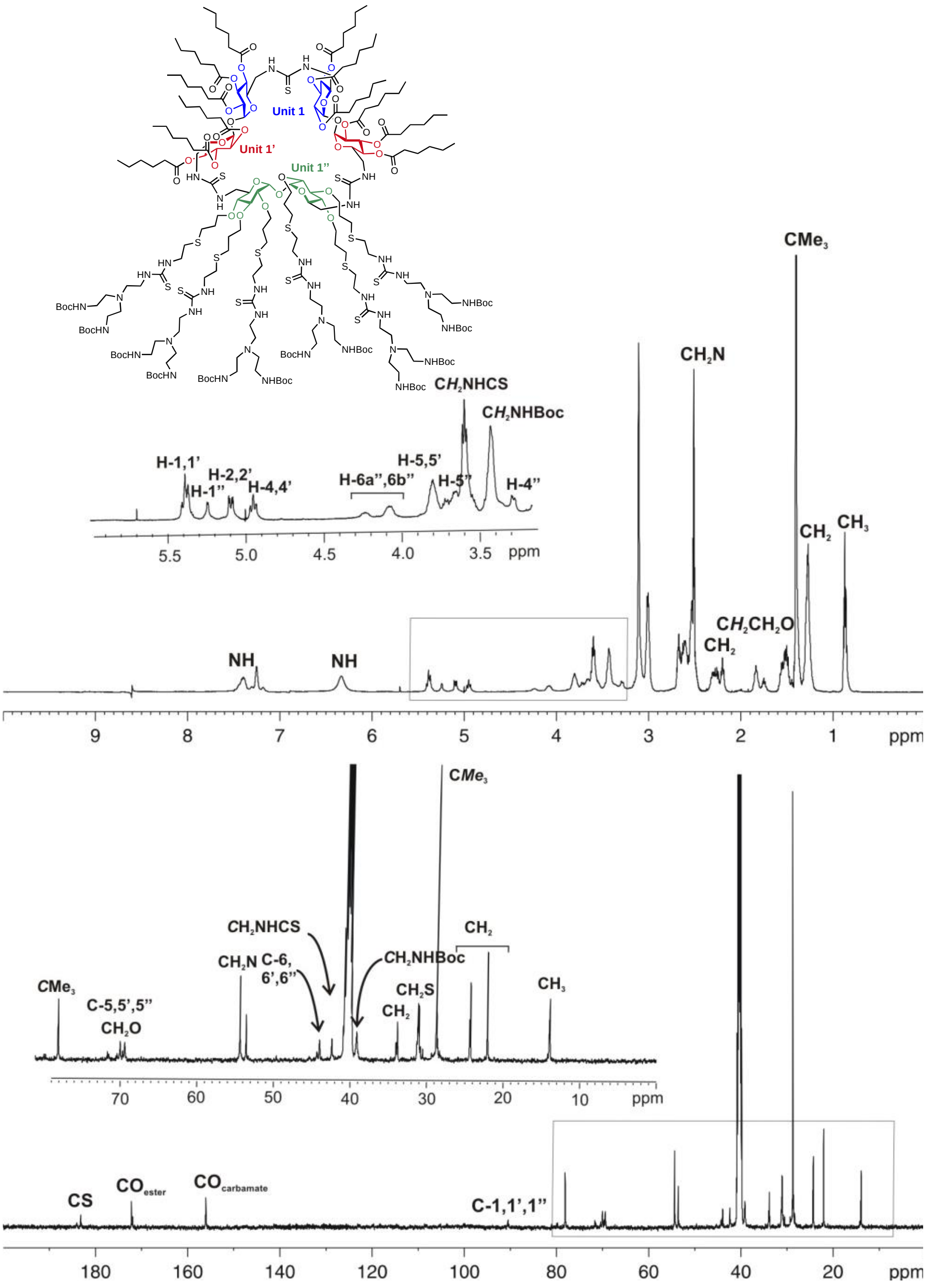

Figure S19. ^{1}H and ^{13}C NMR spectra (500 MHz, 125.7 MHz, DMSO-d_6, 343 K) of **24**.

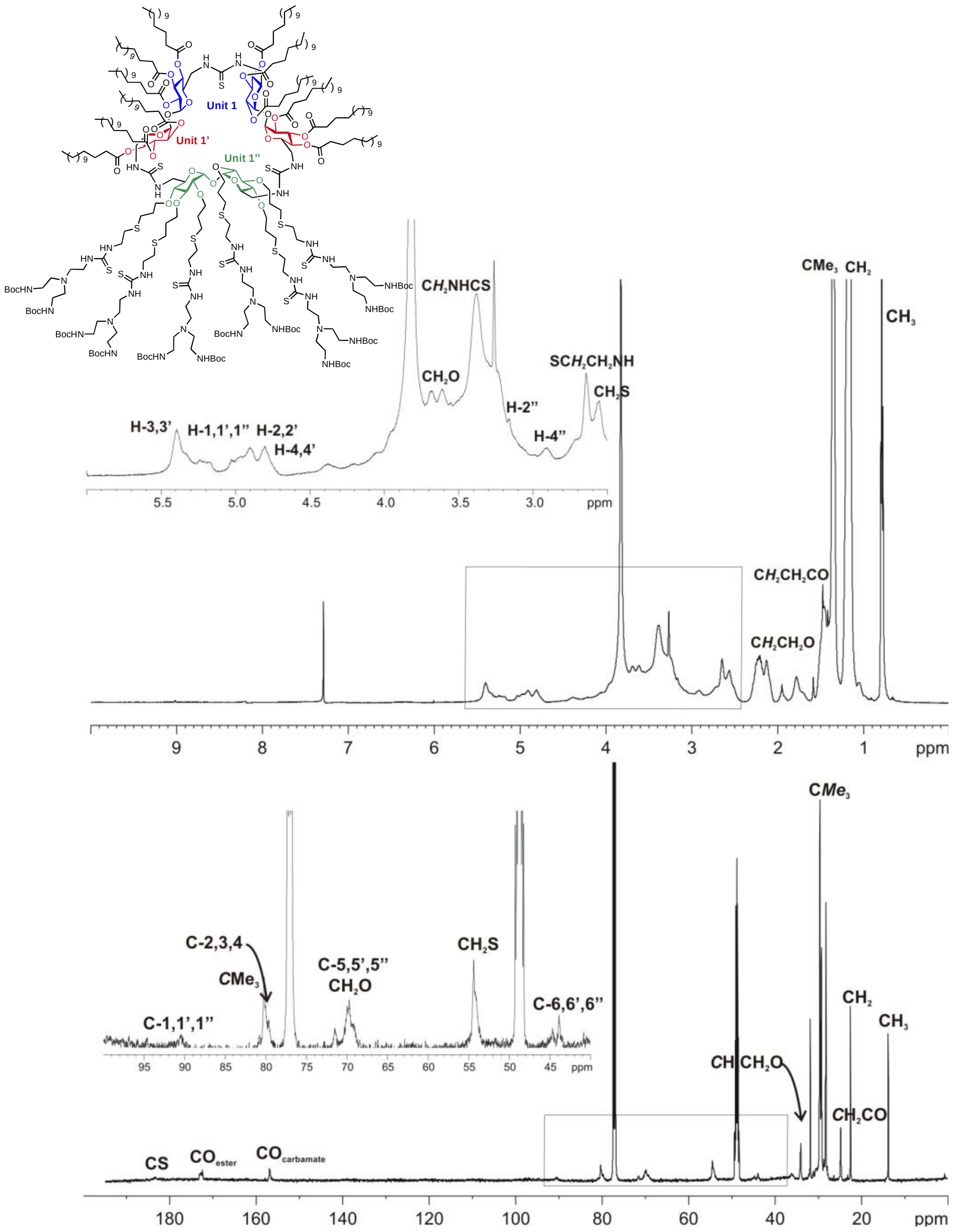

Figure S20. ^{1}H and ^{13}C NMR spectra (500 MHz, 125.7 MHz, 3:1 CD_3OD-$CDCl_3$, 323 K) of **25**.

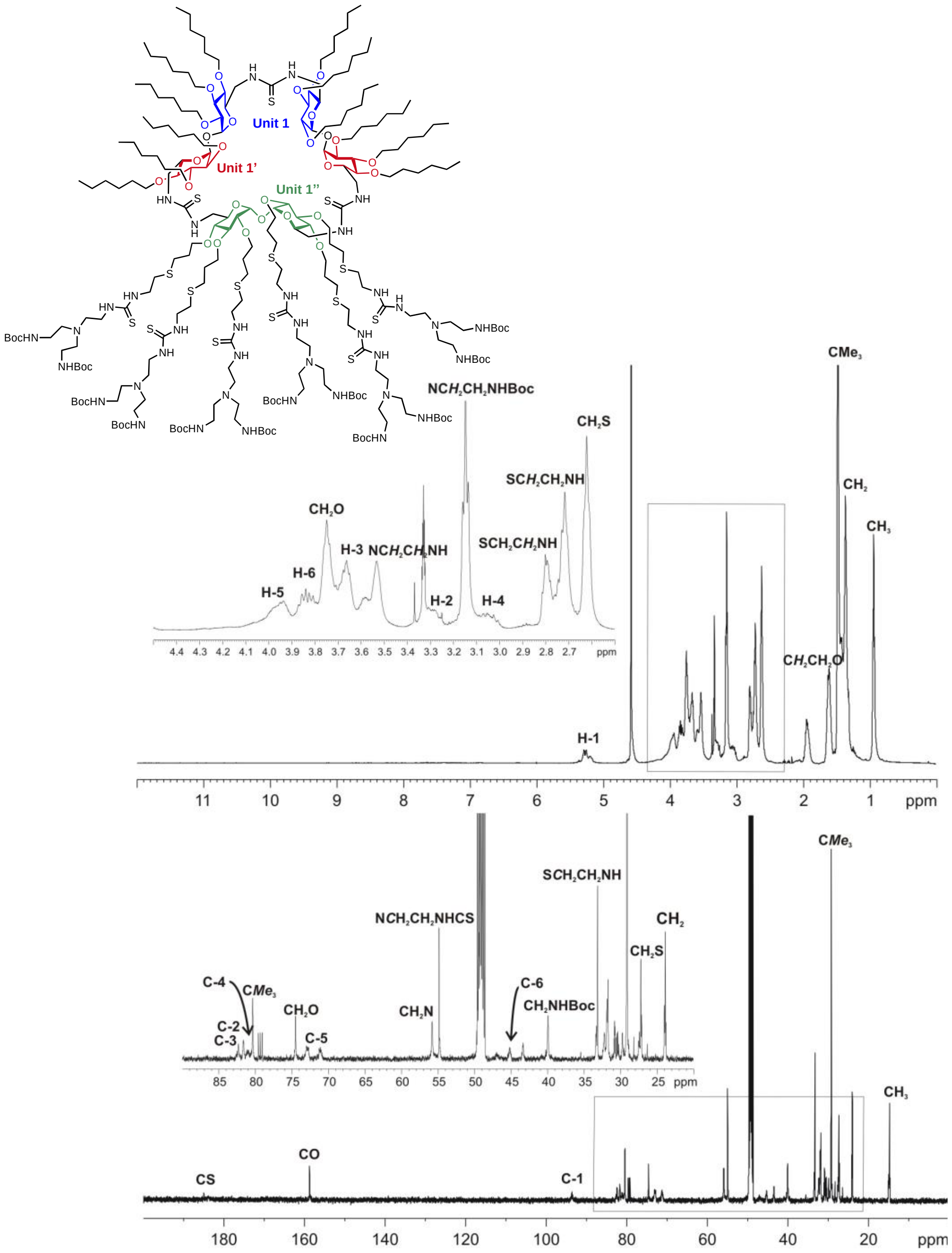

Figure S21. ^{1}H and ^{13}C NMR (500 MHz, 125.7 MHz, CD_3OD, 323 K) of **26**.

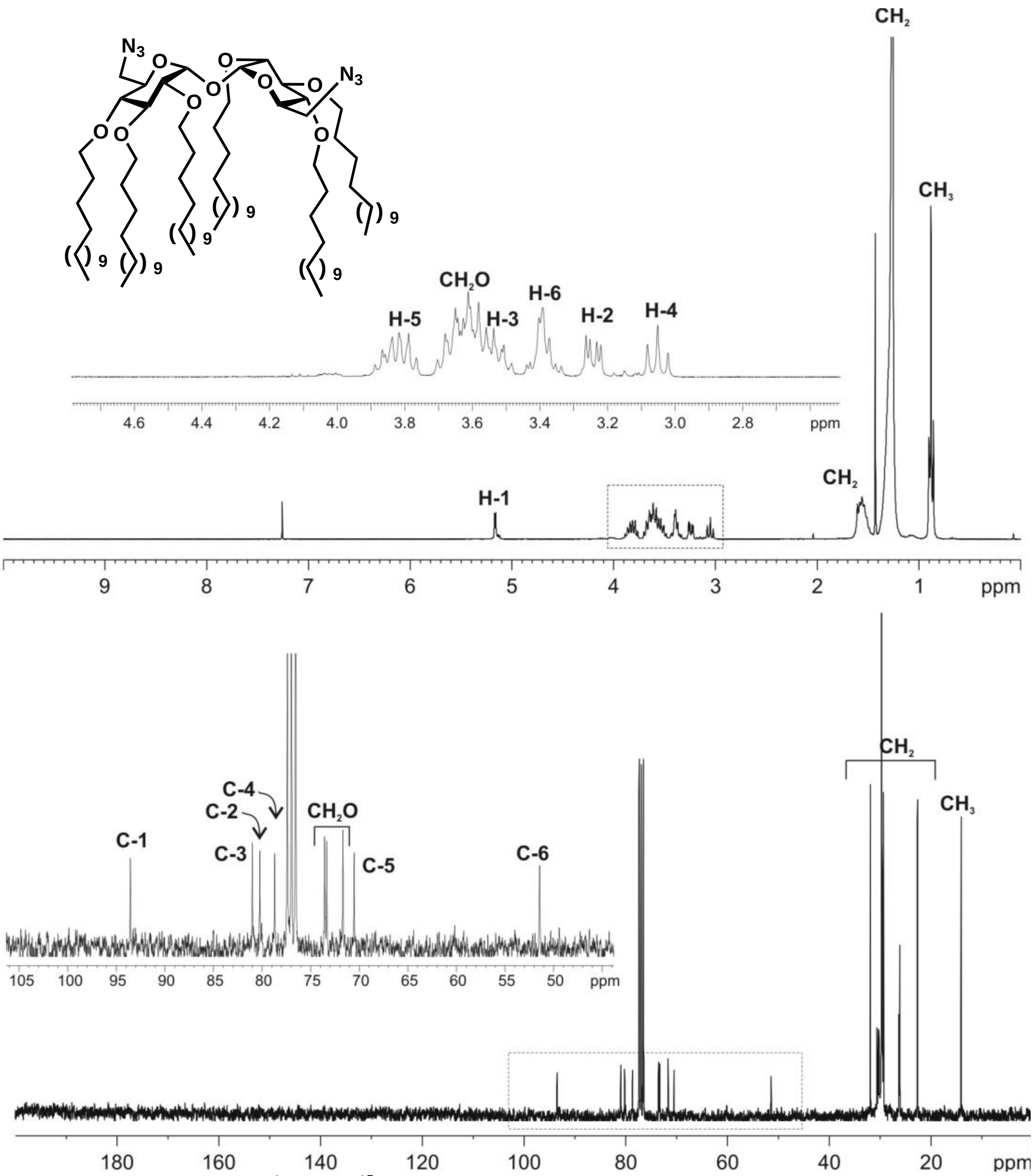

Figure S22. ^{1}H and ^{13}C NMR spectra (300 MHz, 75.5 MHz, $CDCl_3$) of **27**.

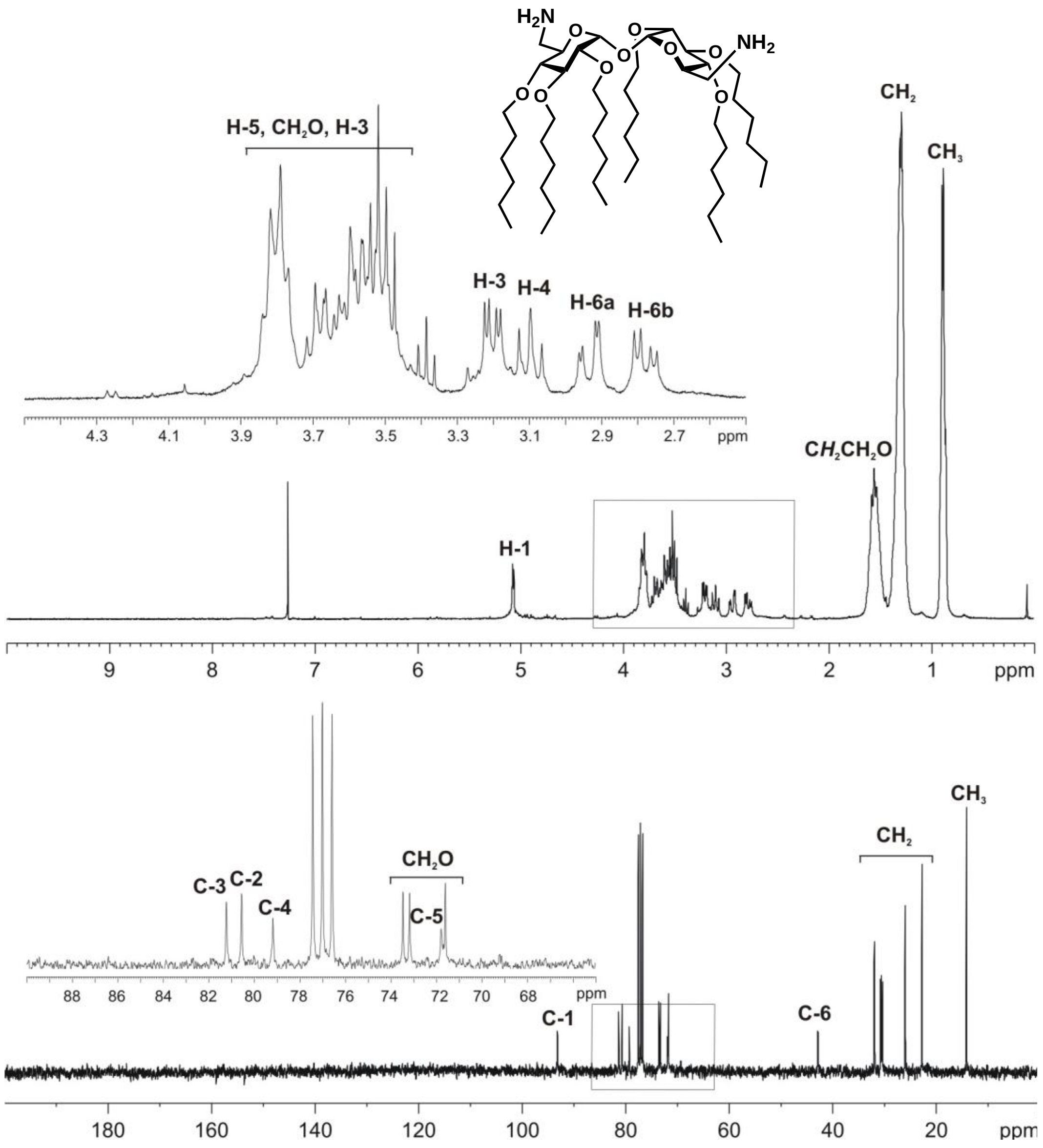

Figure S23. ^{1}H and ^{13}C NMR spectra (300 MHz, 75.5 MHz, $CDCl_3$) of **28**.

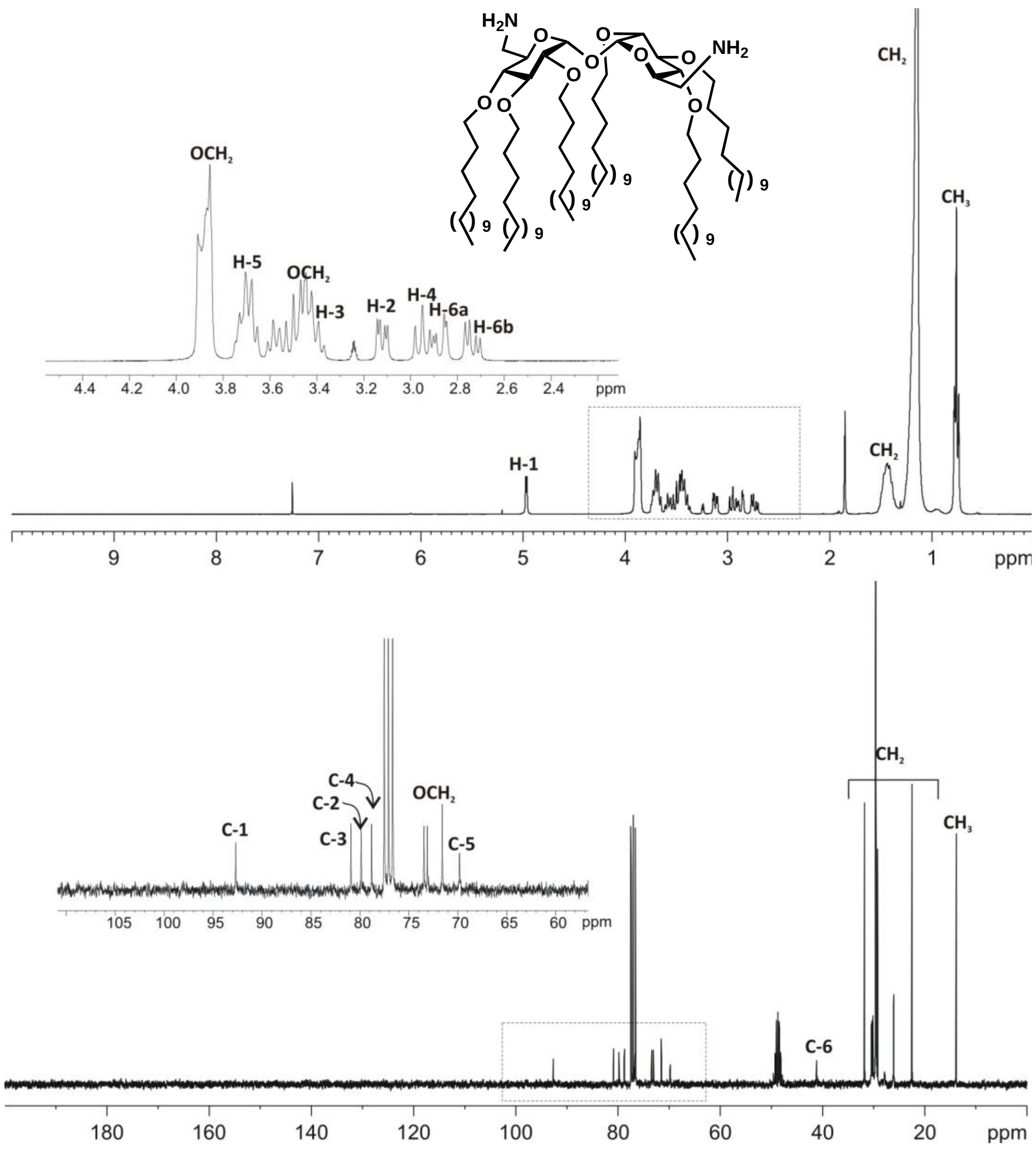

Figure S24. ^{1}H and ^{13}C NMR spectra (300 MHz, 75.5 MHz, 10:1 $CDCl_3$-CD_3OD) of **29**.

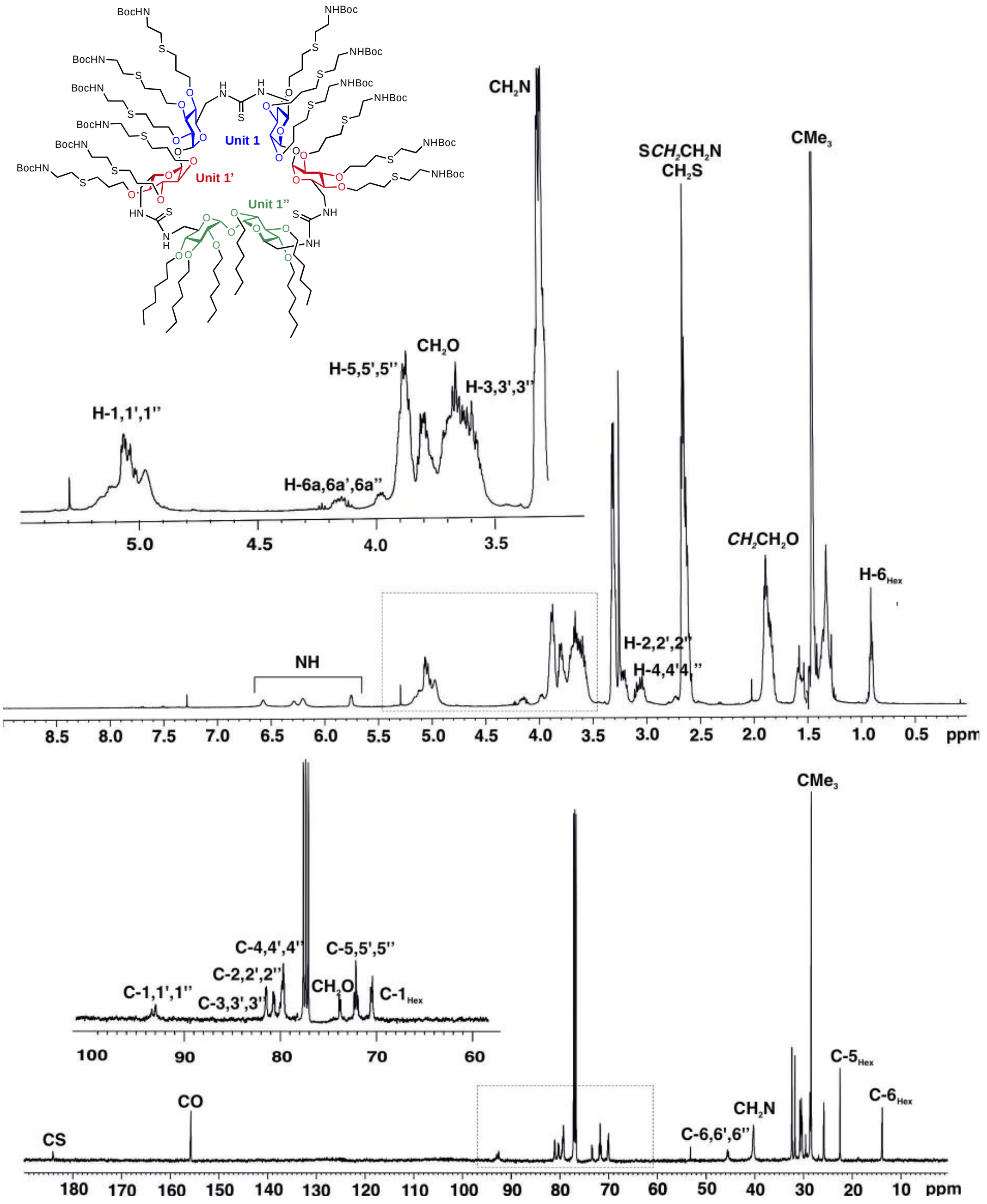

Figure S25. ^{1}H and ^{13}C NMR spectra (500 MHz, 125.7 MHz, $CDCl_3$, 333 K) of **31**.

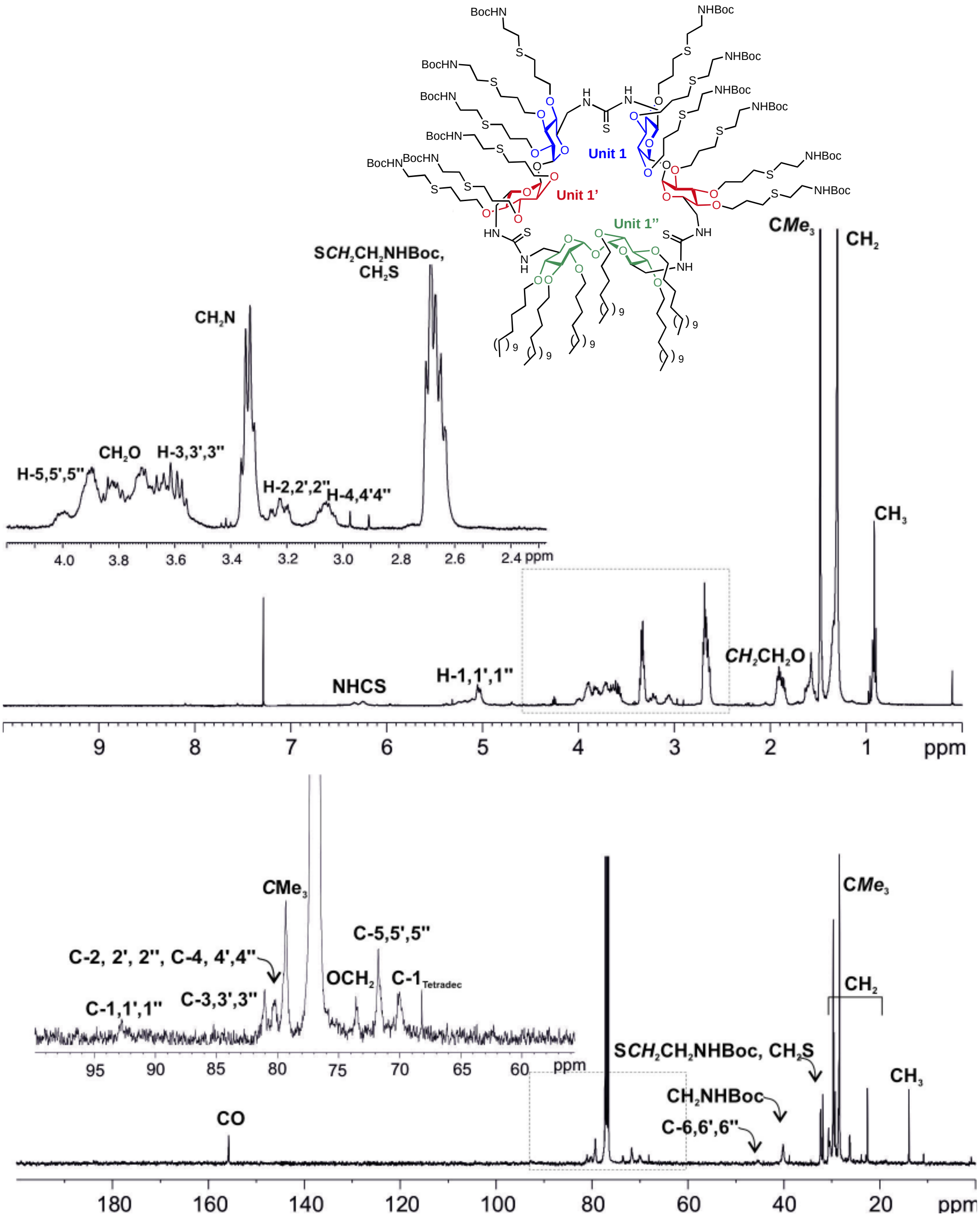

Figure S26. ^{1}H and ^{13}C NMR spectra (400 MHz, 100.6 MHz, $CDCl_3$, 323 K) of **32**.

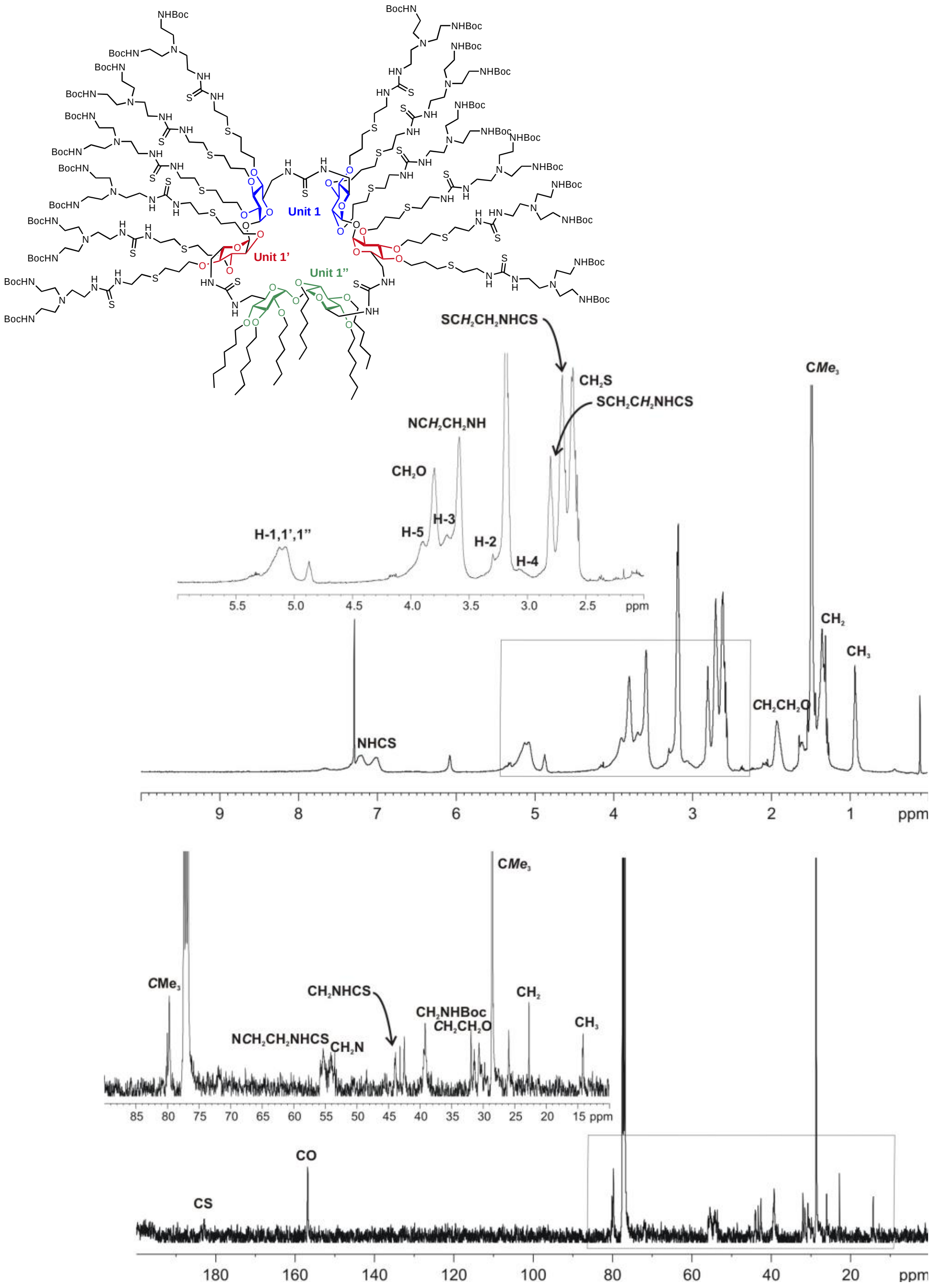

Figure S27. ^{1}H and ^{13}C NMR (400 MHz, 100.6 MHz, $CDCl_3$, 333 K) of **33**.

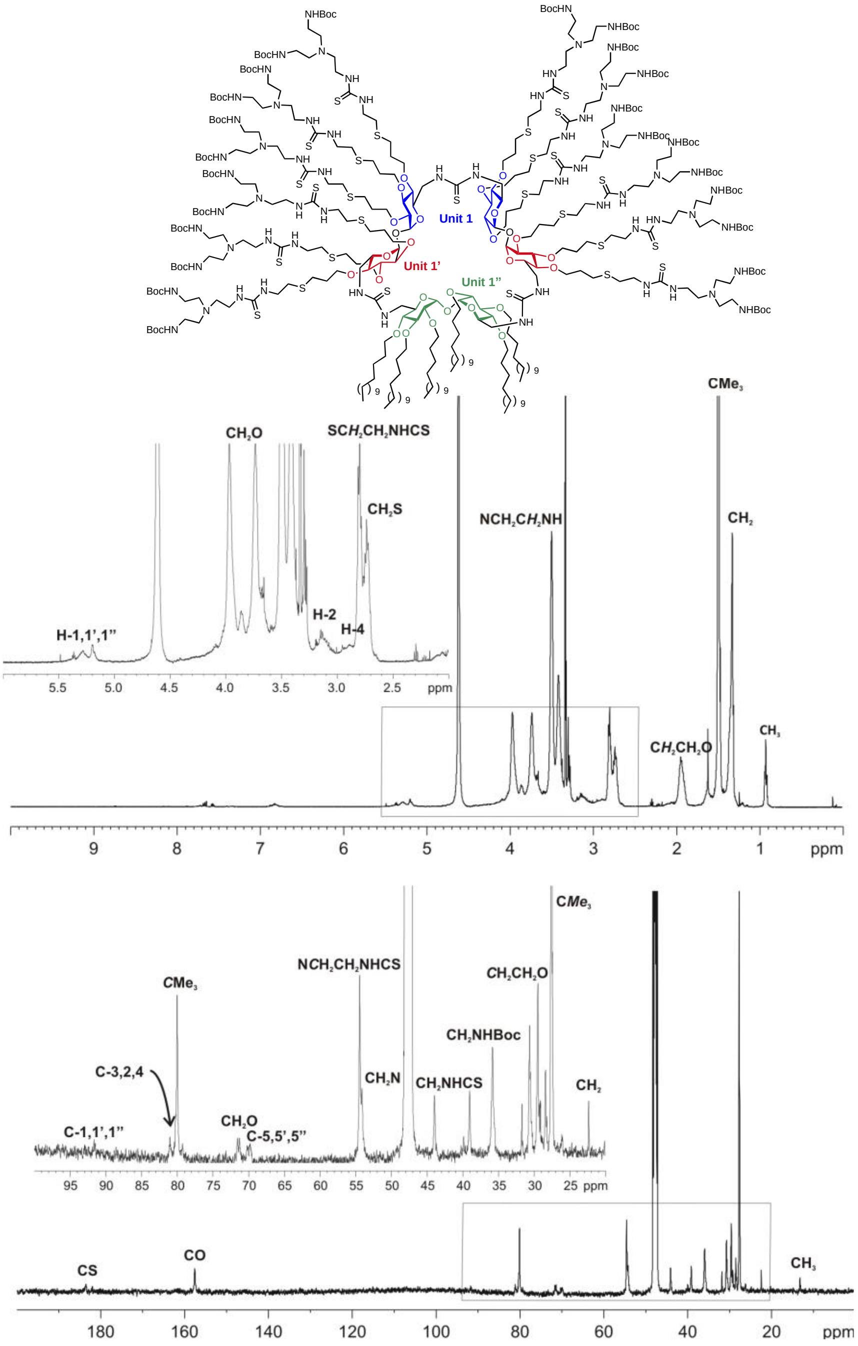

Figure S28. ^{1}H and ^{13}C NMR spectra (500 MHz, 125.7 MHz, CD_3OD, 323 K) of **34**.

Mass Spectra

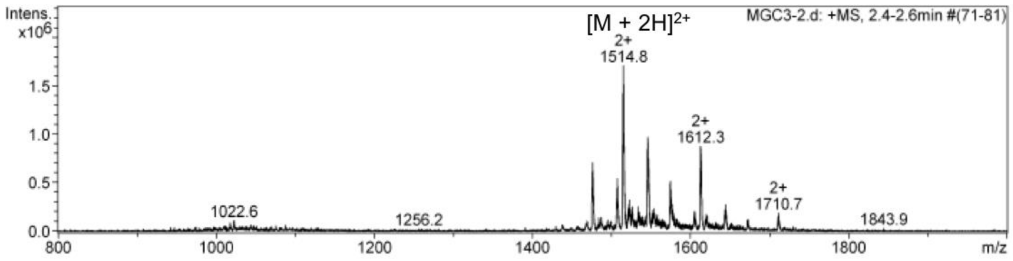

Figure S29. ESI-MS spectrum of **1**.

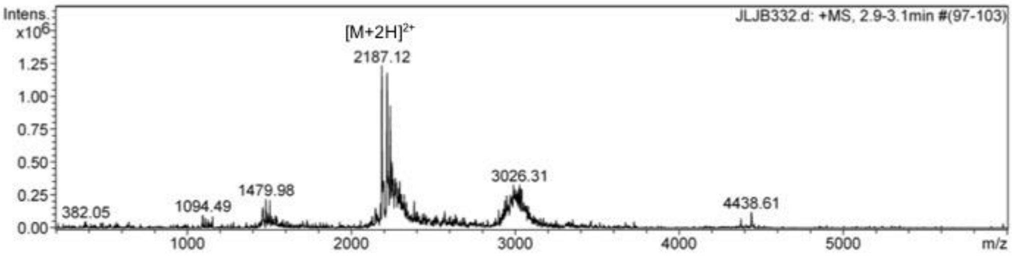

Figure S30. ESI-MS spectrum of **2**.

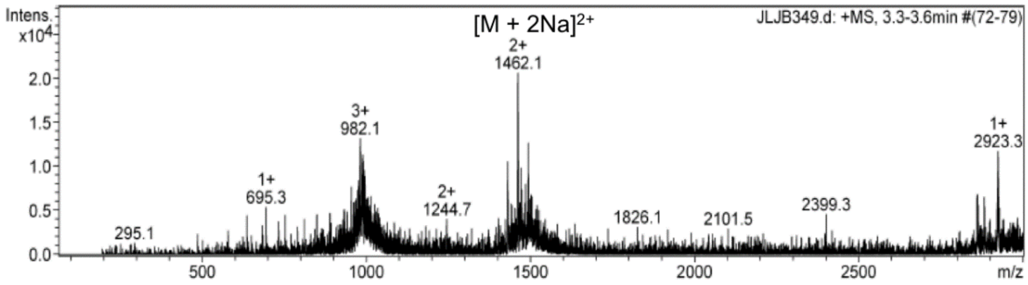

Figure S31. ESI-MS spectrum of **3**.

	Theoretical average mass (g/mol)	Experimental average mass (g/mol)	Mass accuracy (ppm)
[**4** + H]$^+$	4143.88	4143.89	2
[**4** + ssDNA 1/1 + H]$^+$	7728.28	7728.39	14
[**4** + ssDNA 1/2 + H]$^+$	11312.68	11312.81	11

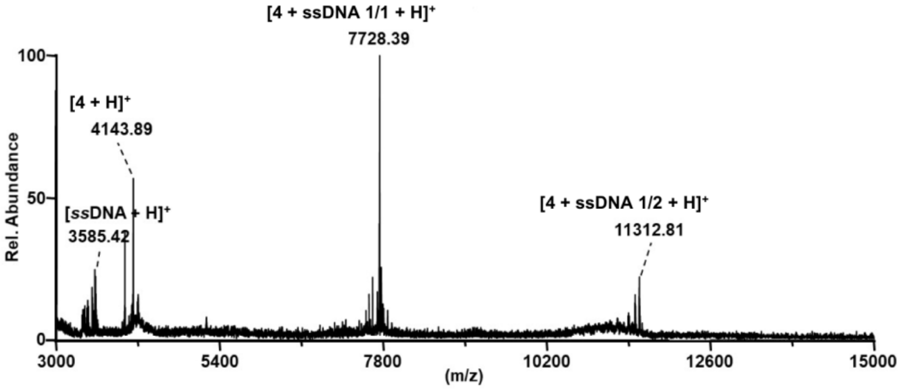

Figure S32. MALDI-TOF mass spectrum of the complex between **4** and 5’-AAGCCCGCCCAA-3’ (ssDNA).

	Theoretical average mass (g/mol)	Experimental average mass (g/mol)	Mass accuracy (ppm)
[**5** + H]$^+$	5505.51	5505.52	2
[**5** + ssDNA 1/1 + H]$^+$	9089.91	9089.98	8
[**5** + ssDNA 1/2 + H]$^+$	12674.31	12674.44	10

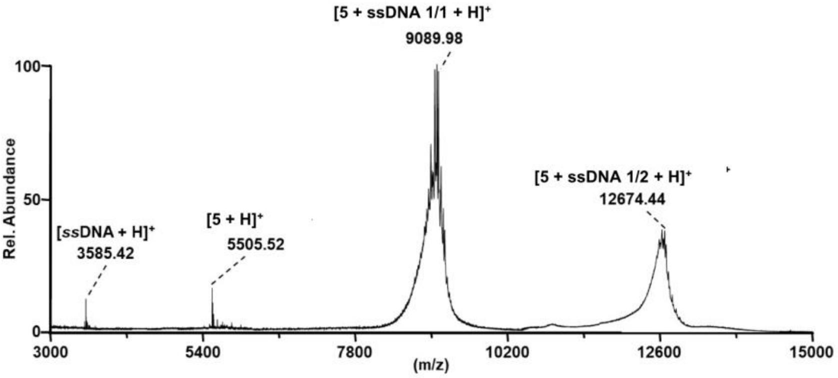

Figure S33. MALDI-TOF mass spectrum of the complex between **5** and 5’-AAGCCCGCCCAA-3’ (ssDNA).

	Theoretical average mass (g/mol)	Experimental average mass (g/mol)	Mass accuracy (ppm)
[**6** + ssDNA 1/1 + H]$^+$	7575.52	7575.41	15
[**6** + ssDNA 1/2 + H]$^+$	11159.92	11160.10	16

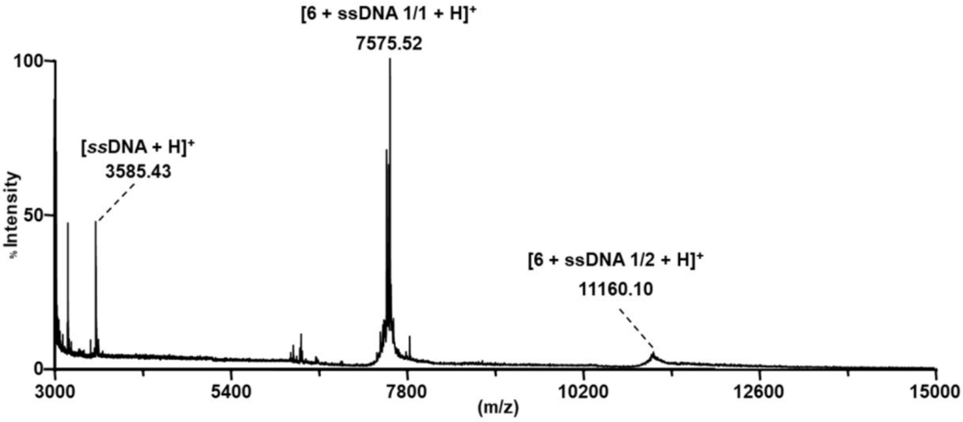

Figure S34. MALDI-TOF mass spectrum of the complex between **6** and 5’-AAGCCCGCCCAA-3’ (ssDNA).

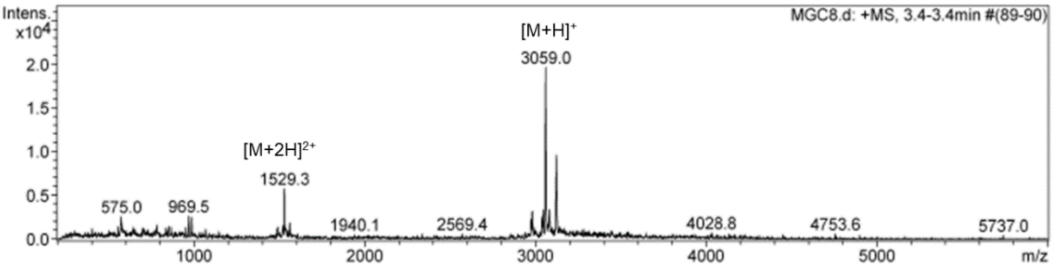

Figure S35. ESI-MS spectrum of **7**.

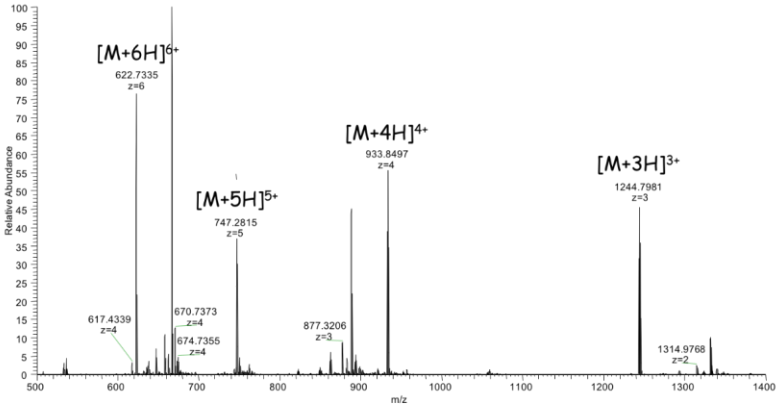

Figure S36. ESI mass spectrum of **8**.

	Theoretical average mass (g/mol)	Experimental average mass (g/mol)	Mass accuracy (ppm)
[**9** + H]+	5302.14	5303.15	1
[**9** + ssDNA 1/1 + H]+	8887.54	8887.58	4
[**9** + ssDNA 1/2 + H]+	12471.95	12472.04	8

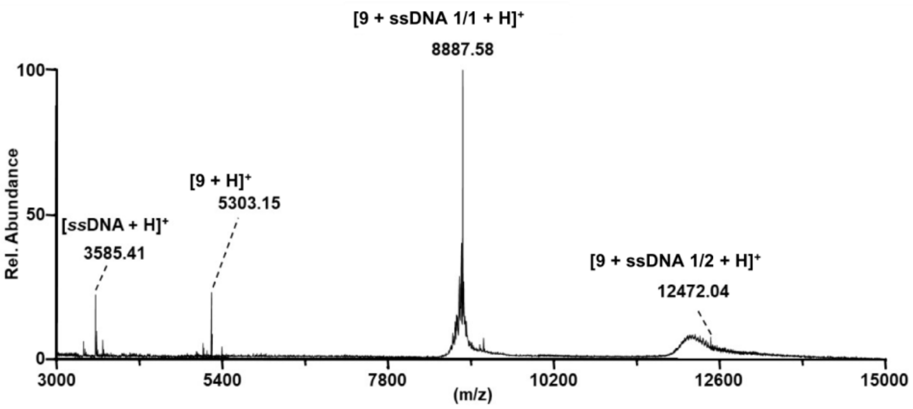

Figure S37. MALDI-TOF mass spectrum of the complex between **9** and 5’-AAGCCCGCCCAA-3’ (ssDNA).

	Theoretical average mass (g/mol)	Experimental average mass (g/mol)	Mass accuracy (ppm)
[**10** + H]+	5996.63	5996.67	5
[**10** + ssDNA 1/1 + H]+	9581.04	9581.15	12
[**10** + ssDNA 1/2 + H]+	13165.44	13165.65	16

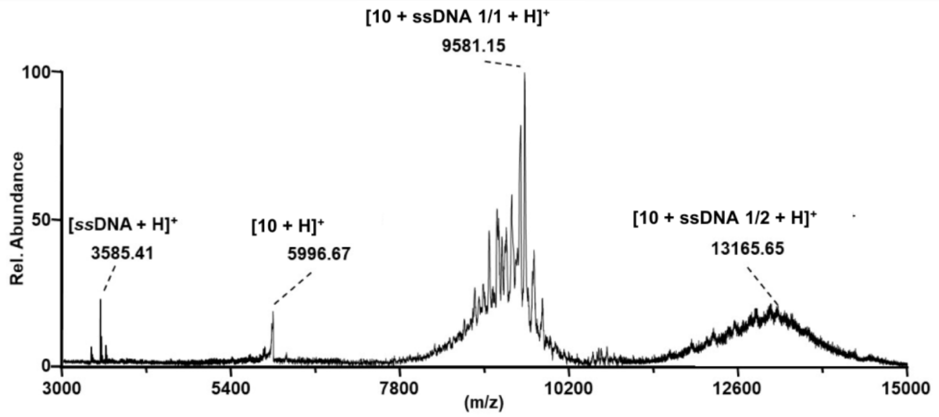

Figure S38. MALDI-TOF mass spectrum of the complex between **10** and 5'-AAGCCCGCCCAA-3' (ssDNA).

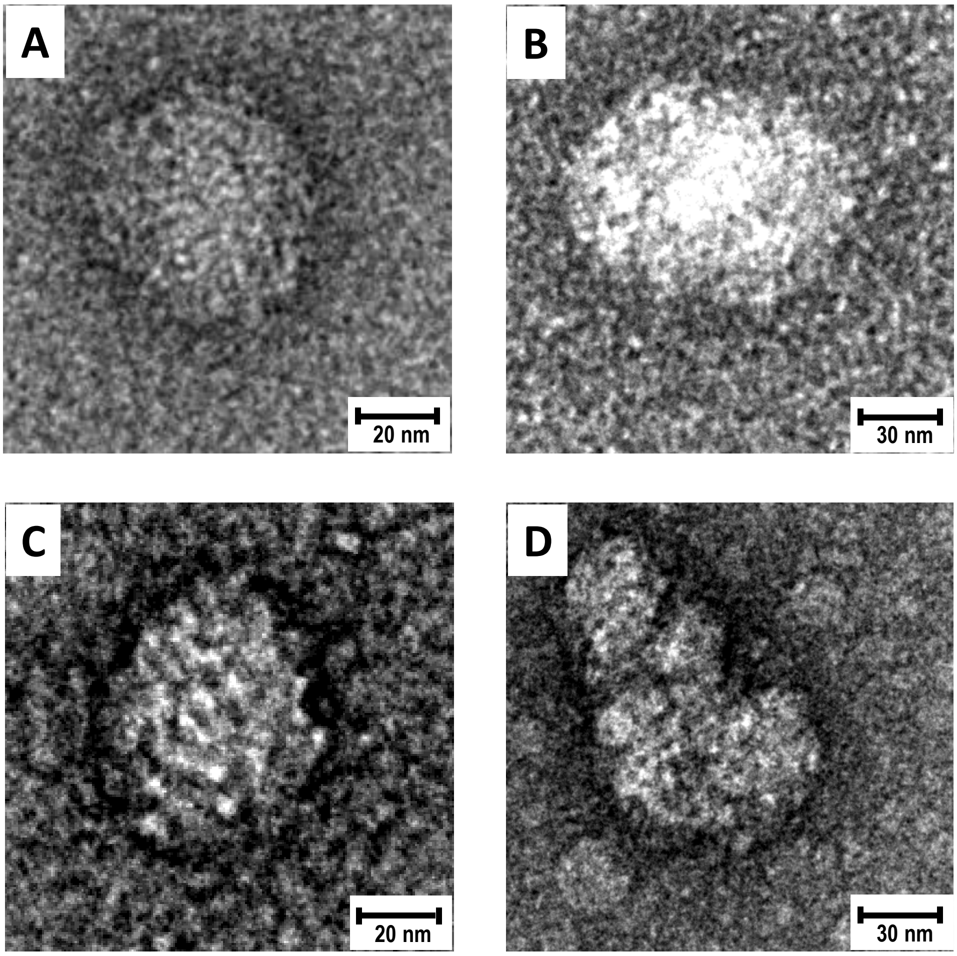

Figure S39. High magnification TEM images of representative nanocomplexes formulated with the pDNA and the MM-MNPs **1** (A), **4** (B), **7** (C) and **8** (D) showing the diferences in their ultrathing structures.

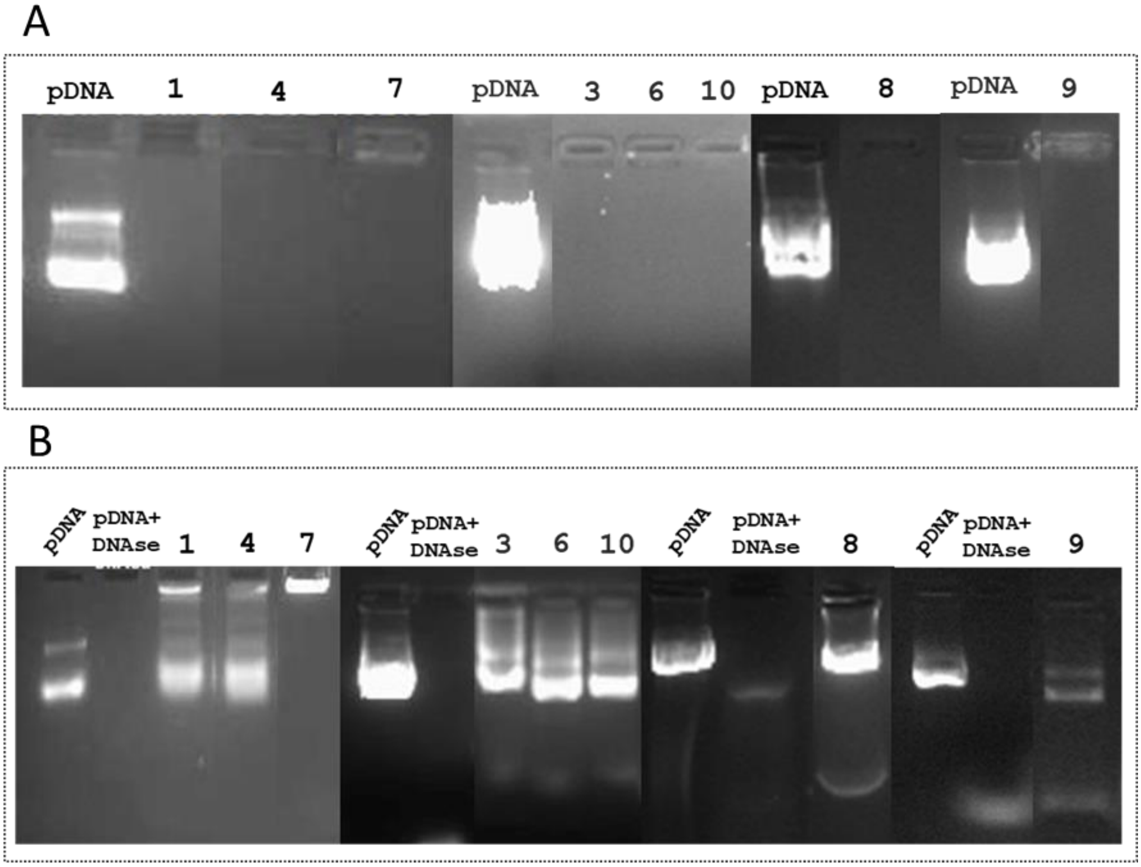

Figure S40. EMSA gels for CTplexes formulated with compounds **1**, **3**, **4** and **6-10** at N/P 10, before (A) and after treatment with DNAse I and subsequent dissociation of the complexes with SDS (B). Naked pDNA and ethidium bromide were used as the control and staining reagent, respectively. Lanes for compounds that were included in the same gel are grouped (i.e., from left to right, **1**, **4** and **7**; **3**, **6** and **10**; **8**; and **9**) and the lane for the pDNA (and pDNAse in B) reference from the corresponding gel is also included.

Detailed protocols for the computational assessment of CTplex assembly from the C_1L_2 or C_2L_1 MM-MNPs 1 or 7 and DNA

Molecular mechanics (MM) and molecular dynamics (MD) calculations: The Sybyl X-2.0 (SYBYL-X 2.0; Tripos Associates; St. Louis, MO, 2012) and the Tripos Force Field were used for all calculations.[6] We obtained charges for the MM-MNPs by MOPAC (AM1) using the Gaussian program. For initial studies in the absence of DNA, the NH_3^+ substituents were neutralized by chloride ions bound to nitrogen by dummy bonds. Calculations were performed not only in vacuo but also in water. The Molecular Silverware algorithm (MS) and periodic boundary conditions (PBC) were used for solvation.[7] A relative permittivity $\varepsilon = 3.5$ (1.0) was used for electrostatic contributions *in vacuo* (presence of explicit water). Nonbonded cutoff distances were set at 12 Å. Optimizations were performed with the simplex algorithm, and the conjugate gradient was used as a termination method with gradients of 0.5 kcal/(mol Å) (3.0 kcal/(mol Å)) *in vacuo* (water).[8] The B-DNA fragments used to emulate the situation in the **1**/pDNA or **7**/pDNA nanocomplexes contained twelve nucleotides with a CGCGAATTCGCG sequence each. DNA fragment charges were derived by using the Gasteiger and Marsili method.[9]

To get a deeper insight on the interactions at play in the formation of **1**/pDNA and **7**/pDNA nanocomplexes, the stability of antiparallel dimers facing their hydrophobic domains, both in the bulk and in the confined space between DNA fragments, was investigated by molecular mechanics (MM) and molecular dynamics (MD) simulations conducted in explicit water. Such dimers can be considered as the basic elements of vector bilayers. In the first calculation, a molecule of **1** (or **7**) with all branches in the *all trans* conformation, in which the six (or twelve) amine termini were neutralized by chloride anions bound to the nitrogen atoms by dummy bonds, was located with the center of mass of the CT3 macroring (*o*) at the origin of a coordinate system and oriented along the *y* axis. Next, another neutral molecule of the vector oriented in the opposite *y* axis direction approached along this axis in the most favorable orientation. Structures generated by scanning the *o–o'* distances from 35 to 12 Å at 0.5 Å intervals, followed by solvation (Molecular Silverware algorithm – MS – and periodic boundary conditions – PBC) were used for optimization (gradient 1.5 kcal mol^{-1} $Å^{-1}$). The solvent was then removed and for the resulting supramolecular dimer the *o–o'* distance was decreased by 0.5 Å, solvated and optimized again; once the water was removed, this structure was used as a starting point for the next structure and so on, *i.e.*, every structure generated in the previous step was used as the starting one for the following step. Figure S41 illustrates this procedure for **1**-dimer formation; a similar protocol was followed in the case of **7**-dimer formation.

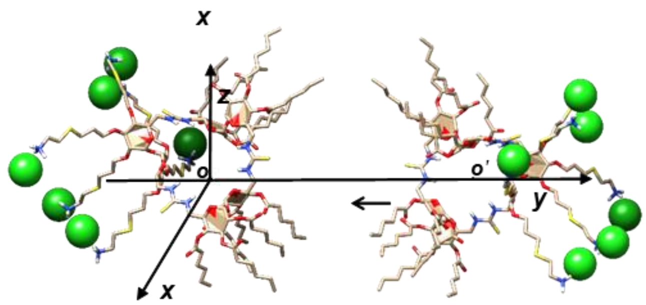

Figure S41. Coordinate system used for **1**-to-**1** (neutral form) approach along the *y* coordinate (chloride anion are depicted as green spheres, oxygen in red, nitrogen in blue, carbon in tan).

Figure S42 A depicts results of the total binding energy and electrostatics and van der Waals contributions as a function of the distance between the centers of mass of the two neutral units of the C_1L_2 MM-MNP **1** approaching along the *y* coordinate in the most favorable orientation. Total binding energies were negative and they monotonically decreased with distance, reaching the MBE structures at the shortest distance. The larger contributions came from van der Waals interactions. As shown in Figure S42 B, something similar occurred for total binding energy and electrostatics and van der Waals contributions as a function of the distance for the neutral dimer of the C_2L_1 MM-MNP **7**. Figure S40 A and B also depict the MBE structures for **1**-dimer and **7**-dimer. These dimeric structures, once the chloride atoms were removed (with net charge +12 esu for **1** or +24 esu for **7**) were employed in the next step for the approaching of two DNA fragments.

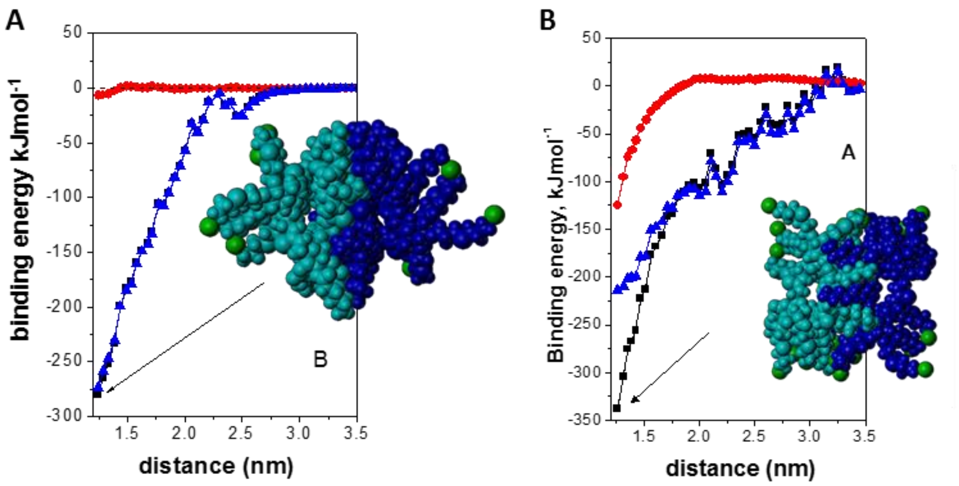

Figure S42. Total binding energy (black squares) and electrostatics (red circle) and van der Waals (blue triangle) contributions upon (A) **1**-to-**1** or (B) **7**-to-**7** (neutral form) approaching along the y coordinate from the positive side (see also Figure S39). Superimposed is the corresponding MBE dimer structure.

To study the stability in water of the (MM-MNP)$_2$-DNA$_2$ nanocomplexes, the MBE optimized charged corresponding (MM-MNP)$_2$ dimer (**1**-dimer or **7**-dimer) was positioned with its center of mass at the origin of a coordinate system and two DNA fragments were placed along different virtual *y*' axes with their major grooves oriented towards the cationic patches as depicted in Figure S43. Then, the DNA(1) and DNA(2) fragments were simultaneously approached to the dimer in 0.2 Å steps along the *y'* coordinate from *y'* = +25 to 9 Å. Each structure was solvated (Molecular Silverware algorithm – MS – and periodic boundary conditions – PBC) and used for optimization (MM) to obtain the corresponding **1**-dimer/(DNA)$_2$ and **7**-dimer/(DNA)$_2$ MBE structures (Figure S44 A and B). Such MBE structures were used as the starting conformations for 1.0 ns MD simulation in the presence of water following the strategy described earlier.[10] The MD histories for some key distances and the **1**-dimer/(DNA)$_2$ and **7**-dimer/(DNA)$_2$ structures averaged throughout the 1 ns MD trajectory are represented in Figure 5 in the manuscript.

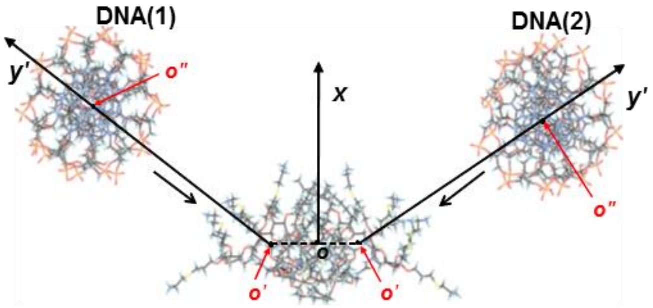

Figure S 43. Coordinate system used for DNA fragments approaching **1**-dimer along the *y'* virtual axes (*o"* is the center of mass of each DNA fragment and *o'* is the center of mass of each CT3 macrocycle in the vector; the origin of the Cartesian system, *o*, is placed in the center of mass of the dimer). An analogous system was used for DNA fragments approaching **7**-dimer.

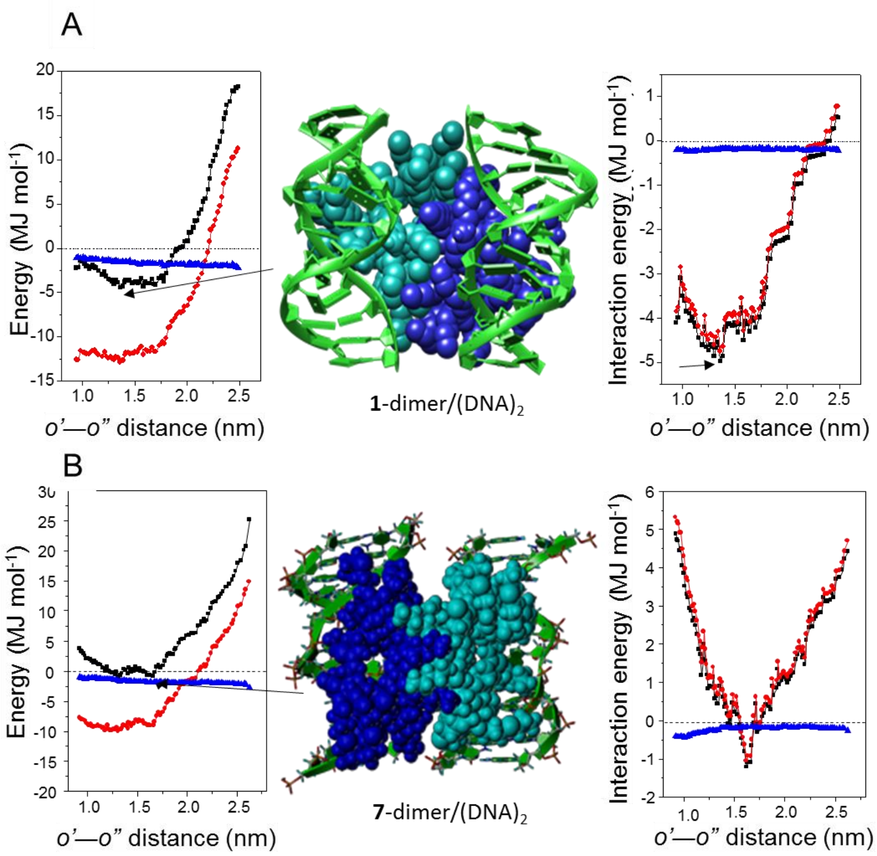

Figure S44. A) (left) Total energy (black) and electrostatics (red) and van der Waals (blue) contributions *versus* the average distance between the center of mass of each DNA and each vector **1** macroring (*o'*—*o*" distance); (right) Interaction energy and contributions (same colors) between the DNA (1) and DNA(2) fragments in the nanocomplex versus the same *o'*—*o*" average distance; (middle) **1**-dimer/(DNA)$_2$ MBE structure obtained from MM calculations. B) The same representations for **7** (DNA fragments in green; the individual monomer constituents are colored in light and deep blue).

Cell viability (MTT assay) for the C_1L_2 MM-NPs 1, 3, 5 and 7 and for the C_2L_1 MM-MNPs 7-10

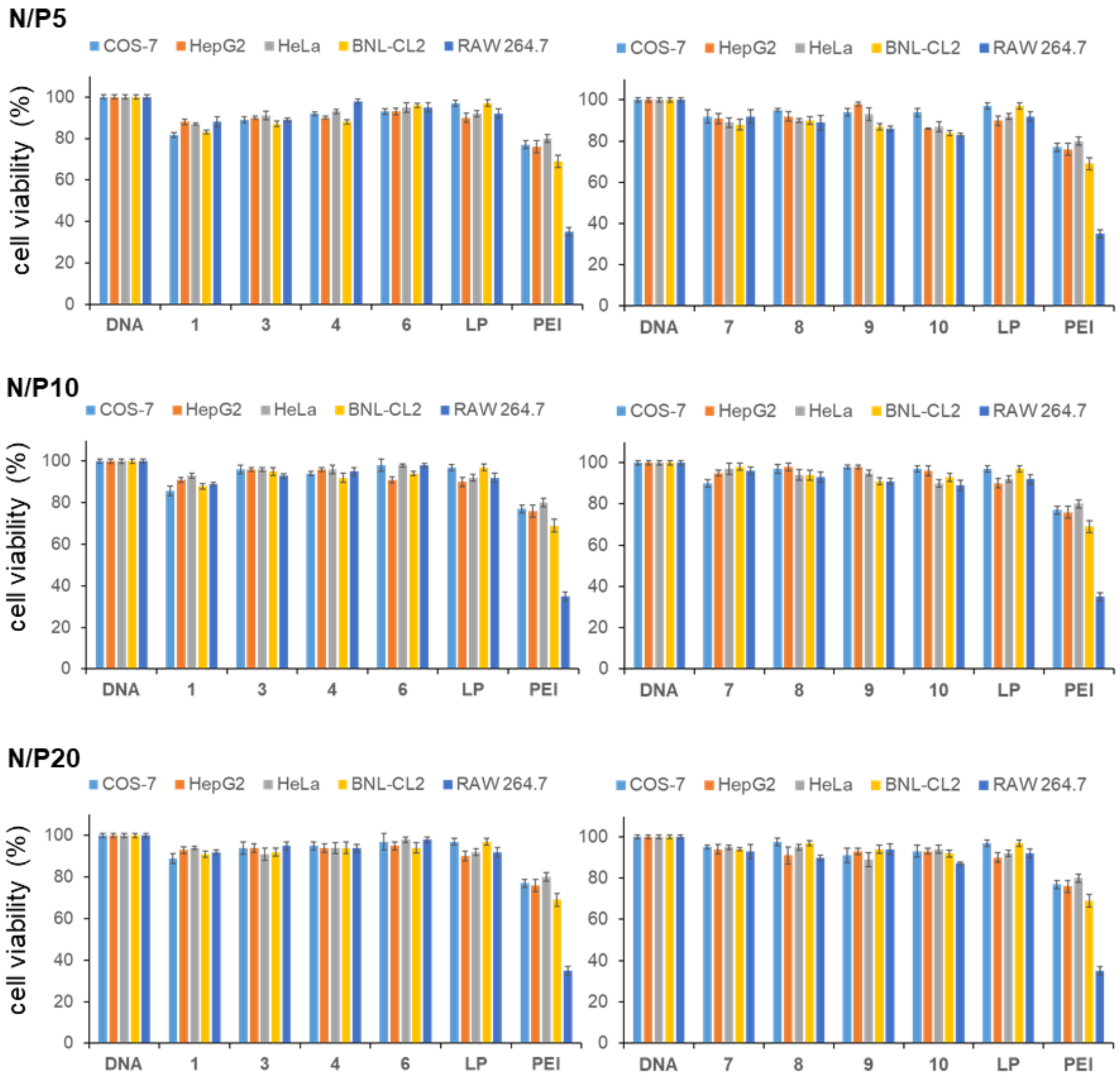

Figure S45. Cell viability (% relative to naked DNA; MTT assay) for the C_1L_2 MM-NPs **1**, **3** ,**4** and **6** (left) and for the C_2L_1 MM-MNPs **7-10** (right) at N/P 5 (upper panel) 10 (middle panel) and 20 (lower pamel). Data for Lipofectamine (LP) and PEI at N/P 10 are included as references.

UV-Vis and circular dichroism spectra of the MM-MNPs 1 and 7 and their complexes with ctDNA

UV-vis: The UV-Vis spectra of **1** showed intense bands centered at 222 and ~247 nm (ε_{247nm}=nm 31,700 L·mol^{-1}·cm^{-1}). Compound **7** displayed bands at 222 and ~241 nm ($\varepsilon_{241\ nm}$=23,400 L·mol^{-1} ·cm^{-1}). Absorbances varied linearly with concentration for both cyclotrehalans (Figure S43). In the corresponding circular dichroism spectra **1** exhibited a relatively intense negative Cotton effect centered at 245-247 nm; this band appeared positive for **7** (Figure S46). Both spectra showed small negative ellipticity signals centered at approximately 275 nm and 290 nm for **1** and **7** respectively. Absolute values of ellipticity measured at 243 nm, experienced a linear increase with concentration in both cases (Figure S47). The data support that in the range of concentration used in the biological assays **1** and **7** did not self-associated, in agreement with DLS and TEM results.

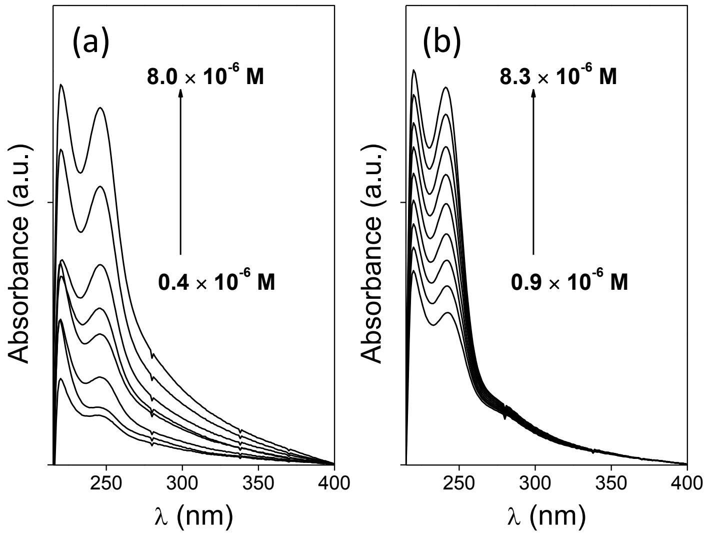

Figure S46. UV-Vis spectra dilute solutions of **1** and **7** in HEPES buffer at 25 °C: (a) **1** at 0.4, 0.8, 1.6, 2.4, 3.2, 4.0, 6.0 and 8.0 × 10^{-6} M and (b) **7** at 0.9, 2.8, 3.7, 4.6, 5.6, 6.5, 7.4 and 8.3 × 10^{-6} M concentrations.

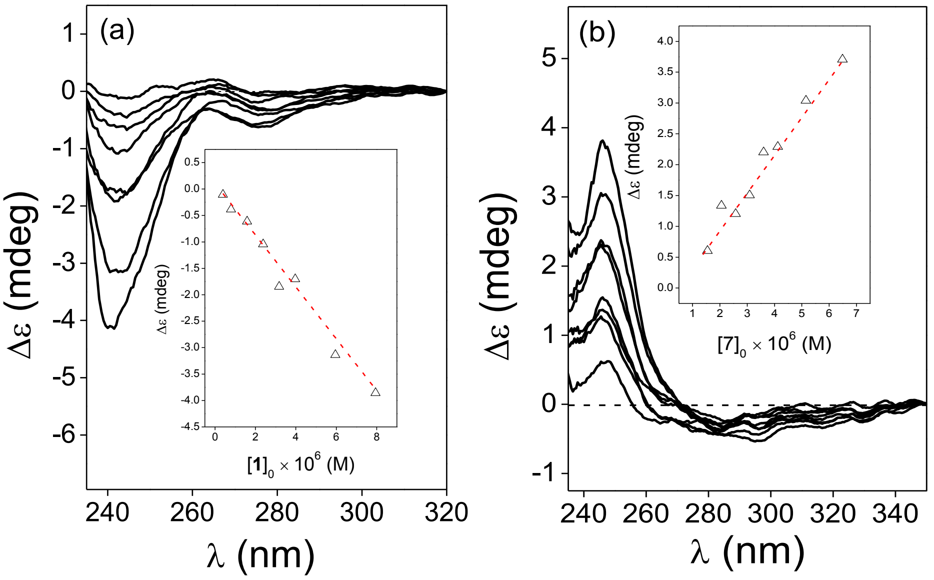

Figure S47. Normalized CD spectra as ellipticties (mdeg), of (a) **1** at 0.4, 0.8, 1.6, 2.4, 3.2, 4.0, 5.96 and 8.0 × 10^{-6} M concentrations and (b) **7** at 1.5, 2.0, 2.6, 3.1, 3.6, 4.1, 5.1 and 6.5 × 10^{-6} M concentrations in the 235-350 nm range at 25 °C. Inset: Plots of the ellipticity (Δε, mdeg) measured at 243 nm versus concentration.

Circular dichroism: Solutions of the C_1L_2 MM-MNP **1** and of the C_2L_1 analogue **7** were prepared in HEPES buffer (pH 7.4, 20 mM). A calf-thymus DNA (*ct*DNA, Aldrich) in HEPES buffer (1 mg mL^{-1}) was used as a stock solution that was kept in the freezer. The final *ct*DNA solutions were prepared daily by dilution. The concentration (per pair of bases) was determined by UV-Vis spectroscopy (ε_{260nm} = 13,200 M-1·cm^{-1} by using a UVIKON 941 spectrophotometer).[11] Measurements were performed on a JASCO J-715 spectropolarimeter. The recorded spectra were the average of three scans taken from 320 (or 350) to 235 nm at the speed of 20 $nmmin^{-1}$ with a time response of 0.125 s in quartz cells of 5 cm path-length. The sensitivity and resolution were 20 mdeg and 10 nm respectively. Measurements were made at 25 ºC. Molar ellipticity was defined as:

$$[\theta] = \Delta\varepsilon/[L]\cdot l \ (mg\cdot deg\cdot cm^2\cdot mol^{-1})$$

where θ is the ellipticity in mdeg, [L] is the ligand concentration (mol·cm^{-3}) and l is the optical path in cm.

The circular dichroism of ctDNA showed the typical positive and negative bands centered at around 278 and 245 nm, which are characteristic of the secondary structure of a DNA B-form

(Figure S45). These bands are usually attributed to base stacking and right-handed polynucleotide helicity, respectively.[12-14] Although bands for vectors **1** and **7** overlap with those observed for ctDNA, this overlapping is less pronounced for the band located at the smaller energy (275nm). For this reason, this band was selected for measuring the ellipticity changes (Δε) in the presence of ctDNA. To exclude the influence of the concentration of **1** and **7** on Δε, the molar ellipticities ([θ]) as a function of the N/P ratios were evaluated and their changes were represented vs N/P ratios. The change (decreasing) observed **1** and **7** demonstrate the presence of interaction between both cyclotrehalans and ctDNA (Figure S48).

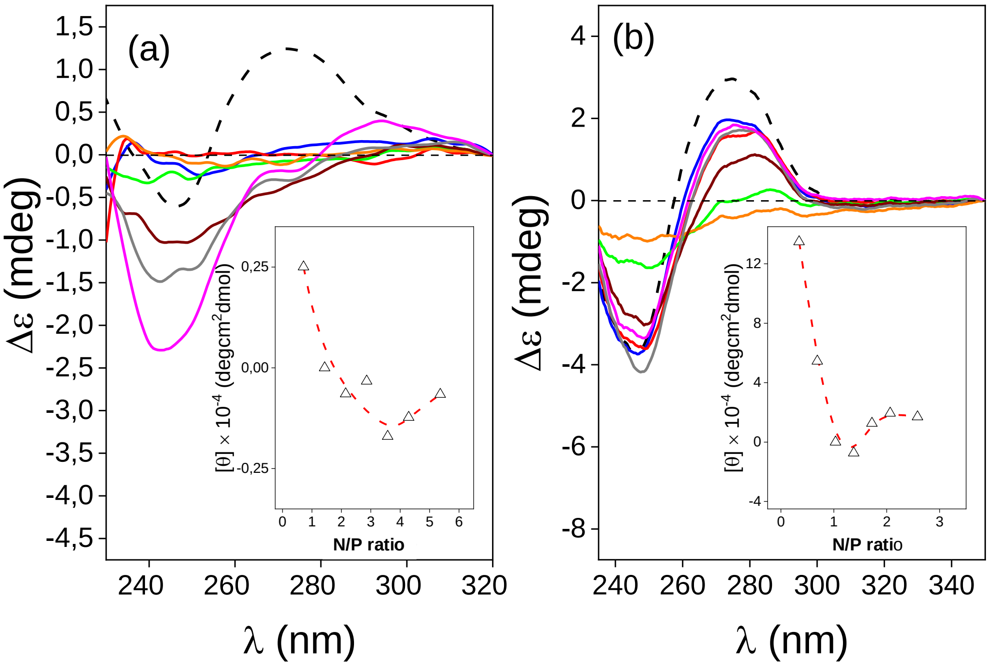

Figure S48. Circular dichroism spectra as ellipticties (mdeg), in the 235-320 (350) nm region, for **1** and **7** buffer solutions of different concentration in the presence of *ct*DNA at a fixed concentration. (a) For **1** at 0 (dashed line), 0.7, 1.4, 2.1, 2.9, 3.6, 4.3 and 5.4 N/P ratios and a fixed [*ct*DNA] =3.0 × 10^{-6} M; (b) for **7** at 0 (dashed line), 0.3, 0.7, 1.0, 1.4, 1.7 2.1 and 2.6 N/P ratios and a fixed [*ct*DNA]= 5.0 × 10^{-6} M concentration. Inset are the changes in the molar ellipticity (equation 1) as a function of the N/P ratios measured at 275 nm for both cyclotrehalans.

References

[1] A. Díaz-Moscoso, L. Le Gourrierec, M. Gómez-García, J. M. Benito, P. Balbuena, F. Ortega-Caballero, N. Guilloteau, C. Di Giorgio, P. Vierling, J. Defaye, C. Ortiz Mellet, J. M. García Fernández, *Chem. Eur. J.* **2009**, *15*, 12871-12888.

[2] J. Rodríguez Lavado, S. E. Sestito, R. Ciguetti, E. M. Aguilar Moncayo, A. Oblak, D. Lainscek, J. L. Jiménez Blanco, J. M. García Fernández, C. Ortiz Mellet, R. Jerala, V. Calabrese, F. Peri, *J. Med. Chem.* **2014**, *57*, 9105-9123.

[3] D. Manzanares, I. Araya-Durán, L. Gallego-Yerga, P. Játiva, V. Márquez-Miranda, J. Canan, J. L. Jiménez Blanco, C. Ortiz Mellet, F. D. González-Nilo, J. M. García Fernández, V. Ceña, *Nanomedicine* **2017**, *12*, 1607-1621.

[4] J. M. García Fernández, C. Ortiz Mellet, J. L. Jiménez Blanco, J. Fuentes Mota, A. Gadelle, A. Coste-Sarguet, J. Defaye. *Carbohydr. Res.* **1995**, *268*, 57-71.

[5] A. I. Carbajo-Gordillo, J. L. Jiménez Blanco, J. M. Benito, H. Lana, G. Marcelo, C. Di Giorgio, C. Przybylski, H. Hinou, V. Ceña, C. Ortiz Mellet, F. Mendicuti, C. Tros de Ilarduya, J. M. García Fernández, *Biomacromolecules* **2020**, *21,* 5173-5188.

[6] M. Clark, R. D. Cramer III, N. Van Opdenbosch, *J. Comput. Chem.* **1989**, *10*, 982-1012.

[7] M. J. González-Álvarez, J. Vicente, C. Ortiz Mellet, J. M. García Fernández, F. Mendicuti, *J. Fluoresc.* **2009**, *19*, 975-988.

[8] M. Blanco, *J. Comput. Chem.* **1991**, *12*, 237-247.

[9] a) J. Gasteiger, M. Marsili, *Tetrahedron Lett*. **1978**, 3181-3184; b) J. Gasteiger, M. Marsili, *Tetrahedron*, **1980,** *36*, 3219-3228.

[10] L. Gallego-Yerga, M. J. González-Álvarez, N. Mayordomo, F. Santoyo-González, J. M. Benito, C. Ortiz Mellet, F. Mendicuti, J. M. García Fernández, *Chem. Eur. J.* **2014**, *20*, 6622-6627.

[11] A. W. McConnaughie, T. C. Jenkins, *J. Med. Chem.* **1995**, *38*, 3488-3501.

[12] M. Ardhammar, B. Nordén, T. Kurucsev, *Circular dichroism: principles and applications*, Wiley-VCH, Weinheim, **2000**, p. 741-768.

[13] W. C. Johnson, *Circular dichroism: principles and applications*, Wiley-VCH, Weinheim, **2000**, p. 703-718.

[14] T. Šmidlehner, I. Piantanida, G. Pescitelli, *Beilstein J. Org. Chem.* **2018**, *14*, 84-105.
